# Molecular Dynamics Simulations of RNA Stem-Loop Folding Using an Atomistic Force Field and a Generalized Born Implicit Solvent

**DOI:** 10.1021/acsomega.5c05377

**Published:** 2025-10-26

**Authors:** Tadashi Ando

**Affiliations:** † Department of Applied Electronics, Tokyo University of Science, 6-3-1 Niijuku, Katsushika-ku, Tokyo 125-8585, Japan; ‡ Research Institute for Science and Technology, Tokyo University of Science, 2641 Yamazaki, Noda, Chiba 278-8510, Japan

## Abstract

Accurate modeling of the structural dynamics of ribonucleic acid (RNA) molecules, including common stem-loop motifs, remains challenging. This study presents *de novo* folding simulations of a diverse set of 26 RNA stem-loops, ranging from 10 to 36 residues, with and without bulges or internal loops, starting from their extended conformations. These simulations employed conventional molecular dynamics using an atomistic force field extensively refined by the Shaw group ( TanD.,  Proc. Natl. Acad. Sci. U.S.A. 2018, 115, E1346−E1355, 10.1073/pnas.1713027115 29378935 PMC5816156) and an implicit solvent model developed by the Simmerling group ( NguyenH.,  J. Chem. Theory Comput. 2015, 11, 3714−3728, 10.1021/acs.jctc.5b00271 26574454 PMC4805114). The 18 stem-loops without bulges or internal loops were folded into their respective structures, retaining all native base pairs in the stem regions. For most of these models, root mean square deviation (RMSD) values relative to experimentally determined structures were <2 Å for stem regions and <5 Å for the molecules. Furthermore, five of the eight stem-loops containing bulges or internal loops were successfully folded into structures with all respective native base pairs in the stem regions. The models initially formed stems directly connected to hairpin loops, followed by the remaining duplex stems between the bulge or internal loop and the terminal. The RMSD values for these structures were 0.9–4.5 Å for the stem regions and 2.8–8.3 Å for the molecules. The RMSD values for the loop regions were approximately 4 Å for all models. Accurate modeling of loop structures remains challenging in simulations using the implicit solvent model. However, our success in recapitulating the RNA stem folding of fundamental stem-loop motifs represents a pivotal step toward enhancing reliable and accurate modeling of RNA structural dynamics.

## Graphical abstract

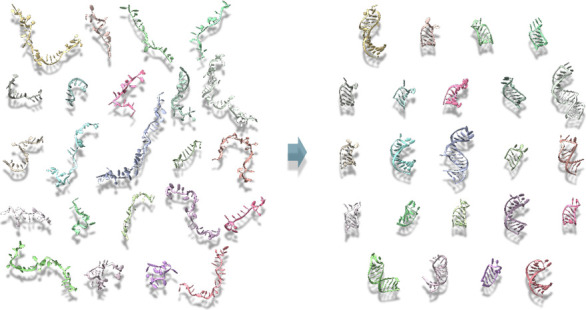

## Introduction

Ribonucleic acids (RNAs) are important molecules in living systems. They are versatile and involved in numerous biological processes that are essential for the maintenance, regulation, and processing of genetic information. RNA-based medicine has experienced recent significant advances with the development of numerous applications, including therapeutics, vaccines, and diagnostics.^1^ RNA molecules are linear polymers composed of four nucleotides (nt) with nitrogenous bases: adenine (A), uracil (U), guanine (G), and cytosine (C). These molecules can adopt a variety of secondary structures, including various structural elements, such as stems, loops, internal loops, bulges, and pseudoknots, which are defined by the canonical Watson–Crick base pairs (A-U and G-C) and the noncanonical wobble base pair (G-U).^2,3^ Interactions between distinct secondary structural elements form more complex tertiary structures. As with other biomolecules, the biological functions of RNAs are closely linked to their structures and dynamics. Deep learning-based methods have revolutionized the research field of RNA structure prediction with increasingly higher accuracy and efficiency.^4–6^ However, the conformational dynamics and flexibility of RNA are inherently coupled with its functions, as observed in riboswitches,^7^ ribozymes,^8^ and RNA–protein interactions.^9^ Therefore, developing methods to efficiently and accurately model RNA structures and dynamics will significantly impact RNA biology and medicinal chemistry.

All-atom molecular dynamics (MD) simulations, which use physics-based potentials, are powerful tools that can be used to investigate the relationships among the structures, dynamics, and biological functions of biomolecules.^10^ MD simulations provide the energetics of interactions and conformational dynamics of biomolecules in atomic detail and at fine temporal resolution.^10^ The AMBER RNA force field has been widely used for MD simulations of atomistic RNA systems.^11–14^ Currently, bsc0χ_OL3_ is the standard force field used for RNA systems in AMBER,^15,16^ hereafter called the AMBER-OL3 RNA force field. Since the AMBER-OL3 force field was parametrized in 2011, significant efforts have been made to improve its accuracy.^11–14^ Steinbrecher et al. modified the phosphate Lennard-Jones parameters with the aid of a thermodynamic cycle consisting of experimentally determined p*K*_a_ values, solvation energies from free energy calculations, and quantum mechanical (QM) calculations.^17^ Although originally developed for bioinorganic phosphates, these parameters improved the quantitative agreement between MD-simulated conformational ensembles of r­(GACC) and r­(CCCC) tetranucleotides and experimental NMR data.^18,19^ Refinements of backbone and glycosidic torsion parameters based on QM calculations have been performed.^20,21^ Chen and García adjusted the Lennard-Jones parameters for base–base and base–water interactions to weaken base stacking, which is overstabilized in the original force field.^22^ Kührová et al. introduced structure-specific local correction potential selectively modifying native hydrogen bonds of RNA, denoted as gHBfix, to improve the performance of AMBER RNA force fields while minimizing undesired side effects.^23,24^ The same group proposed a scheme for automatically adjusting gHBfix weights, and the resulting new parameter set was used in MD simulations, generating a structural ensemble of tetraloop RNAs that were in better agreement with experiments.^25^ Tan et al. introduced an extensive revision of electrostatic, van der Waals, and torsional parameters of the AMBER RNA force field based on QM calculations and existing experimental information to more accurately reproduce the energetics of nucleobase stacking, base pairing, and key torsional conformers.^26^ This force field (called the DESRES-RNA force field hereafter) combined with the TIP4P-D water model^27^ could effectively reproduce the structural and thermodynamic properties of various RNA systems. Specific adjustments for CH···O interactions were also proposed to improve RNA simulations.^28,29^ In a recent study, Mlýnský et al. conducted a comprehensive evaluation of various RNA force fields, including a polarizable force field, using a UUCG tetraloop as a benchmark system.^30^ They concluded that substantial hurdles remain in achieving reliable and accurate modeling of the diverse structural dynamics of RNA systems.^30^

In addition to the remarkable improvements in RNA force field accuracy in recent years, advances in computational resources and algorithms have greatly enhanced our ability to simulate RNA dynamics on various time scales. RNA dynamics encompass a wide range of time scales, reflecting the rugged free energy landscape of RNA folding, unfolding, and interactions. Depending on the specific RNA sequence and environmental conditions, these time scales can vary from microseconds to seconds. Recent experimental studies have shown that simple stem-loop folding also occurs on time scales ranging from tens of microseconds to several seconds.^31–36^ To tackle these long-time-scale dynamics by MD simulations, enhanced sampling techniques, such as replica-exchange MD (REMD) and metadynamics, are widely used. In parallel to enhanced sampling techniques, the development of optimized algorithms for the effective use of graphical processing units (GPUs) and single-instruction multiple-data (SIMD) architectures has significantly accelerated MD simulations, enabling the exploration of biomolecular dynamics at microsecond time scales in explicit solvents.^37,38^ A specialized supercomputer, named ANTON, has further extended MD simulations of RNA in explicit solvent molecules to hundreds of microseconds.^26^

Implicit solvent models can also be used to speed up atomistic simulations by approximating the explicit solvent as a continuum.^39,40^ A drastic reduction in the number of particles in a simulation system can reduce the computational cost. In addition, the low solvent viscosity in implicit solvent simulations accelerates the rate of conformational sampling compared with explicit solvent simulations.^41,42^ The generalized Born (GB) model, which was first introduced by Still et al. in 1990,^43^ is the most widely used implicit solvent model for atomistic MD simulations for biomolecules.^44^ Although the GB model is less realistic than an explicit solvent model, its accuracy has been significantly improved,^44^ and GPU-accelerated GB calculations have also been implemented.^45^ Nguyen et al. optimized the parameters of the original GB-neck model^46^ to reproduce more accurate Poisson–Boltzmann solvation energies for a broad range of peptide and protein systems.^47^ The GB-neck2 model combined with the AMBER ff14SBonlysc protein force field successfully simulated the folding of 16 proteins with diverse topologies.^38^ Notably, the AMBER ff14SBonlysc force field combined with the GB-neck2 model not only folds more proteins but also provides a better balance of different secondary structures than ff14SB combined with the explicit TIP3P water model.^48^ The GB-neck2 model parameters were refined for nucleic acids, and the resulting parameter sets enabled the successful folding of small DNA and RNA hairpins to near native structures.^49^

While remarkable advances have been made in RNA force fields and sampling techniques, simulating RNA folding, even for secondary structures, remains challenging. Chen and García reported that three hyperstable 8 nt RNA tetraloops folded into the structure with a non-hydrogen root mean square deviation (RMSD) of 1–3 Å from their experimental structures, where they used temperature-REMD starting from their unfolded states and an optimized force field based on AMBER-99 with the TIP3P explicit solvent.^22^ Sponer and Bussi performed an extensive set of folding simulations for two tetranucleotides and one 8 nt tetraloop using various enhanced sampling techniques and the gHBfix-applied AMBER RNA force field with explicit solvent models to show an improved agreement between experimental data and conformational ensembles from simulations compared with the original AMBER force field.^24,25^ The DESRES-RNA force field combined with the TIP4P-D water model can simulate the reversible folding of three tetraloops (two 10 nt and one 14 nt) using a simulated tempering enhanced sampling method. These simulations successfully sampled conformations with an overall non-hydrogen RMSD of 1–2 Å from their experimentally determined structures at low temperatures.^26^ With the same force field, magnesium ion-dependent folding of an 8 nt tetraloop was observed in a conventional MD simulation.^50^ An implicit solvent model captured the folding of three tetraloops (two 12 nt and one 14 nt) using deep boosted enhanced sampling MD with the DESRES-RNA force field and the GB-neck2 model.^51^ Linzer et al. reported unfolding/refolding events of stem regions for several stem-loops ranging from 10 to 27 nt in conventional MD simulations using the AMBER-OL3 force field and the GB-neck2 model, close to the predicted melting temperatures.^52^

Here, we present the results of conventional MD simulations for 26 RNA stem-loops, ranging from 10 to 36 nt, with and without bulges or internal loops, starting from their extended conformations. Similar to MD simulations of protein systems, the folding simulation of RNA molecules serves as a stringent test to assess whether current molecular mechanics force fields are sufficiently accurate to enable long-time-scale MD simulations, as a powerful tool for characterizing large conformational changes in RNA.^53^ Among these examined models, 23 RNA molecules were successfully folded into structures with native base pairs and non-hydrogen RMSD values of <2 and 4 Å for the stem and loop regions, respectively. These structures were generally stable and formed dominant clusters in their trajectories. The loop structures sampled in their folded states were less accurate than the stem regions, and further improvement of the force field parameters is required. We expect that this force field and GB model combination will facilitate future MD simulation studies on the complex dynamics of a wide range of RNA systems, such as tertiary structure folding, conformational changes of riboswitches, and kissing-loop complexes.

## Models and Simulation Methods

### RNA Stem-Loop Models

A total of 26 RNA stem-loops with varying sequences and lengths were simulated, 15 of which corresponded to the models previously examined by Linzer et al.^52^ Their sequences, secondary structures, and Protein Data Bank (PDB) IDs are listed in Figure 1. Hereafter, all models are referred to using their PDB IDs. The stem-loops without bulges or internal loops (lengths in parentheses) are as follows (Figure 1A): 1R4H^54^ (10 nt), 1IDV^54^ (10 nt), 1ZIH^55^ (12 nt), 1I46 ^56^ (13 nt), 1ESH^57^ (13 nt), 1F85 ^58^ (14 nt), 1FHK^59^ (14 nt), 2EVY^60^ (14 nt), 2KOC^61^ (14 nt), 2Y95 ^62^ (14 nt), 1OQ0 ^63^ (15 nt), 1XWP^64^ (15 nt), 1JTW^65^ (16 nt), 1KKA^66^ (17 nt), 2RPK^67^ (20 nt), 1SZY^68^ (21 nt), 1PJY^69^ (22), and 2LDL^70^ (27 nt). The stem-loops with bulges or internal loops are as follows (Figure 1B): 1ESY^71^ (19 nt), 17RA^72^ (21 nt), 2KF0 ^73^ (24 nt), 1ANR^74^ (29 nt), 2JWV^75^ (29 nt), 1R2P^76^ (34 nt), 1R7W^77^ (34 nt), and 1N8X^78^ (36 nt). The stem-loop structures of the 26 RNAs were solved by NMR. The loops ranged in length from 3 to 8 nt, and the bulges and internal loops ranged in length from 1 to 6 nt. A5 and U14 in 1ESY and A6 and U24 in 1ANR can form base pairs, but they were not formed in the NMR structures. Six stem-loop models (1F85, 2EVY, 1OQ0, 1JTW, 17RA, and 1N8X) contained the G-U wobble base pair. 2LDL and 2KF0 contained the noncanonical C-A wobble base pair; and 1N8X had a G-A mismatched base pair. Hereafter, we refer to stem-loops without a bulge or internal loop as “class I” stem-loops and those with a bulge or internal loop as “class II” stem-loops.

**Figure 1:**
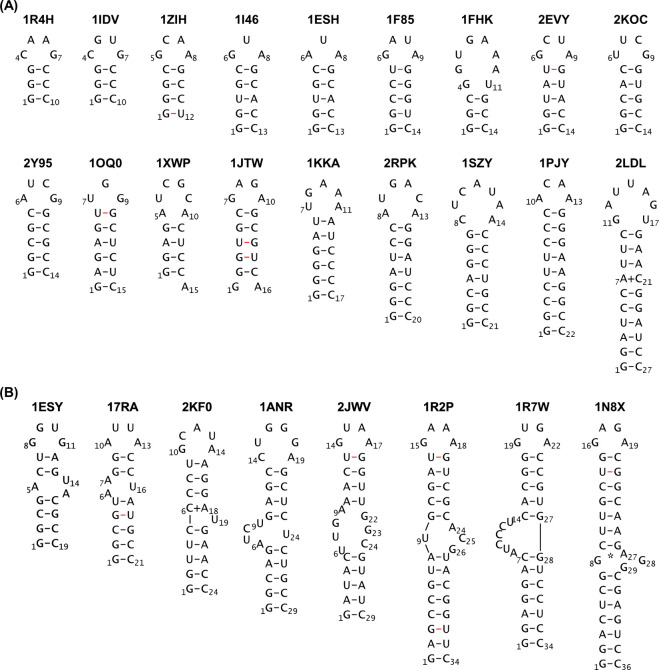
Secondary structures of 26 RNA stem-loops simulated in this study and their corresponding Protein Data Bank IDs. (A) Eighteen class I stem-loops without bulges or internal loops. (B) Eight class II stem-loops with bulges or internal loops. The G-C/A-U Watson–Crick and the G-U wobble base pairs are represented by black and red lines, respectively. Wobble base pairs A7-C21 in 2LDL and C6-A18 in 2KF0 were observed by NMR spectroscopy and are denoted by plus signs (+). A G8-A27 mismatch base pair in 1N8X was observed by NMR spectroscopy and is denoted by an asterisk (*).

### Implicit Solvent MD Simulations for Stem-Loops

All simulations were performed using the *pmemd.cuda* module of AMBER22 ^79^ with an NVIDIA GeForce GTX 2080 Ti or RTX 3090 GPU. The DESRES-RNA force field^26^ and the GB-neck2 implicit solvent model^49^ with the mbondi3 ^47^ intrinsic radii set for nucleic acids were used, where mbondi3 and mbondi2 ^80^ are equivalent for nucleic acid simulations. The *tleap* module of AMBER22 generated extended conformations of RNA molecules as initial structures for the MD simulations, which were subjected to energy minimization for 1000 steps, consisting of 500 steps of the steepest descent followed by 500 steps of the conjugate gradient. Production simulations were performed at 298 K with a time step of 2 fs using the SHAKE algorithm^81^ to constrain the bond lengths involving hydrogen atoms. The temperature was controlled using a Langevin thermostat^82^ with a collision frequency of 1 ps^–1^. The salt concentration in the GB model was set at 0.15 M. The nonpolar contribution to the solvation free energy, which was approximated as a linear function of the solute surface area, was not computed in this study. No cutoff value was used for the calculations of nonbonded interactions and the effective Born radii. The simulation time was 8 μs for the 2EVY and 2LDL class I stem-loops as well as the class II stem-loops and 4 μs for the remaining class I stem-loops.

Analysis was performed using the *cpptraj* program in AmberTools24.^83,84^ For reference structures, the first NMR structural models were used if multiple structural models were deposited. For 1I46, 1ESH, 1XWP, and 2RPK, minimized average structures were used as the reference structures. The RMSD was calculated for the heavy atoms of the molecules. Base pairs were detected by using the *nastruct* command in *cpptraj* with the default parameters. Although the *nastruct* judged the G1 and C10 pair in the 1R4H reference structure not to be base paired, G1-C10 was considered a native base pair in the stem region, as reported by the Rijnbrand group that originally determined the structure by NMR.^54^ The fraction of native base pairs (*Q*) was evaluated for the stem regions. The *k*-means algorithm^85^ was used to sort the conformations sampled in three independent simulations into 20 clusters (that is, *k* = 20) for each model, where the RMSD for the entire molecule was used as a metric for comparing RNA structures. The clusters were numbered in order of decreasing population, i.e., the first cluster was the most populated, and the second cluster was the second most populated. For analyzing the folding of class II stem-loops, stems were divided into two regions: stem 1 was a duplex region formed between the hairpin loop and the bulge or internal loop, and stem 2 was a duplex region formed from the bulge or internal loop to the RNA terminal. Images of the molecular structures and simulation trajectory movies were created using ChimeraX.^86^

### Explicit Solvent MD Simulations for Stem-Loops

The initial structures were built from the first models of their NMR structures deposited in the PDB. Each system was solvated in TIP4P-D water boxes with 150 mM NaCl, maintaining a minimum distance of 15 Å between the solute and box edges. The DESRES-RNA^26^ and CHARMM22 ^87^ force fields were used for RNA and ions, respectively. The numbers of Na^+^ and Cl^–^ ions were calculated using SPLIT.^88^ Energy minimization was carried out in two stages: 1000 steps of the steepest descent, followed by 1000 steps of the conjugate gradient method. The systems were then heated linearly from 0 to 298 K over 500 ps at 1 bar, followed by 500 ps of equilibration at 298 K and 1 bar. A single 1 μs production simulation was conducted for each model. Long-range electrostatic interactions were calculated by the particle mesh Ewald method.^89^ Short-range electrostatic and Lennard-Jones interactions were calculated with a cutoff distance of 10 Å. Pressure was controlled using a Berendsen barostat with a relaxation time of 1 ps.^90^ All other simulation parameters were identical to those used in the GB-neck2 implicit solvent simulations.

### Implicit Solvent MD Simulations for rU_40_

MD simulations of rU_40_ single-stranded RNA (ssRNA) were performed three times, each for 1 μs, using the same protocol as that for stem-loops. The Förster resonance energy transfer (FRET)-averaged end-to-end distance ⟨*R*_FRET_⟩ of the rU_40_ was calculated from MD simulations according to the procedure described by Tan et al.^26^ The distance between the O5′ atom of U1 and the O3′ atom of U40, *R*, was converted into the FRET efficiency, *E*_FRET_, using the equation *E*_FRET_ = [1 + (*R*/*R*_0_)^6^]^−1^, where *R*_0_ was set to 55.0 Å according to the experimental results.^91^ The ⟨*R*_FRET_⟩ was then calculated using the equation ⟨*R*_FRET_⟩ = *R*_0_(⟨*E*_FRET_⟩^–1^ – 1)^1/6^, where ⟨*E*_FRET_⟩ is the ensemble-averaged value of *E*_FRET_. The standard error of the mean for ⟨*E*_FRET_⟩ was estimated by using three independent simulations.

### Implicit Solvent MD Simulations for RNA Duplex Formation

Three non-self-complementary RNA duplexes, CGCGG, ACUGUCA, and CGACGCAG, were simulated using the DESRES-RNA force field with the GB-neck2 implicit solvent model, starting from separate complementary strands. Initial structures were generated by using the *tleap* module of AMBER22, where the two complementary strands were positioned 30 Å apart along the *x*-axis. To prevent the two strands from diffusing away, a half-harmonic restraining potential was applied to the distance between the phosphorus atoms of the central residues in each strand. The ionic concentration was set to 1 M. Each simulation was conducted for 2 μs, and three independent runs were performed. All other simulation parameters were identical to those used in the stem-loop folding simulations. Full methodological details are provided in the Supporting Information.

## Results and Discussion

### Folding Trajectories of RNA Stem-Loops

The time evolutions of the fractions of native base pairs *Q* in stem regions from the 18 class I stem-loops shown in Figure 1A and the 8 class II stem-loops shown in Figure 1B are shown in Figures 2 and 3, respectively. The RMSD time series data for the stem and loop regions, *Q*, base pairs, and probability distributions of the RMSDs are presented in the Supporting Information (Figures S1–S26). Notably, all 18 class I stem-loops were able to fold into their conformations with *Q* = 1.0 in three independent 4 or 8 μs simulations starting from their extended conformations (Figure 2). The 10 nt molecules of 1R4H and 1IDV with three base pairs in their stem regions showed frequent folding and unfolding transitions during the simulation time. In the case of 2EVY, the folded state was unstable and lasted only 1 μs out of a 24 μs simulation time. Except for the above three RNA models, once the molecules folded into conformations with all of their native base pairs, their folded structures were stable until the end of the simulations. For class II stem-loops, five of the eight models (17RA, 2KF0, 1ANR, 1R2P, and 1N8X) were able to fold to the conformations with *Q* = 1.0 (Figure 3). In most cases, these RNA molecules reached the folded states with high *Q* values (*Q* > 0.6) and remained stable until the end of the simulations.

**Figure 2:**
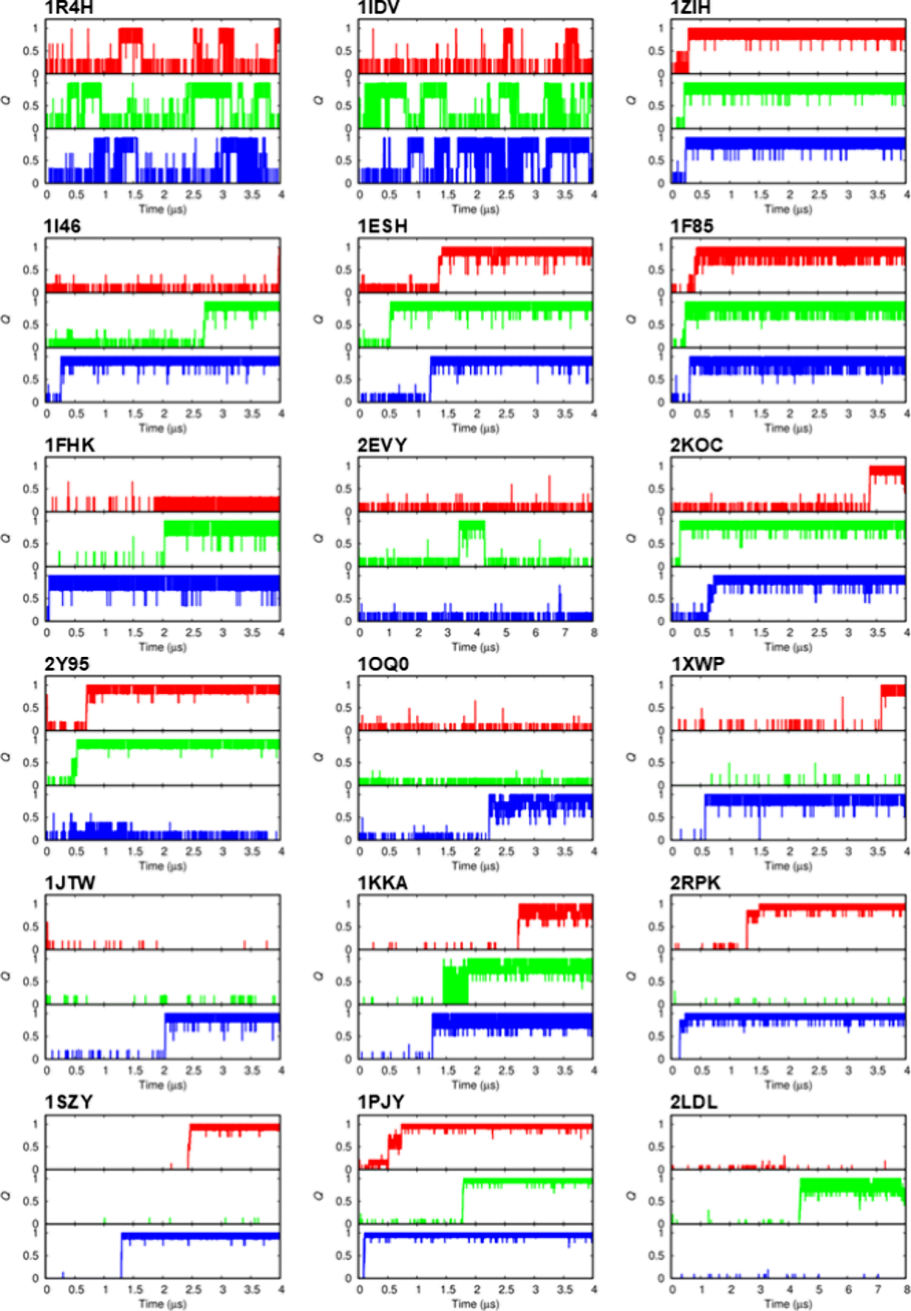
Time evolution of the fraction of native base pairs *Q* for 18 class I stem-loops without bulges or internal loops (Figure 1A). Three independent simulations (red, green, and blue lines) were performed for each model, starting from the extended conformations.

**Figure 3:**
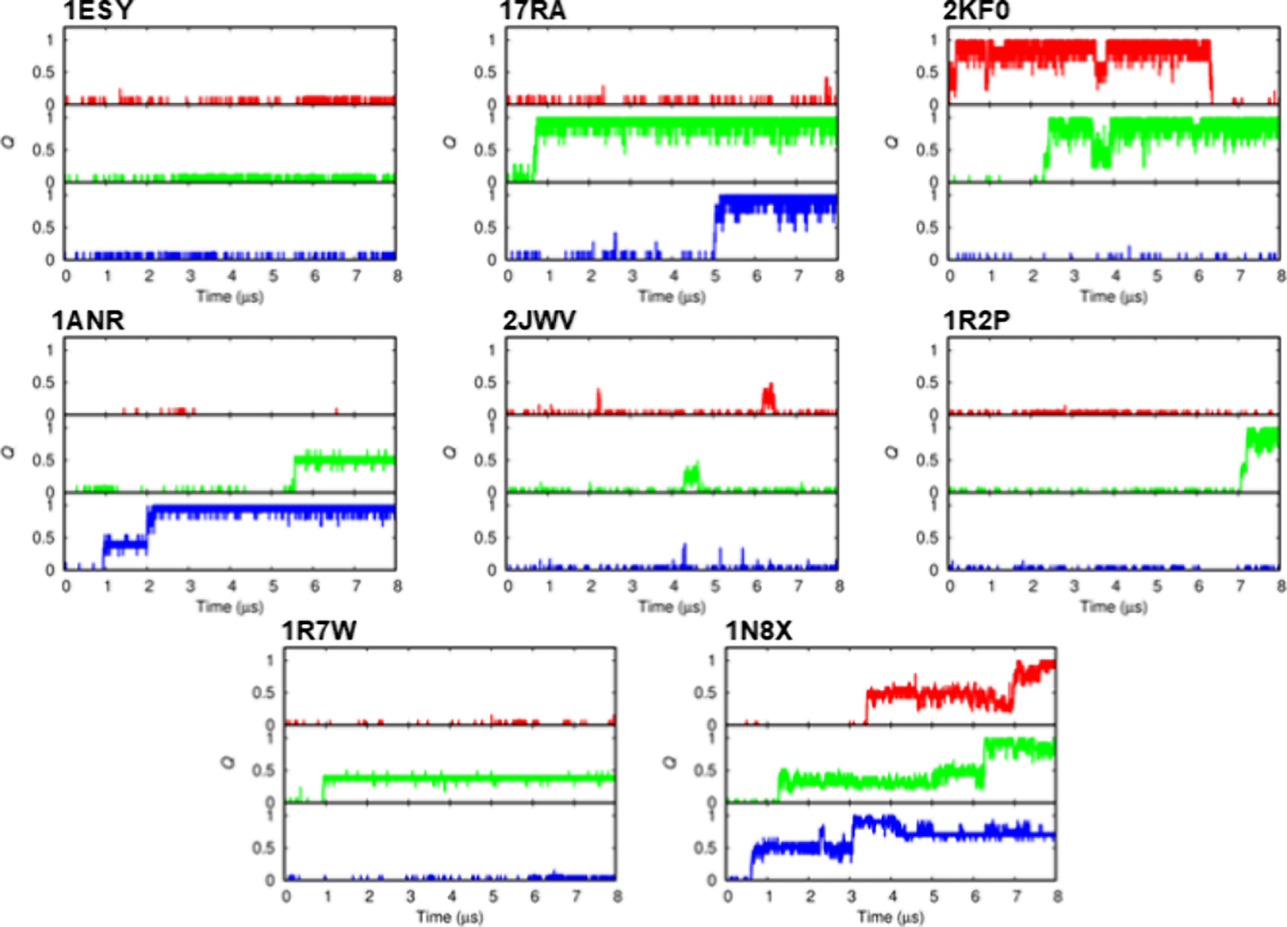
Time evolution of the fraction of native base pairs *Q* for eight class II stem-loops with bulges or internal loops (Figure 1B). Three independent simulations (red, green, and blue lines) were performed for each model, starting from the extended conformations.

### Cluster Analysis

Cluster analysis of the sampled conformations was performed using the *k*-means method to extract representative structures from the trajectories and to determine whether the folded structures formed a dominant cluster. In the cluster analysis, the overall RMSD was used to evaluate the structural differences between the two model conformations. The centroid structures for the class I stem-loops with the lowest RMSD values as well as the cluster numbers, RMSDs for the molecules, and *Q* values are presented in Figure 4. The fractions of the total trajectory, RMSDs for all, stem, and loop regions, and *Q* values for the top three clusters for the class I stem-loops are listed in Table 1. For 15 of the 18 class I stem-loops, the centroid structures with the lowest RMSD values among the 20 clusters belonged to their most populated first clusters (Figure 4). The centroid structures of the second and third most populated clusters for 1FHK and 1JTW, respectively, had the lowest RMSD values. The fractions of these clusters were 0.21 and 0.16 for 1FHK and 1JTW, respectively, which are still major components of their trajectories. For 2EVY, the centroid with the lowest RMSD value of 3.7 Å was in the 17th cluster due to the instability of the folded state of the model. The centroid structures with the lowest RMSD always have a *Q* of 1.0 and RMSD values for the molecules of <4.4 Å (Figure 4). In the stem region, the RMSD values of the centroids were <2.0 Å, except for 1I46 (2.2 Å) and 2LDL (3.0 Å) (Table 1). In contrast to the stem regions, the loop regions tended to have high RMSD values of approximately 4 Å (Table 1). This trend of inaccuracy in loop regions is clearly visible in Figure 4, where the loops were disrupted relative to their respective experimental structures despite the stem regions being nearly perfectly formed. Tan et al. reported folding simulations for 1ZIH and 2KOC with the DESRES-RNA force field and the TIP4P-D explicit solvent model using the simulated tempering technique, where the structures with stem and loop RMSDs <2 Å were substantially sampled at low temperatures.^26^ Thus, using the GB-neck2 implicit solvent model decreased the modeling accuracy of the loop region.

**Figure 4:**
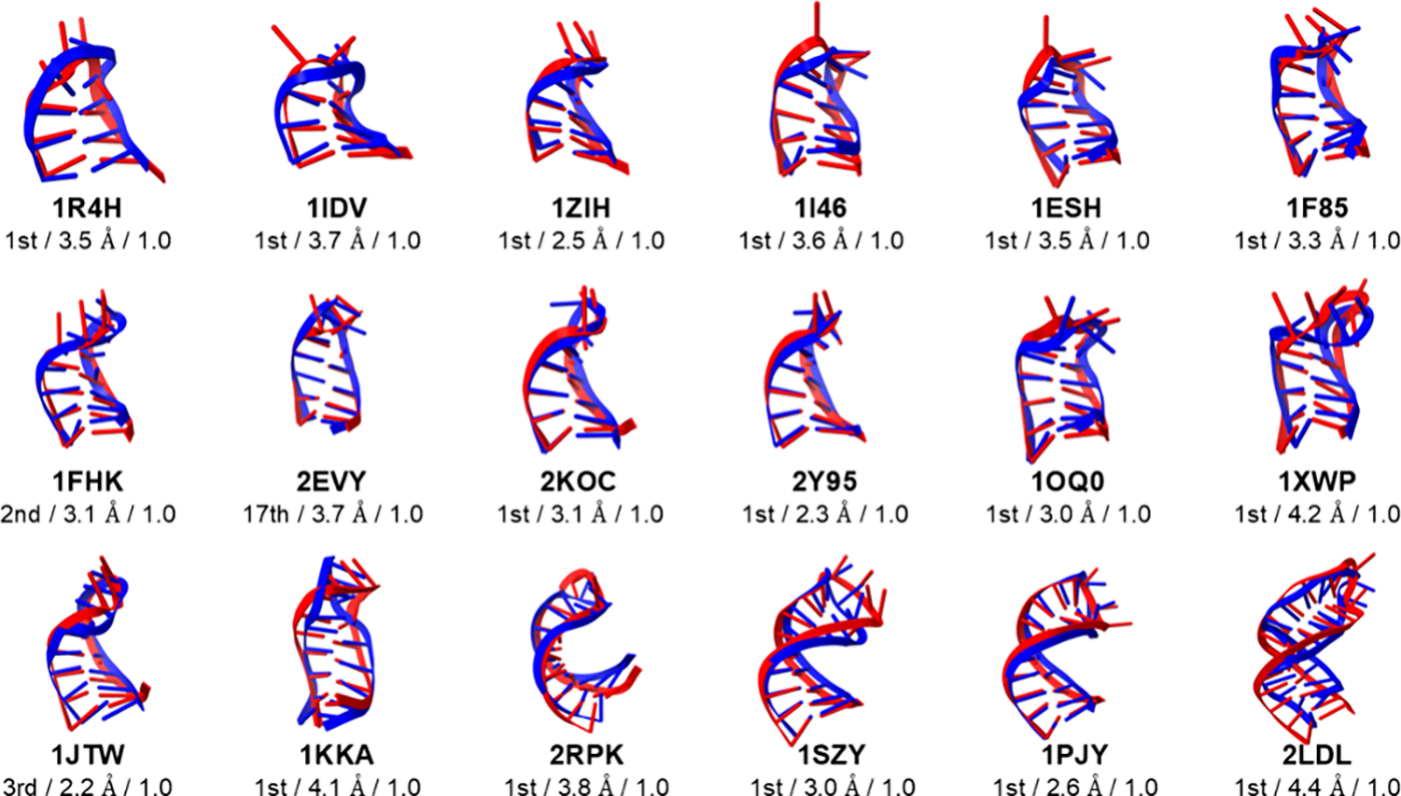
Comparison of class I stem-loop structures between the experiment (blue) and cluster centroid (red) exhibiting the lowest non-hydrogen root mean square deviation (RMSD) values for the entire molecule. Below the RNA name of each structure, the cluster number, RMSD value, and fraction of native base pairs (*Q*) are shown and separated by slashes. The *k*-means method with *k* = 20 was used to cluster the RNA structures sampled from the three independent molecular dynamics simulations starting from extended conformations.

**Table 1:** Fraction of the Total Trajectory, Non-Hydrogen Root Mean Square Deviation (RMSD; Å) for the Entire Molecule, Stem, and Loop Regions, and the Fraction of Native Base Pairs *Q* of the Centroids for the Three Most Populated Clusters of the 18 Class I Stem-Loops^a^

	First cluster					Second cluster					Third cluster				
		RMSD					RMSD					RMSD			
Model	Fraction	Overall	Stem	Loop	*Q*	Fraction	Overall	Stem	Loop	*Q*	Fraction	Overall	Stem	Loop	*Q*
1R4H	**0.16**	**3.5**	**1.8**	**4.5**	**1.0**	0.10	4.9	1.8	5.5	1.0	0.09	4.2	2.9	4.5	0.0
1IDV	**0.25**	**3.7**	**1.4**	**4.5**	**1.0**	0.16	3.7	2.9	3.8	0.3	0.05	8.9	9.8	2.8	0.0
1ZIH	**0.94**	**2.5**	**1.5**	**2.5**	**1.0**	0.02	7.0	6.6	5.7	0.0	0.01	11.7	13.6	4.3	0.0
1I46	**0.42**	**3.6**	**2.2**	**4.7**	**1.0**	0.07	6.9	6.5	4.1	0.0	0.05	13.7	14.7	4.8	0.0
1ESH	**0.74**	**3.5**	**2.0**	**4.5**	**1.0**	0.09	6.0	5.9	4.8	0.0	0.03	15.6	17.1	3.9	0.0
1F85	**0.75**	**3.3**	**1.3**	**4.8**	**1.0**	0.16	4.2	3.4	4.8	0.8	0.02	5.1	4.0	4.5	0.6
1FHK	0.28	4.4	1.1	5.3	1.0	**0.21**	**3.1**	**0.9**	**3.7**	**1.0**	0.12	8.4	5.1	7.2	0.0
2EVY ^b^	0.07	15.8	17.2	5.4	0.0	0.07	13.2	14.7	4.3	0.0	0.06	15.3	17.0	4.8	0.0
2KOC	**0.46**	**3.1**	**1.3**	**4.5**	**1.0**	0.18	4.2	1.5	6.5	1.0	0.03	14.5	15.1	4.3	0.0
2Y95	**0.56**	**2.3**	**1.2**	**3.6**	**1.0**	0.18	5.5	4.7	5.2	0.0	0.05	6.4	6.6	4.1	0.0
1OQ0	**0.24**	**3.0**	**1.9**	**4.2**	**1.0**	0.07	14.8	15.8	4.5	0.0	0.06	17.8	18.8	4.7	0.0
1XWP	**0.32**	**4.2**	**1.4**	**4.9**	**1.0**	0.06	18.8	20.0	8.3	0.0	0.05	18.0	19.4	6.7	0.0
1JTW	0.27	7.3	6.9	5.6	0.0	0.17	9.0	9.3	5.7	0.0	**0.16**	**2.2**	**1.4**	**2.1**	**1.0**
1KKA	**0.55**	**4.1**	**1.3**	**4.8**	**1.0**	0.05	20.0	22.5	5.3	0.0	0.05	16.2	17.9	6.0	0.0
2RPK	**0.55**	**3.8**	**1.1**	**4.4**	**1.0**	0.04	13.6	14.1	7.1	0.0	0.04	22.8	25.2	8.1	0.0
1SZY	**0.36**	**3.0**	**1.1**	**2.9**	**1.0**	0.05	21.6	24.0	7.2	0.0	0.04	20.3	22.1	7.3	0.0
1PJY	**0.83**	**2.6**	**1.8**	**3.9**	**1.0**	0.02	23.6	25.1	5.6	0.0	0.02	26.8	28.6	6.0	0.0
2LDL	**0.15**	**4.4**	**3.0**	**3.9**	**1.0**	0.07	28.9	32.1	6.0	0.0	0.06	28.3	31.2	8.0	0.0

a Cluster analysis was performed using the *k*-means method with *k* = 20. For each model, the cluster whose centroid structure had the lowest RMSD value among the 20 clusters is highlighted in bold.

b For 2EVY, the cluster with the lowest RMSD value was the 17th cluster, where the fraction of the cluster, the RMSD for the entire molecule, stem, and loop regions, and the *Q* value of the centroid were 0.04, 3.7 Å, 1.4 Å, 4.6 Å, and 1.0, respectively.

The centroid structures with the lowest RMSD values for the class II stem-loops, along with their cluster numbers, RMSD values for the molecules, and *Q* values are shown in Figure 5. The fraction of the trajectory, RMSDs for all, stem, and hairpin loop regions, and *Q* values for the top three clusters for the class II stem-loops are listed in Table 2. The RMSD values for the stems that directly connect to hairpin loops (stem 1) and those close to the 5′ and 3′ termini of the RNA (stem 2) are also listed. The conformations with *Q* = 1.0 were sampled for five class II stem-loops: 17RA, 2KF0, 1ANR, 1R2P, and 1N8X (Figure 5). Among the five folded models, the centroid structures of the most populated clusters for 17RA, 2KF0, 1ANR, and 1N8X gave the lowest RMSD values, with 17RA and 2KF0 at <3 Å. For 1ANR and 1N8X, the centroid structures had slightly higher RMSD values at 5.9 and 4.8 Å, despite *Q* values of 1.0 and 0.7, respectively. For 1R2P, the centroid structure of the 16th cluster had the lowest RMSD value of 8.3 Å with *Q* = 1.0. These results showed that the modeling accuracy of stem regions was high but that of hairpin and internal loop regions and the relative orientation between the two stem regions was low with the current DESRES-RNA force field in the GB-neck2 implicit solvent model.

**Figure 5:**
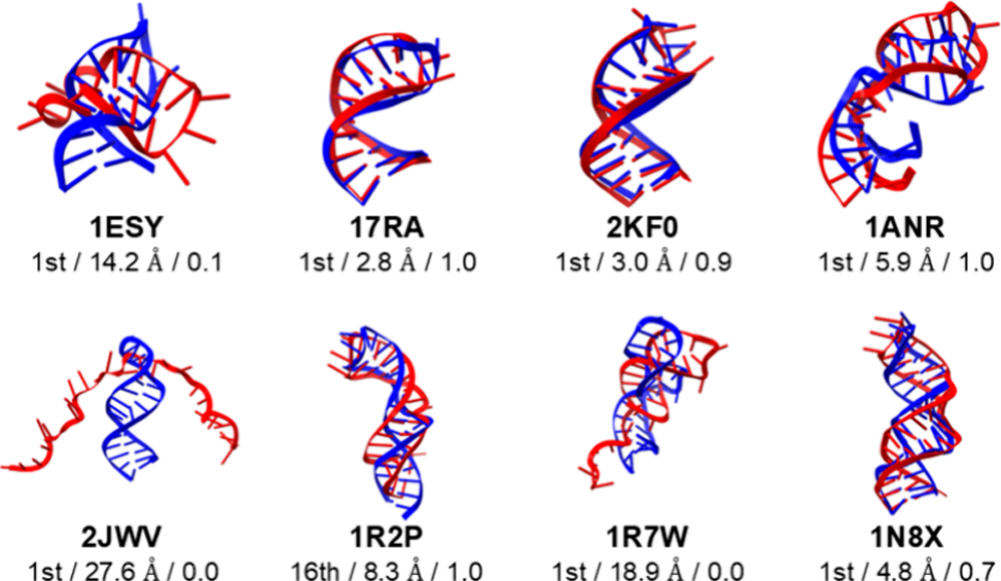
Comparison of the class II stem-loop structures with bulges or internal loops between the experiment (blue) and cluster centroid (red) exhibiting the lowest non-hydrogen root mean square deviation (RMSD) for the entire molecule. For models 1ESY, 2JWV, and 1R7W, which did not correctly fold, the centroid structure of the first cluster is shown. Below the RNA name of each structure, the cluster number, RMSD value, and fraction of native base pairs (*Q*) are shown and separated by slashes. The *k*-means method with *k* = 20 was used to cluster the RNA structures sampled from the three independent molecular dynamics simulations starting from extended conformations.

**Table 2:** Fraction of the Total Trajectory, Non-Hydrogen Root Mean Square Deviation (RMSD; Å) for the Entire Molecule, Stems 1 and 2, and Loop Regions, and the Fraction of Native Base Pairs *Q* of the Centroids for the Three Most Populated Clusters of the Eight Class II Stem-Loops^a^

	First cluster						Second cluster						Third cluster					
		RMSD						RMSD						RMSD				
Model	Fraction	Overall	Stem 1	Stem 2	Loop	*Q*	Fraction	Overall	Stem 1	Stem 2	Loop	*Q*	Fraction	Overall	Stem 1	Stem 2	Loop	*Q*
1ESY	0.23	14.2	9.5	7.5	6.2	0.1	0.10	9.1	6.6	5.8	5.4	0.0	0.08	11.0	8.7	9.4	6.1	0.0
17RA	**0.33**	**2.8**	**0.9**	**1.5**	**3.4**	**1.0**	0.11	4.2	0.7	1.6	3.9	1.0	0.05	21.4	9.4	24.1	4.1	0.0
2KF0	**0.47**	**3.0**	**1.0**	**0.7**	**4.7**	**0.9**	0.04	25.3	17.4	30.2	6.5	0.0	0.04	22.3	14.0	25.3	5.9	0.0
1ANR	**0.35**	**5.9**	**1.4**	**1.3**	**5.1**	**1.0**	0.10	22.6	16.2	25.2	7.1	0.0	0.08	25.0	18.3	25.5	8.1	0.0
2JWV	0.07	27.6	15.9	33.6	6.3	0.0	0.07	32.9	15.7	42.2	6.9	0.0	0.07	32.6	16.0	39.6	7.7	0.0
1R2P ^b^	0.09	32.7	17.0	38.3	6.2	0.0	0.07	37.1	16.7	46.0	5.5	0.0	0.07	29.5	15.9	33.7	4.3	0.0
1R7W	0.24	18.9	14.2	15.5	4.2	0.0	0.20	13.9	0.8	16.9	2.6	0.4	0.09	18.6	15.5	14.6	4.0	0.0
1N8X	**0.32**	**4.8**	**3.5**	**2.0**	**2.6**	**0.7**	0.16	5.9	1.8	6.4	2.1	0.5	0.13	9.7	3.9	10.6	3.2	0.4

a Cluster analysis was performed using the *k*-means method with *k* = 20. For each model, the cluster with a centroid structure that had the lowest RMSD value among the 20 clusters is highlighted in bold. For the models 1ESY, 2JWV, and 1R7W, the RNA did not fold into the structures with a *Q* value of 1.0; therefore, the clusters with the lowest RMSD values are not highlighted in bold in this table.

b For 1R2P, the cluster with the lowest RMSD value was the 16th cluster, where the fraction of the cluster, the RMSD for the entire molecule, stem 1, stem 2, and loop regions, and the *Q* value of the centroid were 0.04, 8.3 Å, 1.6 Å, 4.5 Å, 3.0 Å, and 1.0, respectively.

### Folding Kinetics

Conventional MD simulations were performed at 298 K to investigate the folding of various RNA stem-loops. One advantage of conventional MD simulations when studying biomolecular folding compared with enhanced sampling simulations, such as replica-exchange MD simulations, is the relatively easy analysis of folding/unfolding kinetics and mechanisms in a time-dependent manner.

Recent experimental studies have shown that the folding of simple stem-loops can occur on time scales ranging from microseconds to seconds.^31–36^ In the MD simulations using the Langevin thermostat with the implicit GB solvent model presented in this report, the folding of the class I stem-loops occurred on a time scale of several microseconds. The rate of conformational changes was increased using GB implicit solvent models relative to experiments that were approximately 10–100-fold with a collision frequency γ of 1 ps^–1^.^41^ Therefore, our simulations estimate folding times of tens to hundreds of microseconds for simple class I stem-loops, which are within the lower limit of the experimental estimate.

### Folding Mechanisms and Pathways

We show two representative trajectories of RNA stem-loops and discuss their folding mechanisms: 1SZY from the class I stem-loops and 1ANR from the class II stem-loops. The trajectories of the other stem-loops are shown in the Supporting Information (Figures S1–S26).

The time evolutions of the RMSD for the stem region and base pairs in the first trajectory of 1SZY in the class I stem-loops, as well as snapshots at selected time points, are shown in Figure 6. A simulation movie of this trajectory is provided in the Supporting Information (Movie S1). The RNA had seven base pairs in the stem region and a loop length of 7 nt. In the trajectory, folding occurred at approximately 2.4 μs from random conformations via a two-state transition without an intermediate state (Figure 6A). The stem-loop was zipped from the loop-closing base pair (G7-C15) to its terminal side of the chain (Figure 6A,C). In the trajectory, the loop-closing G7-C15 base pair was initially formed at 2.4314 μs from a random conformation, followed by the adjacent two base pairs G6-C16 and G5-C17 at 2.433 μs. The remaining stem base pairs formed by 2.4736 μs. Before folding at approximately 2.4 μs, the molecules formed almost no base pairs, and the RMSD for the molecule and stem region fluctuated between 10 and 30 Å, except at 1.3–1.9 μs, where the three misaligned base pairs (G1-U9, G2-C8, and C3-G7) were clearly formed. Misaligned base pairs were also observed in the second and third trajectories of the molecule (Figure S16).

**Figure 6:**
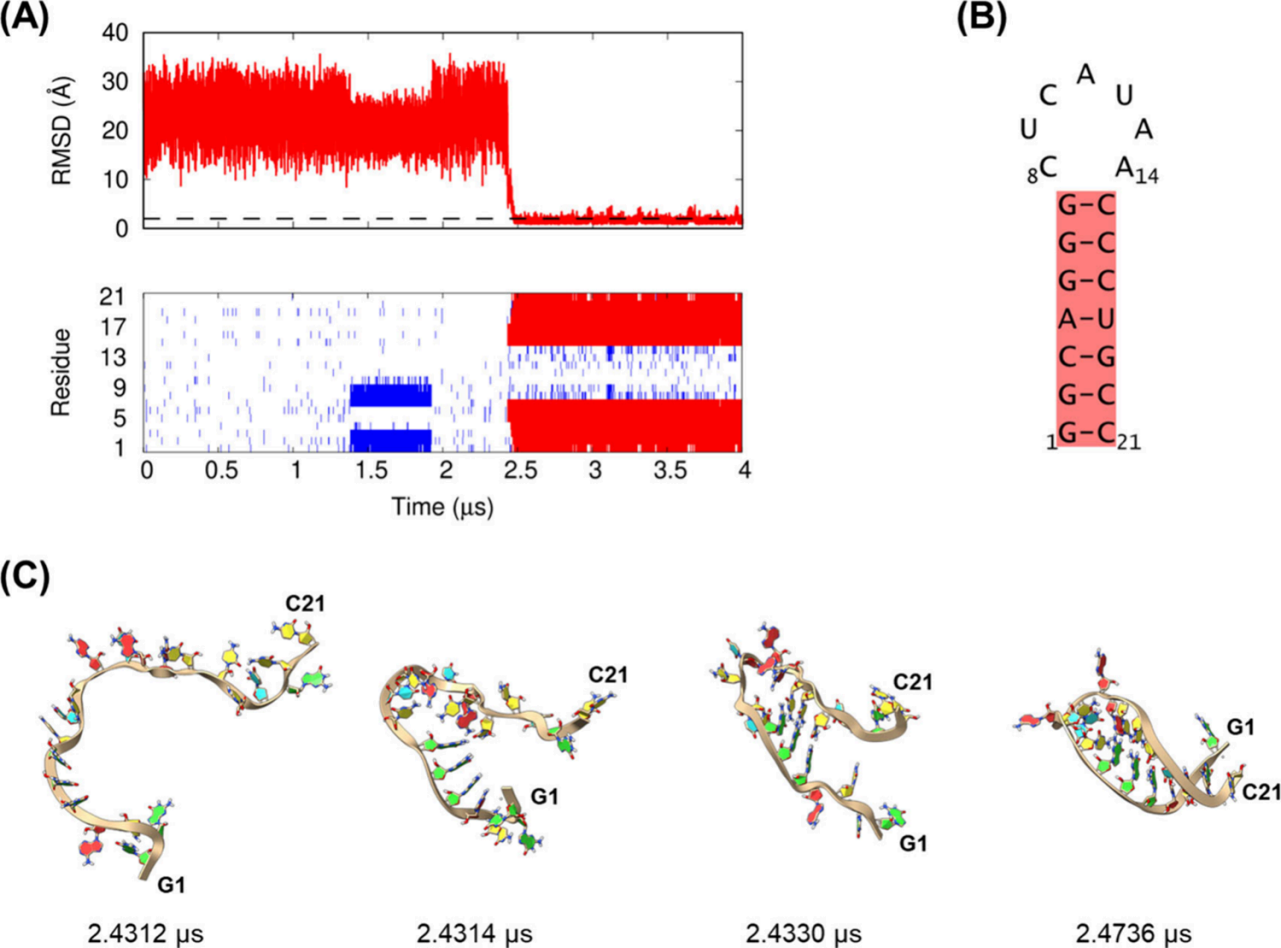
Folding trajectory of the 1SZY class I stem-loop from the first molecular dynamics simulation using the DESRES-RNA force field and the GB-neck2 model. (A) (Upper) Non-hydrogen root mean square deviation (RMSD) for the stem region. The dashed line indicates an RMSD of 2 Å. (Lower) Base pairs formed during simulation, where the native and non-native base pairs are represented by red and blue, respectively. (B) Secondary structure of 1SZY. The stem region is indicated in red. (C) Snapshots of the trajectory at various simulation time points. RNA backbones are represented by ribbons. The nucleotide ring structures are colored according to nucleotide type: adenine, red; cytosine, yellow; guanine, green; and uracil, cyan.

The time evolutions of the RMSD for the stem regions and base pairs in the third trajectory of 1ANR, as a representative trajectory for class II stem-loops, are shown in Figure 7. The figure also includes snapshots during the folding transition observed in this trajectory. A simulation movie of the trajectory is provided in the Supporting Information (Movie S2). The RNA consists of 29 nucleotides with an asymmetrical internal loop. Folding of the molecule from its extended conformations clearly occurred in a two-step manner: stem 1 was formed first, followed by stem 2, and they likely formed via the zipping mechanism from the hairpin loop-closing base pair for stem 1 and from the internal loop-closing base pair for stem 2. In the trajectory, four base pairs in the stem 1 region (C13-G20, G12-C21, A11-U22, and G10-C23) were initially formed by 0.998 μs from a random conformation (Figure 7C). This stem 1 duplex persisted for approximately 1 μs, whereas two strands in the stem 2 region largely fluctuated, corresponding to an intermediate state in the folding process. At 1.998 μs, the G5-C25 base pair in stem 2 and the A6-U24 base pair in the internal loop region of the molecule were formed, which promoted the formation of the remaining base pairs in the stem 2 region. The details of the A6-U24 base pair in the folded structures are described in the Supporting Information. All stem 2 base pairs were formed by 2.4 μs.

**Figure 7:**
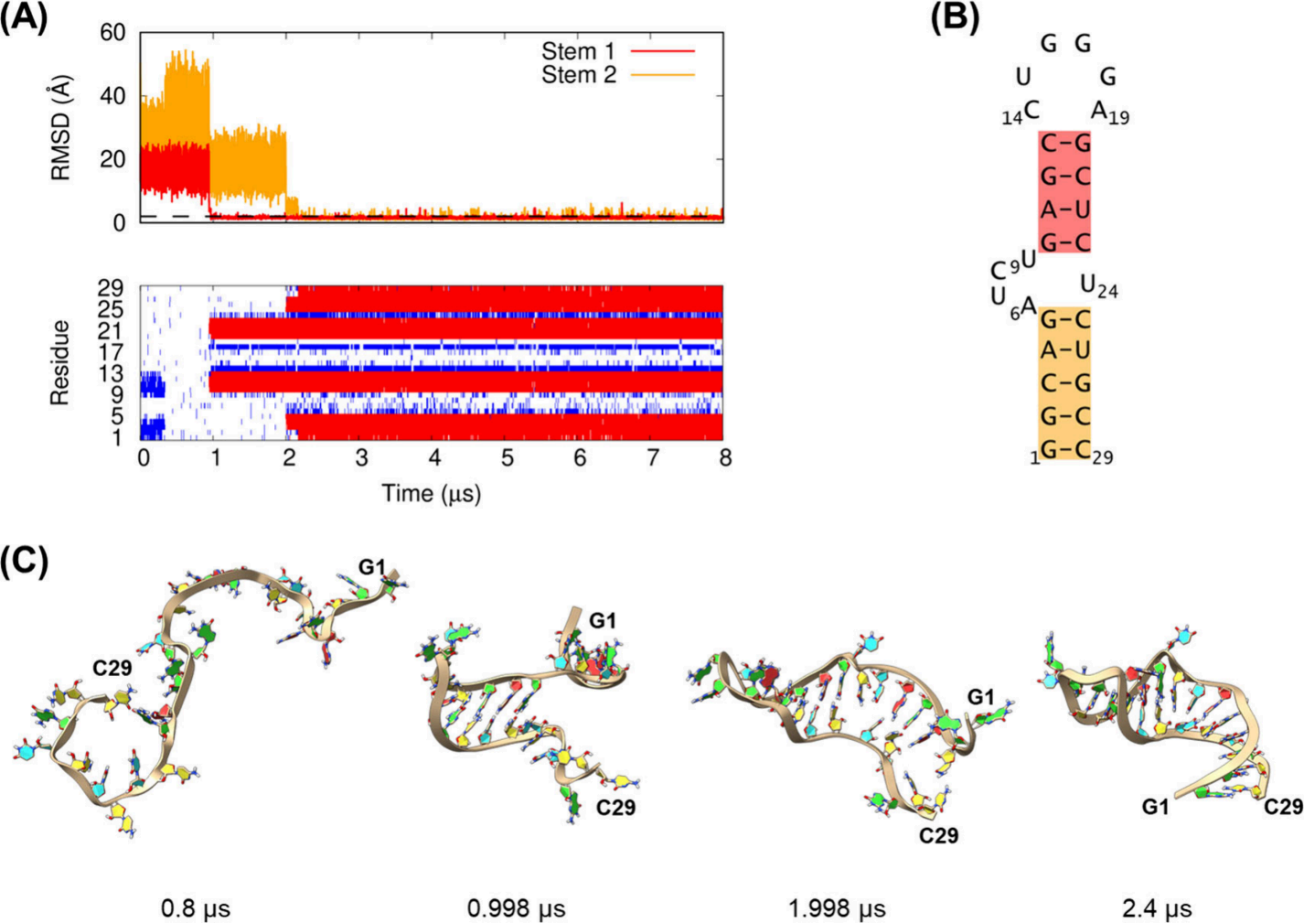
Folding trajectory of the 1ANR class II stem-loop from the third molecular dynamics simulation using the DESRES-RNA force field and the GB-neck2 model. (A) (Upper) Non-hydrogen root mean square deviation (RMSD) for the two stem regions, where stem 1 (red lines) and stem 2 (orange lines) represent the double helical regions directly connecting the hairpin loop and those near the 5′ and 3′ termini of the RNA, respectively. The dashed line indicates an RMSD of 2 Å. (Lower) Base pairs formed during simulations, where the native and non-native base pairs are represented in red and blue, respectively. (B) Secondary structure of 1ANR. Stems 1 and 2 regions are indicated in red and orange, respectively. (C) Snapshots of the trajectory at various simulation time points. RNA backbones are represented by ribbons. The nucleotide ring structures are colored according to nucleotide type: adenine, red; cytosine, yellow; guanine, green; and uracil, cyan.

The folding mechanisms of the two-state transition and zipping from the loop-closing base pairs observed in 1SZY were consistently reproduced across other class I stem-loops (Figures S1–S18). For class II stem-loops, the two-step folding pathway observed in 1ANR was essentially the same for the successful folding trajectories of 17RA, 2KF0, 1R2P, and 1N8X (Figures S20, S21, S24, and S26, respectively).

Three to four misaligned base pairs were frequently observed for other class I stem-loops. Without continuously misaligned base pairs, class I stem-loops displayed substantial molecular fluctuations, with RMSD values for the molecules and stem regions fluctuating rapidly over 10 Å, except for 1JTW. For 1JTW, the overall RMSD values remained stable at approximately 7 and 9 Å for most of the simulation time in the second and first trajectories, respectively (Figure S13a). The corresponding structures were found in the first (RMSD ≈ 7 Å) and second clusters (RMSD ≈ 9 Å), where the guanosine bases formed stable hydrogen bonds with the backbone phosphate and were simultaneously stacked with neighboring residues (Figure S27).

### Trajectories that Failed to Form Stable Native Stems: 2EVY, 1ESY, 2JWV, and 1R7W

The MD simulations for 2EVY, 1ESY, 2JWV, and 1R7W failed to form stable native stem-loop conformations. The MD simulations of the class I stem-loop 2EVY with the GB-neck2 model sampled structures with *Q* = 1.0; however, these structures were unstable and belonged to the 17th cluster (Figure 4 and Table 1). The centroid structures of the first and second clusters adopted expanded conformations (Figure S28). The stem of the molecule comprises five base pairs, beginning with a U-G wobble base pair at the loop-closing position followed by two A-U base pairs extending toward the terminal end. Given that other stem-loops tended to initiate base pair formation from the loop-closing pair and proceed toward the terminus via the zipping mechanism, stable base pairing near the loop region likely plays a crucial role in proper stem-loop folding. The current simulation model using DESRES-RNA and GB-neck2 may underestimate the stability of the G-U wobble and A-U pairs, which could limit the sampling of stably folded structures for 2EVY.

Class II stem-loop 2JWV could not fold into a structure with *Q* = 1.0 within the 3 × 8 μs MD simulations (Figure S23). While transient formation of the stem 1 region was observed, stable base pairs, including non-native base pairs, did not form during the simulations (Figure S23). The centroid structure of the first cluster adopted an expanded conformation (Figure 5), and its population fraction was only 0.07, which is lower than the values of other stem-loop models. Stem 1 consists of four base pairs: a loop-closing U-G wobble pair followed by one C-G and two A-U base pairs to the internal loop. Because this region contains only a single G-C pair, like 2EVY, the current simulation model failed to form a stable duplex in stem 1, resulting in unsuccessful global folding of the molecule.

Class II stem-loops 1ESY and 1R7W were also unable to fold into a structure with *Q* = 1.0 within the 3 × 8 μs MD simulations (Figures S19 and S25, respectively). In the unsuccessful folding trajectories of 1ESY and 1R7W, misaligned base pairs were stably formed with reduced RMSD fluctuations. These misfolded structures formed dominant clusters, which are shown in Figure S29 along with their secondary structure diagrams. The persistence of the misfolded states likely prevents the successful folding of both RNA molecules.

These cases underscore the need for further refinement of the force field parameters, longer simulation durations, and the integration of enhanced sampling techniques to achieve more reliable and accurate RNA folding in MD simulations.

### Comparison of Loop and Bulge Structures with Explicit Solvent Simulations and NMR Data

We investigated the potential causes of the loop and bulge structure modeling inaccuracies observed with the DESRES-RNA force field and the GB-neck2 implicit solvent model. Residues directly involved in binding to proteins, nucleic acids, and small metabolite molecules are predominantly found in single-stranded loop regions.^92^ Enhancing the modeling accuracy of these loop and bulge regions is crucial for advancing the use of MD simulations in RNA biology. Here, using four representative stem-loops, 1F85, 1FHK, 2KOC (class I), and 1R2P (class II), the sampled loop and bulge structures from the MD simulations using the DESRES-RNA force field with the GB-neck2 implicit solvent model were compared with those obtained from 1 μs MD simulations using the same RNA force field combined with the TIP4P-D explicit solvent model. These RNA molecules were chosen based on their distinct structural features. The analysis aimed to determine whether the observed inaccuracies could be primarily attributable to the RNA force field or the implicit solvent model.

Trajectories of the RMSDs for these stem-loops in the explicit solvent MD simulations are shown in Figure S30. The stem regions of the three selected class I stem-loops remained stable, with RMSD values consistently <2.0 Å throughout the 1 μs explicit solvent simulations. For 2KOC, the loop conformation remained stable, with an RMSD < 1.5 Å for most of the simulation time, averaging 0.7 Å. In contrast, the loop regions of 1F85 and 1FHK exhibited a marked increase in RMSD to approximately 3.5 Å at around 0.5 μs, which persisted until the end of the simulations. For class II stem-loop 1R2P, the overall RMSD increased to approximately 8.0 Å (see Figure S33 for the resulting overall structure). Elevated RMSD values were observed in the terminal stem 2 (∼4.5 Å) and bulge regions (∼3.3 Å), whereas the stem 1 and loop regions remained stably formed, with RMSD values consistently below 2.0 Å.

The loop structures of four stem-loops derived from reference NMR models, explicit solvent MD snapshots at 1 μs, and cluster centroids with the lowest overall RMSD values in the GB-neck2 implicit solvent MD are shown in Figure 8. For 1F85, the NMR structure features a sheared G6-A9 base pair, a critical interaction governing the loop conformation of the molecule.^58^ This noncanonical pairing was found in the structure of the explicit solvent MD simulation. However, U8 adopted a different orientation relative to the NMR structure, contributing to the elevated RMSD values in the loop region. In the structure of the GB-neck2 implicit solvent MD simulations, the noncanonical sheared base pair G6-A9 was completely disrupted, and A9 was pointed toward the outside of the loop. In the first cluster of the implicit solvent simulations, the fraction of structures forming the sheared G6-A9 base pair was less than 1%.

**Figure 8:**
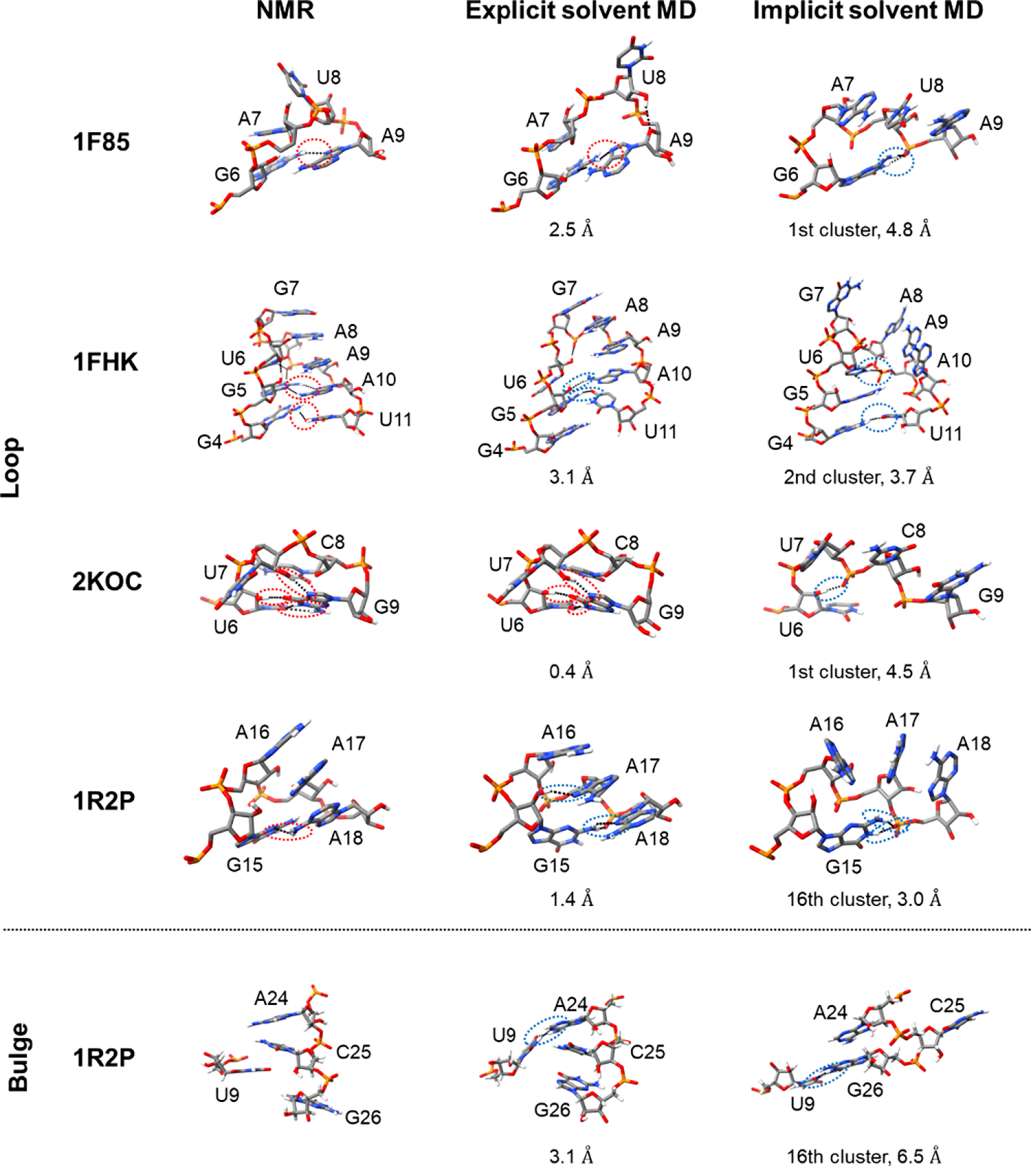
Comparison of the loop and bulge structures in four representative RNA stem-loops from NMR and molecular dynamics (MD) simulations with explicit and implicit solvent models. Loop regions of 1F85, 1FHK, 2KOC, and 1R2P, as well as the bulge region of 1R2P, are shown for three structural sources: NMR-derived structures (left column), simulations using the DESRES-RNA force field with the TIP4P-D explicit solvent model (middle column), and simulations using the same force field with the GB-neck2 implicit solvent model (right column). Dashed black lines indicate hydrogen bonds. In the NMR structures, key hydrogen bonds stabilizing the loop conformations are highlighted with red dashed circles. For the TIP4P-D explicit solvent MD simulations, snapshots at 1 μs are shown with non-hydrogen atom root mean square deviation (RMSD) values for the loop and bulge regions. For the GB-neck2 implicit solvent MD simulations, the centroid structures with the lowest RMSD values for the entire molecule are shown with their cluster numbers and RMSD values for the loop and bulge regions. In the structures from the explicit and implicit solvent MD simulations, hydrogen bonds consistent with those observed in the NMR structures are highlighted with red dashed circles. In contrast, hydrogen bonds that are formed exclusively in the simulated structures are highlighted with blue dashed circles.

For 1FHK, the NMR structure has sheared G4-U11 and G5-A10 mismatch base pairs and a contiguous base stacking from G7 to U11.^59^ The reported absence of a nuclear Overhauser effect (NOE) between the imino protons of G4 and U11 in NMR experiments suggests that G4 and U11 do not form a wobble base pair.^59^ In the explicit solvent MD simulation, although the contiguous base stacking from G7 to U11 was preserved, the two sheared base pairs, G4-U11 and G5-A10, were not formed. In the implicit solvent MD simulations using the GB-neck2, G4-U11 adopted a wobble base pair, and A10 pointed outward from the loop without pairing with G5. The contiguous base stacking pattern was also disrupted. In the second cluster of the implicit solvent simulations, whose centroid structure exhibited the lowest RMSD, less than 1% of the structures formed the sheared G4-U11 and G5-A10 mismatch base pairs. In contrast, G4 and U11 formed wobble base pairs in 87% of the structures within the cluster.

For 2KOC, the NMR structure features a *trans*-wobble base pair, U6-G9, which includes an unusual 2′-OH hydrogen bond.^61^ In addition, a hydrogen bond between the 2′-OH of U7 and the N7 of G9 is also formed. In the structure from the explicit solvent MD simulation, these key interactions between U6 and G9, as well as between U7 and G9, were stably maintained, contributing to the low RMSD values for the loop region. In contrast, the implicit solvent MD simulation led to the disruption of these interactions, with the C8 and G9 bases adopting orientations that diverged from those in the NMR structure. In the first cluster of the implicit solvent simulations, the *trans*-wobble U6-G9 base pair was formed in less than 1% of the structures within the cluster.

For the 1R2P class II stem-loop, chemical shift and NOE data from NMR experiments indicate that the GAAA tetraloop (G15-A18) adopts a stable conformation, with A16 tightly stacked.^76^ In the NMR structure, G15 and A18 are in close proximity, forming a partial hydrogen bond between G15 (N3) and A18 (N6-H). This conformation was preserved in the explicit solvent MD simulation, yielding a low loop RMSD of 1.4 Å. Although the G15-A18 hydrogen bond observed in the NMR structure was disrupted, three new hydrogen bonds were formed: two involving G15 (N2-H_2_), one with A18 (N3) and another with its backbone phosphate oxygen atom, and a third between G15 (2′-OH) and A17 (N7), potentially contributing to loop rigidity. In contrast, the implicit solvent MD simulation showed weakened stacking of A16 with A17 and an outward orientation of A18. The nucleobase hydrogen bond between G15 and A18 observed in the NMR structure was lost and replaced by hydrogen bonds between G15 (N1-H/N2-H) and a backbone phosphate oxygen of A18, resulting in an elevated loop RMSD of 3.0 Å.

The structures of the bulge region in the 1R2P class II stem-loop obtained from the explicit and implicit solvent MD simulations are also shown in Figure 8. In the bulge region of 1R2P, comprising residues U9, A24, C25, and G26, the U9 residue can, in principle, form either a Watson–Crick base pair with A24 or a wobble base pair with G26. However, NMR experiments revealed no evidence of an interaction between U9 and A24 or between U9 and G26.^76^ The G26 residue adopted a *syn* conformation and flipped into the major groove of duplex stem 2, precluding canonical base pairing.^76^ In the explicit solvent MD simulation, the bulge region exhibited a slightly elevated average RMSD of 3.3 Å. The G26 residue consistently adopted the *syn* conformation throughout the trajectory. Over the 1 μs simulation, U9-A24 and U9-G26 base pairs were observed in 55% and 20% of the sampled structures, respectively. The U9-A24 pairing frequency in the explicit solvent MD simulation may be overestimated relative to experimental observations. In contrast, the GB-neck2 implicit solvent MD simulation showed markedly different behavior: only 5% of the structures in the 16th cluster adopted the *syn* conformation, and 81% formed a U9-G26 base pair. The RMSD of the bulge region in the centroid structure reached 6.5 Å, which is substantially higher than that observed in the explicit solvent simulation. These structural features observed in the GB-neck2 implicit solvent MD simulation are clearly inconsistent with experiments, suggesting limitations of the implicit solvent model in accurately capturing native structural preferences within the bulge region.

MD simulations using the DESRES-RNA force field with the TIP4P-D explicit solvent model partially reproduced key hydrogen bonds and base stacking, stabilizing distinct loop conformations for four representative stem-loops. Conversely, MD simulations using the same RNA force field with the GB-neck2 implicit solvent model completely disrupted these key interactions in the loop regions. Although only a single bulge model was examined in this study, the bulge conformation generated by the explicit solvent MD simulation more closely resembled the NMR structure in comparison to that obtained from the implicit solvent MD simulation. While longer simulations and application of enhanced sampling techniques across a broader set of RNA stem-loop structures are needed in explicit solvent simulations for a more rigorous comparison, the results presented here indicate that the implicit solvent representation substantially reduces modeling accuracy in the loop and bulge regions of the RNA stem-loops.

### Comparison of Noncanonical and Transient Base Pairing with NMR Data

Several of the RNA stem-loops examined in this study include regions with potential base pairing that are not detected experimentally or whose base pairing behavior is modulated by pH conditions or ligand binding. We analyzed these base pairing patterns in the MD simulations using the DESRES-RNA force field and the GB-neck2 implicit solvent model and compared them with NMR data, which are described in the Supporting Information. Since NMR data reflect ensemble-averaged conformations in solution, whereas MD simulations yield time-resolved trajectories, direct comparison remains nontrivial due to differences in temporal resolution and conformational averaging. Nevertheless, many of the simulated base pairing patterns appeared to deviate from those observed in the NMR experiments. These findings, together with the results described above, suggest that further refinement of both the force field and the implicit solvent model is necessary to improve the accuracy of the RNA loop modeling.

### Flexibility of the ssRNA rU_40_

To further investigate potential causes of loop structure modeling inaccuracies observed with the DESRES-RNA force field and GB-neck2 implicit solvent model, the flexibility of a single-stranded RNA was evaluated from MD simulations. Single-stranded loop regions generally exhibit greater flexibility than duplex stem regions. Consequently, the intrinsic flexibility of RNA models for a given force field in MD simulations can significantly affect the results of loop structure modeling. We evaluated the FRET-averaged end-to-end distance ⟨*R*_FRET_⟩ of rU_40_ from the implicit solvent MD simulation with the DESRES-RNA and AMBER-OL3 RNA force fields for comparison (see Models and Simulation Methods for details of ⟨*R*_FRET_⟩). Lower ⟨*R*_FRET_⟩ values indicate higher flexibility in the RNA model. The ⟨*R*_FRET_⟩ values of rU_40_ at various ionic concentrations were evaluated by experiments^91^ and MD simulations using the DESRES-RNA and AMBER-OL3 force fields with explicit water models.^26^

The ⟨*R*_FRET_⟩ values calculated from the GB-neck2 implicit solvent and explicit solvent MD simulations and experiments at various ionic concentrations are shown in Figure 9. The end-to-end distance trajectories in the MD simulations with GB-neck2 at a 0.15 M ionic concentration are provided in the Supporting Information (Figure S31). The ⟨*R*_FRET_⟩ values calculated from the simulations with the DESRES-RNA force field and GB-neck2 were consistently higher than the experimental values across all ionic concentrations examined in this study, particularly at lower ionic concentrations. At 0.2 M ionic concentration, for example, the ⟨*R*_FRET_⟩ value for the DESRES-RNA with GB-neck2 was 82 Å, which is 1.3 times higher than the experimental value of 64 Å. At 1.0 M ionic concentration in the GB-neck2 implicit solvent, the simulation with the DESRES-RNA yielded a ⟨*R*_FRET_⟩ value of 70 Å, which is close to the experimental value of 68 Å observed at 0.05 M NaCl. Similarly, the AMBER-OL3 force field combined with GB-neck2 also yielded higher ⟨*R*_FRET_⟩ values than the experimental results at ionic concentrations of 0.15 and 0.2 M. These values were slightly lower than those of the DESRES-RNA with GB-neck2. In contrast, the ⟨*R*_FRET_⟩ values obtained using explicit solvent models were significantly lower than the experimental values. For the DESRES-RNA force field with the TIP4P-D water model, the ⟨*R*_FRET_⟩ value at 0.05 M NaCl was close to the experimental value, whereas the value at 0.2 M NaCl was 41 Å, 0.64 times the experimental result. For the AMBER-OL3 force field with the TIP3P water model, the ⟨*R*_FRET_⟩ value at 0.2 M NaCl was 24 Å, corresponding to 0.38 times the experimental value. These results indicate that the flexibility of the RNA model is highly sensitive to solvent models, with values observed in the implicit solvent model being significantly higher than those in explicit solvent models.

**Figure 9:**
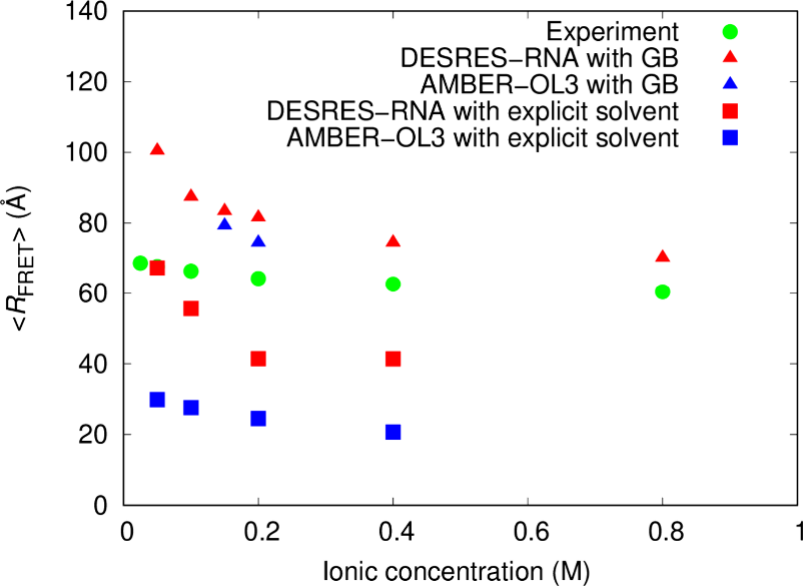
FRET-averaged end-to-end distances ⟨*R*_FRET_⟩ (Å) of rU_40_ single-stranded RNA obtained from molecular dynamics (MD) simulations using the DESRES-RNA and AMBER-OL3 force fields with the GB-neck2 implicit and explicit solvent models at various ionic concentrations. Standard errors for the simulation data obtained using the GB-neck2 model are too small to be visible in the plot. Values for explicit solvent simulations were obtained from the plot in ref 26, where the TIP4P-D and TIP3P water models were used with the DESRES-RNA and AMBER-OL3 force fields, respectively, for the MD simulations of rU_40_ at 300 K. The experimental values were obtained from the plot reported in ref 91. In both the experiment and the explicit solvent MD simulations, NaCl was used as the background electrolyte.

Tan et al. reported that the single-stranded loop regions of the tetraloops, 2KOC and 1ZIH, repeatedly adopted conformations that were <0.8 Å RMSD from the experimental structures in the simulated tempering MD simulations at 0.15 M NaCl using the DESRES-RNA force field and the TIP4P-D explicit water model.^26^ In our MD simulations using the DESRES-RNA force field and the GB-neck2 implicit solvent model, the loop region of 1ZIH frequently adopted conformations with an RMSD of less than 1.0 Å (Figure S3). In contrast, the loop region of 2KOC did not largely adopt the conformations with RMSD < 1.0 Å in the MD simulations using GB-neck2; instead, the RMSD values fluctuated around 5 Å for the conformations with *Q* > 0.8, which were almost indistinguishable from those with *Q* < 0.2 (Figure S9). The combination of DESRES-RNA and the TIP4P-D model showed RNA strand flexibility higher than that of the experiment, but the modeling accuracy of the loop region was higher than that of the combination of DESRES-RNA and the GB-neck2 model. Therefore, adjusting force field parameters to enhance the ssRNA flexibility in the implicit solvent model may improve the accuracy of loop region modeling.

### Toward More Accurate RNA Stem-Loop Folding Simulations

This section highlights several factors that require refinement to improve the accuracy of RNA modeling. As discussed above, RNA structural dynamics in MD simulations using the DESRES-RNA force field are highly sensitive to the choice of the solvent model. In simulations with the GB-neck2 implicit solvent model, hydrogen bonding between bases in loop regions tends to be disrupted, and RNA molecules exhibit reduced flexibility. In contrast, simulations employing the TIP4P-D explicit solvent demonstrate partial preservation of base–base hydrogen bonding within loop regions and greater conformational flexibility. Furthermore, analyses of 2EVY and 2JWV suggest that the current model may underestimate the stability of G-U wobble base pairs and canonical A-U base pairs. It is worth noting that the GB-neck2 parameters for nucleic acids were optimized against Poisson–Boltzmann calculations using a training set composed exclusively of stable DNA and RNA duplex conformations formed by canonical Watson–Crick base pairing. This may contribute to deficiencies in modeling loop region interactions and in accurately capturing the folding behavior of diverse RNA sequences. Since loop structures are often stabilized by noncanonical base pairs with diverse conformations, expanding the GB-neck2 training set to include a broader range of conformations, such as single-stranded RNA, loop structures, and noncanonical base pairs, is essential for improving its accuracy and generalizability in modeling complex RNA structures beyond regular duplexes.

In the simulations conducted in this study, the nonpolar contribution to the solvation free energy was not considered. Linzer et al. approximated the nonpolar solvation effect by strengthening Lennard-Jones interactions between pairs of heavy atoms in bases, which improved loop region modeling accuracy with the AMBER-OL3 RNA force field and GB-neck2 implicit solvent model.^52^ Therefore, incorporating the nonpolar solvation term in energy calculations may also improve the performance of the DESRES-RNA force field with the GB-neck2 implicit solvent model, particularly in accurately capturing loop region conformations.

Finally, it is worth mentioning the necessity of incorporating the effects of divalent cations in MD simulations for RNA systems to accurately capture biologically relevant conformations and interactions. Divalent cations, like Mg^2+^, play critical roles in RNA structures, folding, and functions.^50,93–97^ The ionic strength dependence of the persistence length of rU_40_ varies markedly between the presence of NaCl and MgCl_2_, with Mg^2+^ significantly increasing the rigidity of the ssRNA even at the same ionic strength.^91^ To assess the influence of Mg^2+^ ions on the RNA structure, explicit solvent MD simulations of the 1R2P stem-loop were performed in the presence and absence of Mg^2+^ ions. The simulation protocol and results are provided in the Supporting Information (Figures S32 and S33). The results underscore both the importance and the inherent challenges of accurately modeling Mg^2+^ ion interactions in MD simulations. In the current standard GB implicit solvent models, including the GB-neck2 model, the effects of monovalent ions are introduced using a mean-field approximation (the linearized Debye–Hückel approximation), whereas multivalent ions remain out of reach. Hybrid explicit ions/implicit solvent models that use the GB calculation framework^98^ may help tackle this problem.

## Conclusions

Atomic-level computer simulations of RNA systems are crucial for understanding RNA structure–dynamics–function relationships. RNA molecule folding simulations provide stringent tests that can be used to determine whether current molecular mechanics force fields and simulation methodologies can accurately characterize large conformational changes in RNA from long-time-scale MD simulations. In this study, we report that the DESRES-RNA force field combined with the GB-neck2 implicit solvent model can successfully simulate the folding of stem regions in 23 RNA stem-loops with varying sequences and lengths, including those with bulges, internal loops, and noncanonical wobble base pairs. The combination of the DESRES-RNA force field and the GB-neck2 implicit solvent model also successfully reproduced RNA duplex formation of CGCGG, ACUGUCA, and CGACGCAG, starting from two separate complementary strands, with details of the simulation protocols and results included in the Supporting Information (Figures S34–S36 and Table S1). Accurate modeling of loop structures remains challenging in simulations using the implicit solvent model. Conventional MD simulations were performed at room temperature. The enhanced sampling MD simulations with the DESRES-RNA force field and GB-neck2 provide a free energy landscape for RNA folding, offering valuable insights into folding mechanisms and potentially facilitating further optimization of RNA force field parameters. In MD simulations employing the DESRES-RNA force field and the GB-neck2 implicit solvent model, improving the conformational flexibility of single-stranded RNAs and the reproducibility of hydrogen bonds in loop regions is expected to increase the predictive accuracy of stem-loop folding and loop conformation modeling. This improvement could be achieved through optimization of GB parameters using a structurally diverse set of RNA molecules and by incorporating a nonpolar term in solvation free energy calculations. Accurate treatment of the interactions between RNA and divalent cations is also necessary for further advancements. The ability to recapitulate RNA stem folding of fundamental stem-loop motifs, which is reported in this study, represents a significant milestone toward accurately modeling RNA tertiary structures using MD simulations.

## Supplementary Material









## Data Availability

The input files used for MD simulations with AMBER22 are described in the Supporting Information. All data will be made available upon request.

## Notes

Time evolutions of RMSD, fraction of native base pairs *Q*, formed base pairs, and probability distributions of RMSD values for class I and class II stem-loops; centroid structures of the first and second clusters of 1JTW and 2EVY; misfolded structures and corresponding secondary structure diagrams forming the first clusters for 1ESY and 1R7W; trajectories of 1 μs MD simulations for three class I stem-loops and one class II stem-loop; comparison of noncanonical and transient base pairing with NMR data; trajectories of the rU_40_ ssRNA end-to-end distance; explicit water molecular dynamics simulation of an RNA stem-loop in the presence of divalent magnesium ions; trajectories of 1 μs MD simulations for 1R2P in the absence and presence of magnesium ions; comparison of overall, loop, and bulge structures of 1R2P; MD simulations of RNA duplex formation); trajectories of three independent molecular dynamics simulations of CGCGG, ACUGUCA, and CGACGCAG RNA duplex formation; non-hydrogen atom RMSD, base pairing, and C3′-endo sugar pucker presence evaluated over the last 0.5 μs of the RNA duplex model simulations; descriptions of simulation movies; and input files used for AMBER MD simulations (PDF)
Movie S1: Simulation movie of a 1SZY class I stem-loop folding observed in the first molecular dynamics simulation using the DESRES-RNA force field and the GB-neck2 implicit solvent model (MP4)
Movie S2: Simulation movie of a 1ANR class II stem-loop folding observed in the third molecular dynamics simulation using the DESRES-RNA force field and the GB-neck2 implicit solvent model (MP4)
Movie S3: Simulation movie of a CGACGCAG RNA duplex formation in the first molecular dynamics simulation using the DESRES-RNA force field and the GB-neck2 implicit solvent model (MP4)
