# Supplementary material for: Molecular Dynamics Simulations of RNA Stem-Loop Folding Using an Atomistic Force Field and a Generalized Born Implicit Solvent
Source: ACS Omega. 2025 Oct 26;10(43):51011–27. doi: 10.1021/acsomega.5c05377 (PMC12593105; doi:10.1021/acsomega.5c05377)
Supplement: Supplementary file 1 [file ao5c05377_si_001.pdf]

SUPPORTING INFORMATION

Molecular Dynamics Simulations of RNA Stem-Loop Folding Using an Atomistic Force Field and a Generalized Born Implicit Solvent

Tadashi Ando [a,b*]

[a] Department of Applied Electronics, Tokyo University of Science, 6-3-1 Niijuku, Katsushika-ku, Tokyo 125-8585, Japan

[b] Research Institute for Science and Technology, Tokyo University of Science, 2641 Yamazaki, Noda, Chiba 278-8510, Japan

* E-mail: tando@rs.tus.ac.jp

Table of Contents

1. Time evolutions of root mean square deviation (RMSD), fraction of native base pairs *Q*, formed base pairs, and probability distributions of RMSD values for class I stem-loops (Figures S1–S18) ··········· p. S3–S20
2. Time evolutions of root mean square deviation (RMSD), fraction of native base pairs *Q*, formed base pairs, and probability distributions of RMSD values for class II stem-loops (Figures S19–S26) ··········· p. S21–S28
3. Centroid structures of the first and second clusters of 1TJW (Figure S27) ··········· p. S29
4. Centroid structures of the first and second clusters of 2EVY (Figure S28) ··········· p. S30
5. Misfolded structures and corresponding secondary structures forming the first clusters for 1ESY and 1R7W (Figure S29) ··········· p. S31
6. Trajectories of 1-μs molecular dynamics (MD) simulations for three class I stem-loops (1F85, 1FHK, and 2KOC) and one class II stem-loop (1R2P) using the DESRES-RNA force field and the TIP4P-D explicit solvent model (Figure S30) ··········· p. S32
7. Comparison of non-canonical and transient base pairing with NMR data ··········· p. S33–S35
8. Trajectories of the rU_{40} single-stranded RNA end-to-end distances in the molecular dynamics simulations with the DESRES-RNA and AMBER-OL3 force fields in the GB-neck2 implicit solvent environment at a 0.15 M ionic concentration (Figure S31) ··········· p. S36
9. Explicit water molecular dynamics simulation of an RNA stem-loop in the presence of divalent

magnesium ions (Methods, Results and Discussion) ···································p. S37–S38

10. Trajectories of 1-μs molecular dynamics (MD) simulations for class II stem-loops 1R2P using the DESRES-RNA force field and the TIP4P-D explicit solvent model in the absence and presence of magnesium ions (Figure S32) ···p. S39
11. Comparison of overall, loop, and bulge structures of the class II 1R2P stem-loop obtained from NMR, molecular dynamics (MD) simulations with the TIP4P-D explicit solvent model in the absence and presence of magnesium ions, and MD simulations with the GB-neck2 implicit solvent model. (Figure S33) ···p. S40
12. Molecular dynamics simulations of RNA duplex formation (Models and Methods, Results and Discussion) ·······································p. S41–S42
13. Trajectories of three independent molecular dynamics simulations of CGCGG RNA duplex formation (Figure S34) ··p. S43
14. Trajectories of three independent molecular dynamics simulations of ACUGUCA RNA duplex formation (Figure S35) ··p. S44
15. Trajectories of three independent molecular dynamics simulations of CGACGCAG RNA duplex formation (Figure S36) ··p. S45
16. Non-hydrogen atom root mean square deviation, base pairing, and C3′-endo sugar pucker presence evaluated over the last 0.5 μs of simulations for the CGCGG, ACUCUCA, and CGACGCAG RNA duplex models (Table S1) ···p. S46
17. Descriptions of simulation movies (Movies S1, S2, and S3) ·······························p. S47
18. Input files used for AMBER molecular dynamics simulations ·····························p. S48
19. SI References ···p. S49

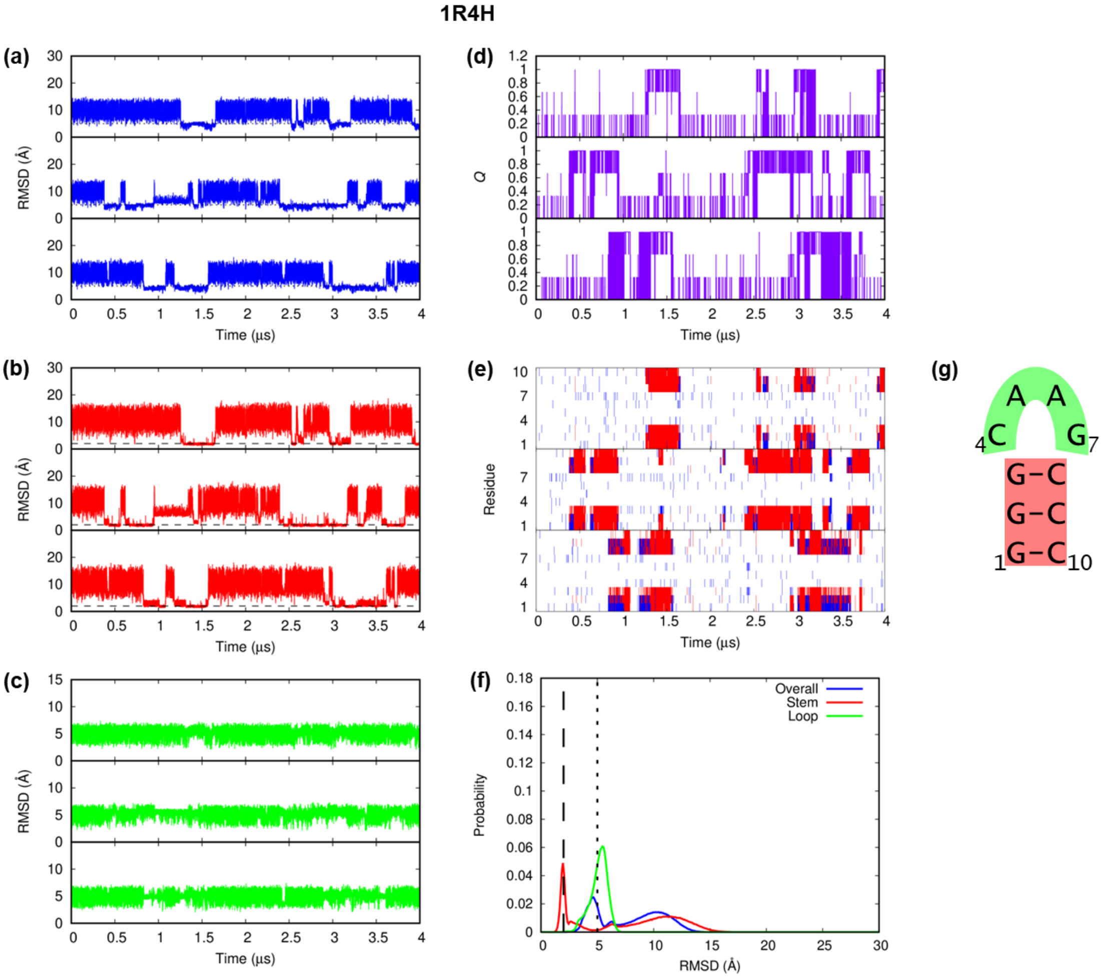

Figure S1. Trajectories of three independent molecular dynamics (MD) simulations of 1R4H. (a) Root mean square deviation (RMSD) of the entire molecule. The dotted horizontal lines indicate the RMSD at 5 Å. (b) RMSD of the stem region. The dashed horizontal lines indicate the RMSD at 2 Å. (c) RMSD of the loop region. (d) The fraction of native base pairs, Q. (e) Base pairs formed during simulations. The native and non-native base pairs are represented in red and blue, respectively. (f) Probability distributions of RMSD values for the entire molecule (blue), stem (red), and loop (green) regions. The dashed and dotted vertical lines in the probability distribution plot indicate the RMSD values at 2 and 5 Å, respectively. (g) Secondary structure of the RNA model. The stem and loop regions are indicated in red and green, respectively. In plots (a)–(e), the three sub-panels from top to bottom correspond to the first, second, and third MD simulations, respectively.

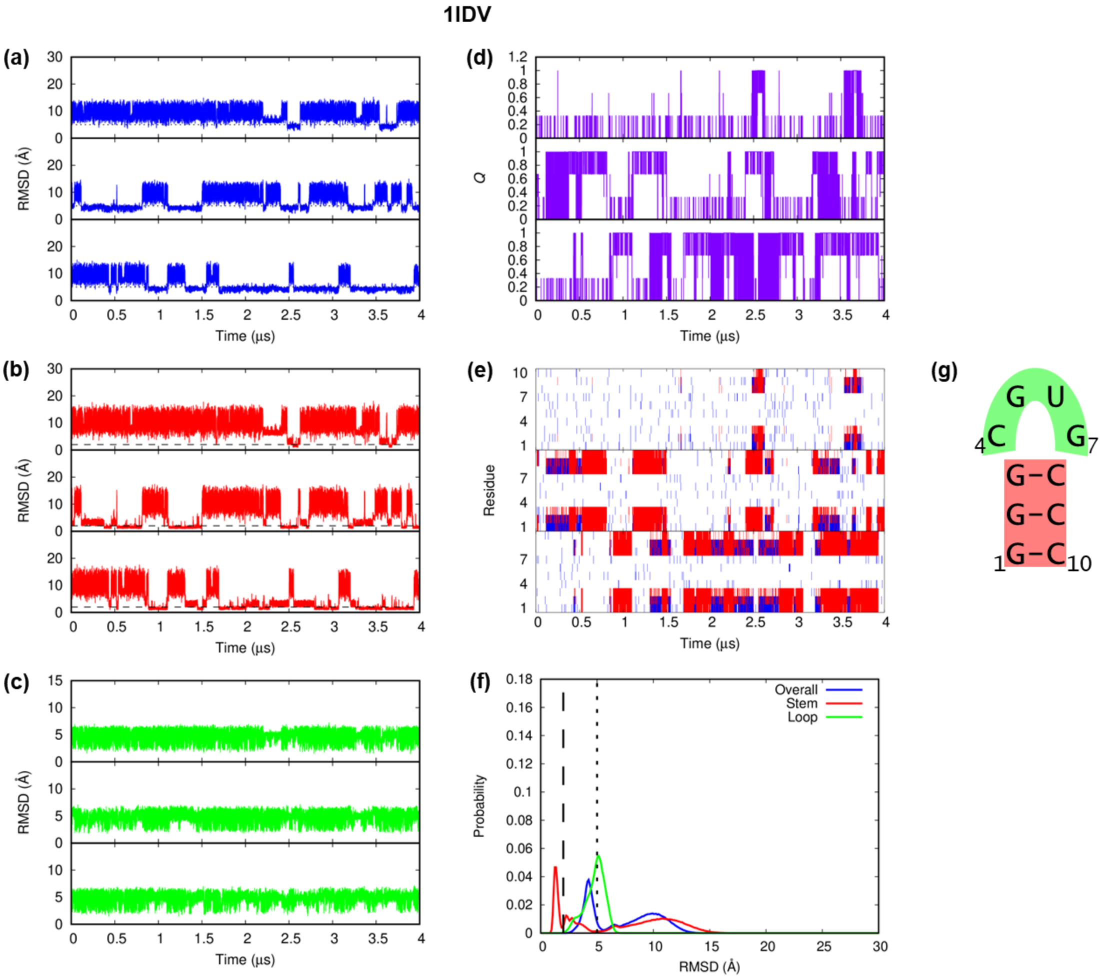

Figure S2. Trajectories of three independent molecular dynamics (MD) simulations of 1IDV. (a) Root mean square deviation (RMSD) of the entire molecule. The dotted horizontal lines indicate the RMSD at 5 Å. (b) RMSD of the stem region. The dashed horizontal lines indicate the RMSD at 2 Å. (c) RMSD of the loop region. (d) The fraction of native base pairs, Q. (e) Base pairs formed during simulations. The native and non-native base pairs are represented in red and blue, respectively. (f) Probability distributions of RMSD values for the entire molecule (blue), stem (red), and loop (green) regions. The dashed and dotted vertical lines in the probability distribution plot indicate the RMSD values at 2 and 5 Å, respectively. (g) Secondary structure of the RNA model. The stem and loop regions are indicated in red and green, respectively. In plots (a)–(e), the three sub-panels from top to bottom correspond to the first, second, and third MD simulations, respectively.

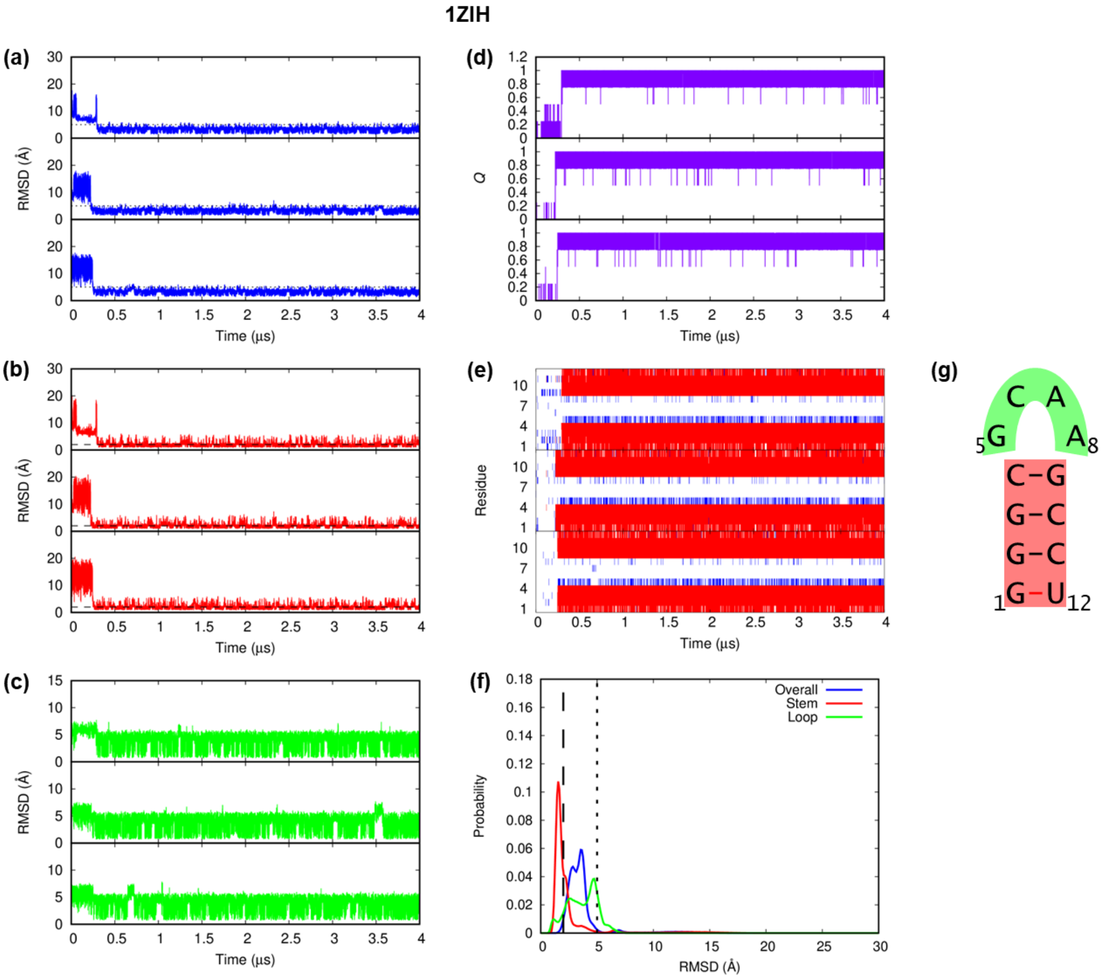

Figure S3. Trajectories of three independent molecular dynamics (MD) simulations of 1ZIH. (a) Root mean square deviation (RMSD) of the entire molecule. The dotted horizontal lines indicate the RMSD at 5 Å. (b) RMSD of the stem region. The dashed horizontal lines indicate the RMSD at 2 Å. (c) RMSD of the loop region. (d) The fraction of native base pairs, Q. (e) Base pairs formed during simulations. The native and non-native base pairs are represented in red and blue, respectively. (f) Probability distributions of RMSD values for the entire molecule (blue), stem (red), and loop (green) regions. The dashed and dotted vertical lines in the probability distribution plot indicate the RMSD values at 2 and 5 Å, respectively. (g) Secondary structure of the RNA model. The stem and loop regions are indicated in red and green, respectively. In plots (a)–(e), the three sub-panels from top to bottom correspond to the first, second, and third MD simulations, respectively.

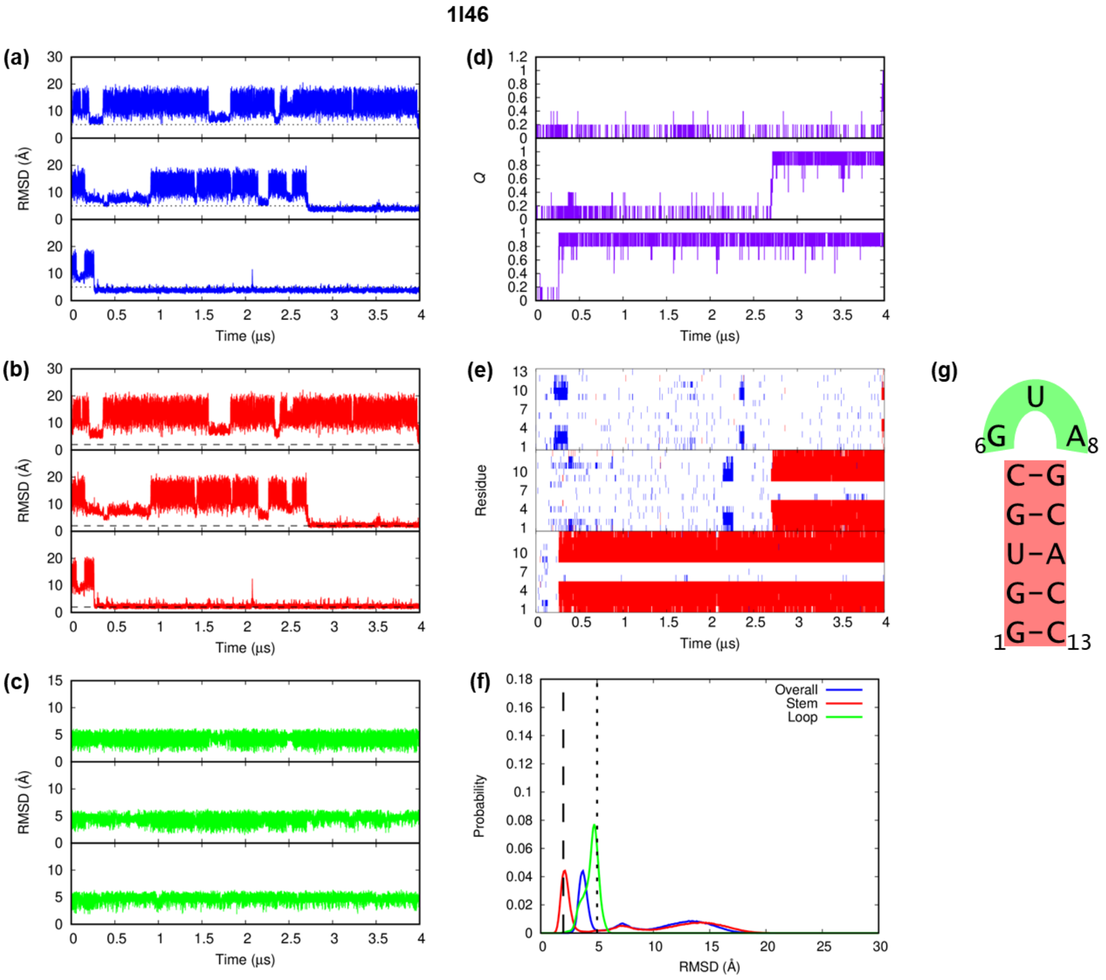

Figure S4. Trajectories of three independent molecular dynamics (MD) simulations of 1I46. (a) Root mean square deviation (RMSD) of the entire molecule. The dotted horizontal lines indicate the RMSD at 5 Å. (b) RMSD of the stem region. The dashed horizontal lines indicate the RMSD at 2 Å. (c) RMSD of the loop region. (d) The fraction of native base pairs, Q. (e) Base pairs formed during simulations. The native and non-native base pairs are represented in red and blue, respectively. (f) Probability distributions of RMSD values for the entire molecule (blue), stem (red), and loop (green) regions. The dashed and dotted vertical lines in the probability distribution plot indicate the RMSD values at 2 and 5 Å, respectively. (g) Secondary structure of the RNA model. The stem and loop regions are indicated in red and green, respectively. In plots (a)–(e), the three sub-panels from top to bottom correspond to the first, second, and third MD simulations, respectively.

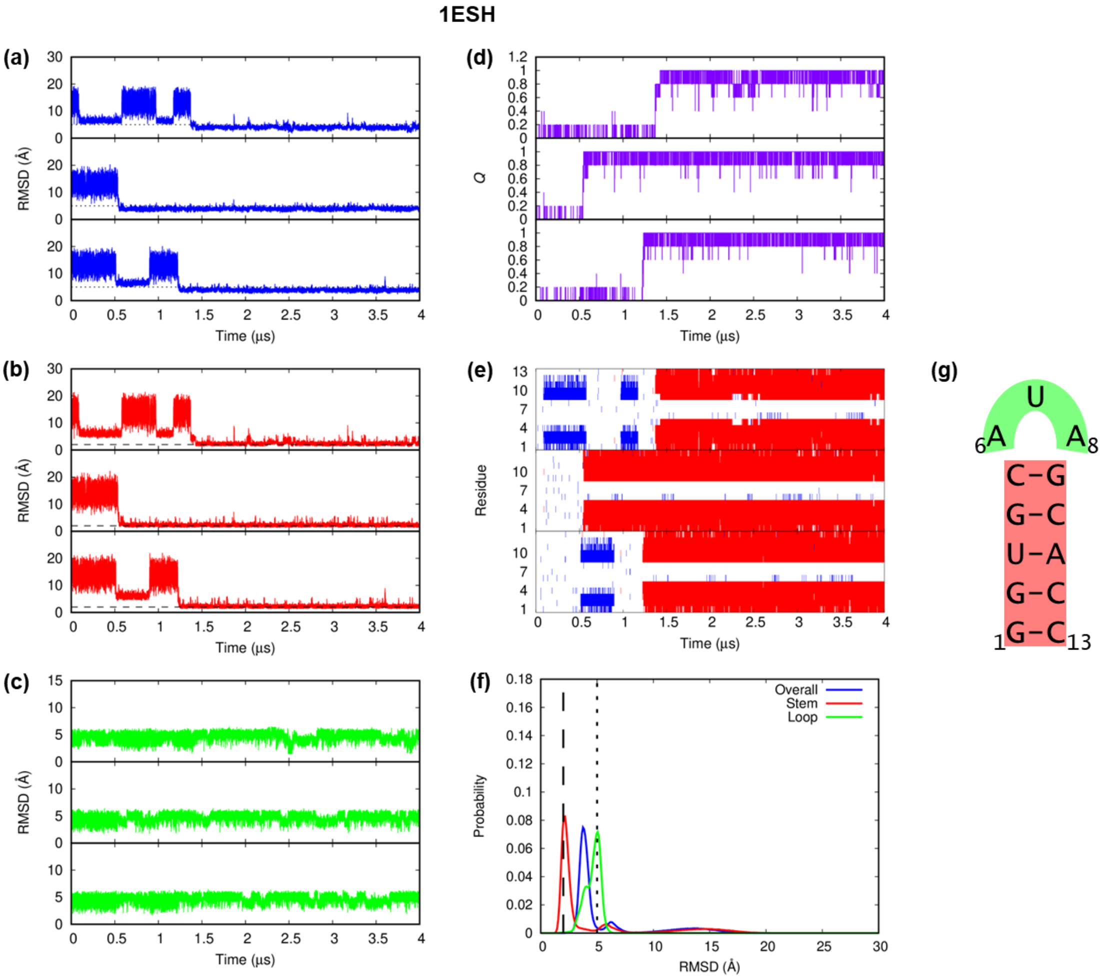

Figure S5. Trajectories of three independent molecular dynamics (MD) simulations of 1ESH. (a) Root mean square deviation (RMSD) of the entire molecule. The dotted horizontal lines indicate the RMSD at 5 Å. (b) RMSD of the stem region. The dashed horizontal lines indicate the RMSD at 2 Å. (c) RMSD of the loop region. (d) The fraction of native base pairs, Q. (e) Base pairs formed during simulations. The native and non-native base pairs are represented in red and blue, respectively. (f) Probability distributions of RMSD values for the entire molecule (blue), stem (red), and loop (green) regions. The dashed and dotted vertical lines in the probability distribution plot indicate the RMSD values at 2 and 5 Å, respectively. (g) Secondary structure of the RNA model. The stem and loop regions are indicated in red and green, respectively. In plots (a)–(e), the three sub-panels from top to bottom correspond to the first, second, and third MD simulations, respectively.

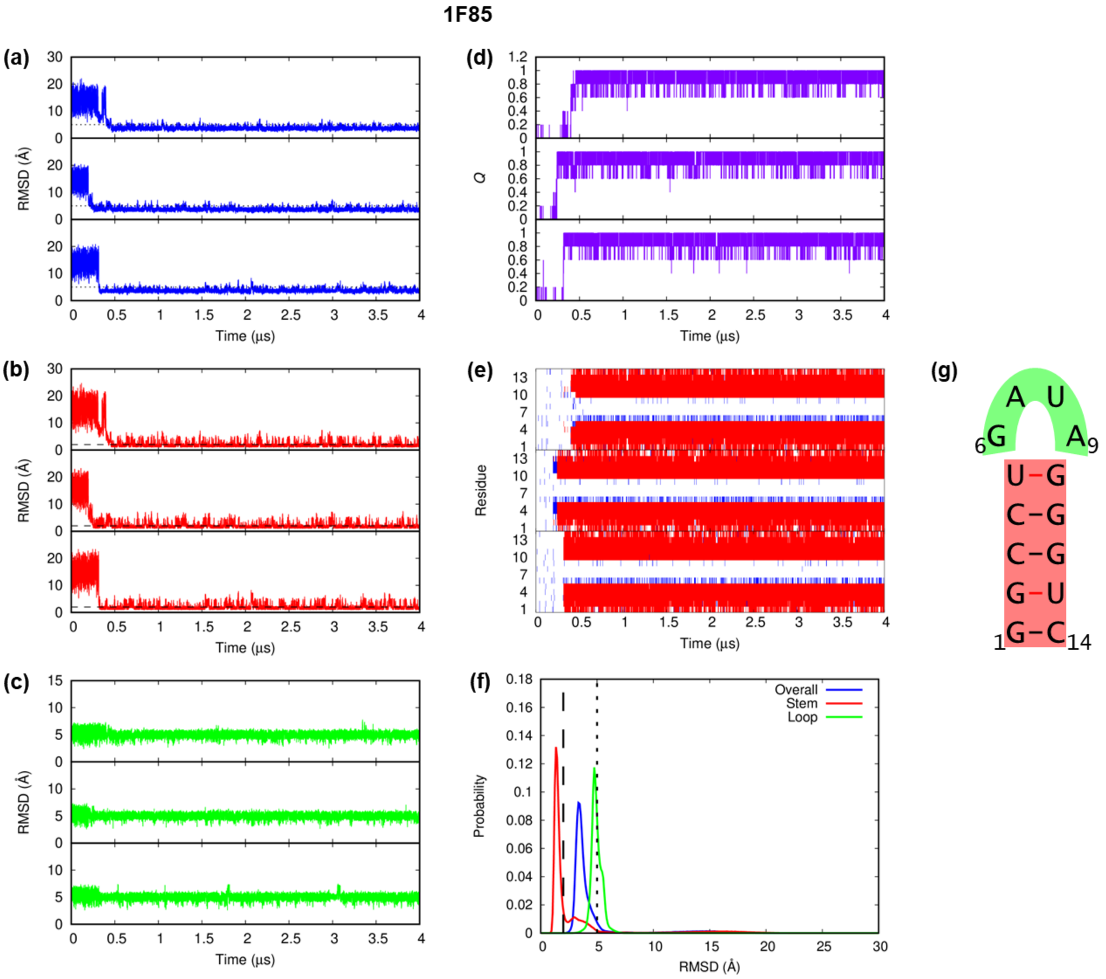

Figure S6. Trajectories of three independent molecular dynamics (MD) simulations of 1F85. (a) Root mean square deviation (RMSD) of the entire molecule. The dotted horizontal lines indicate the RMSD at 5 Å. (b) RMSD of the stem region. The dashed horizontal lines indicate the RMSD at 2 Å. (c) RMSD of the loop region. (d) The fraction of native base pairs, Q. (e) Base pairs formed during simulations. The native and non-native base pairs are represented in red and blue, respectively. (f) Probability distributions of RMSD values for the entire molecule (blue), stem (red), and loop (green) regions. The dashed and dotted vertical lines in the probability distribution plot indicate the RMSD values at 2 and 5 Å, respectively. (g) Secondary structure of the RNA model. The stem and loop regions are indicated in red and green, respectively. In plots (a)–(e), the three sub-panels from top to bottom correspond to the first, second, and third MD simulations, respectively.

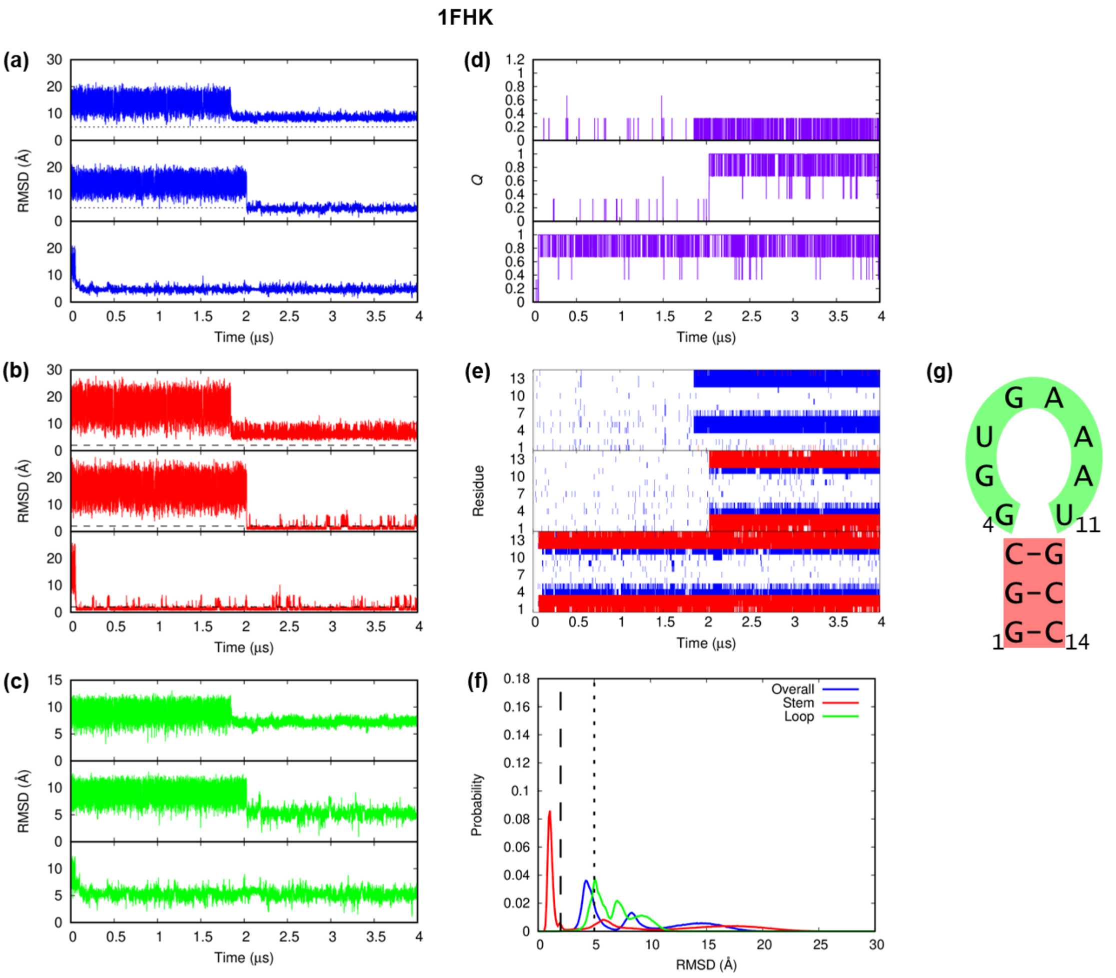

Figure S7. Trajectories of three independent molecular dynamics (MD) simulations of 1FHK. (a) Root mean square deviation (RMSD) of the entire molecule. The dotted horizontal lines indicate the RMSD at 5 Å. (b) RMSD of the stem region. The dashed horizontal lines indicate the RMSD at 2 Å. (c) RMSD of the loop region. (d) The fraction of native base pairs, Q. (e) Base pairs formed during simulations. The native and non-native base pairs are represented in red and blue, respectively. (f) Probability distributions of RMSD values for the entire molecule (blue), stem (red), and loop (green) regions. The dashed and dotted vertical lines in the probability distribution plot indicate the RMSD values at 2 and 5 Å, respectively. (g) Secondary structure of the RNA model. The stem and loop regions are indicated in red and green, respectively. In plots (a)–(e), the three sub-panels from top to bottom correspond to the first, second, and third MD simulations, respectively.

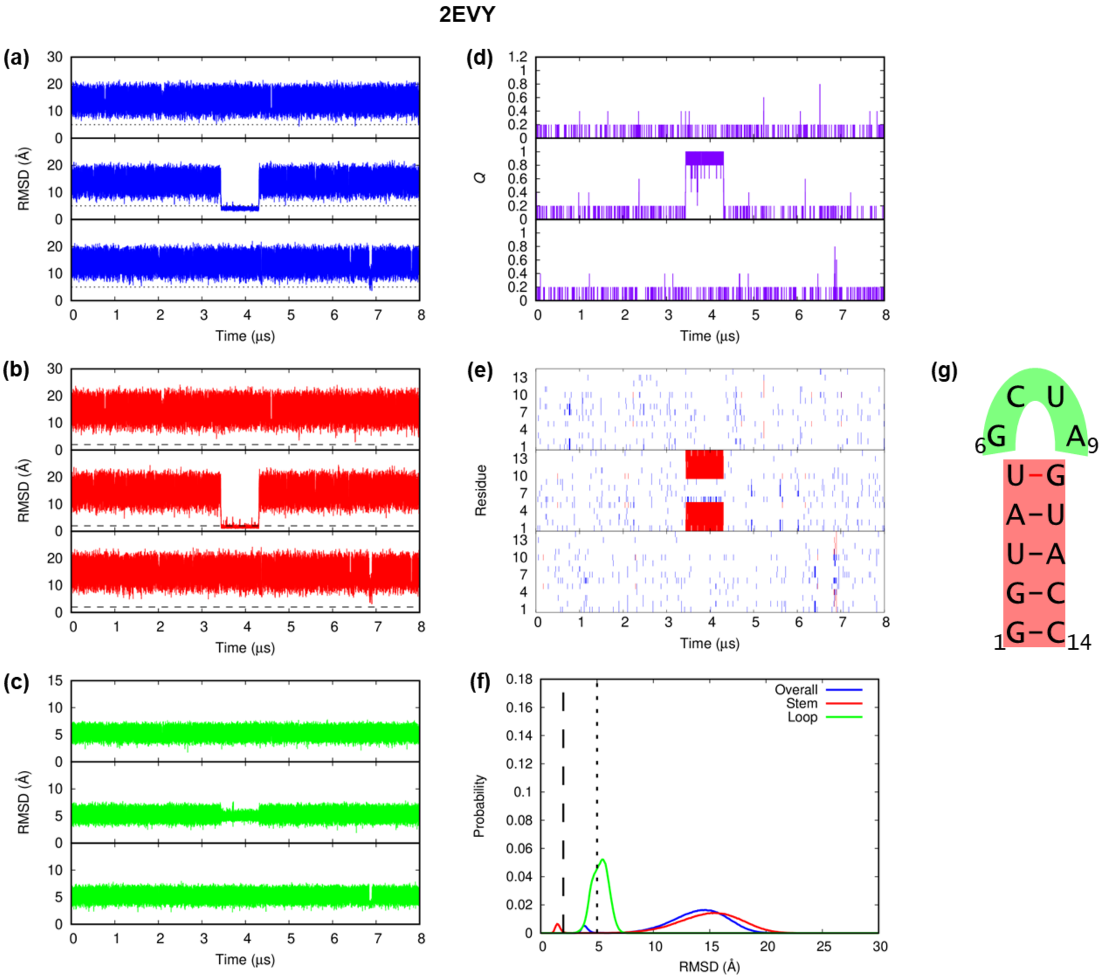

Figure S8. Trajectories of three independent molecular dynamics (MD) simulations of 2EVY. (a) Root mean square deviation (RMSD) of the entire molecule. The dotted horizontal lines indicate the RMSD at 5 Å. (b) RMSD of the stem region. The dashed horizontal lines indicate the RMSD at 2 Å. (c) RMSD of the loop region. (d) The fraction of native base pairs, Q. (e) Base pairs formed during simulations. The native and non-native base pairs are represented in red and blue, respectively. (f) Probability distributions of RMSD values for the entire molecule (blue), stem (red), and loop (green) regions. The dashed and dotted vertical lines in the probability distribution plot indicate the RMSD values at 2 and 5 Å, respectively. (g) Secondary structure of the RNA model. The stem and loop regions are indicated in red and green, respectively. In plots (a)–(e), the three sub-panels from top to bottom correspond to the first, second, and third MD simulations, respectively.

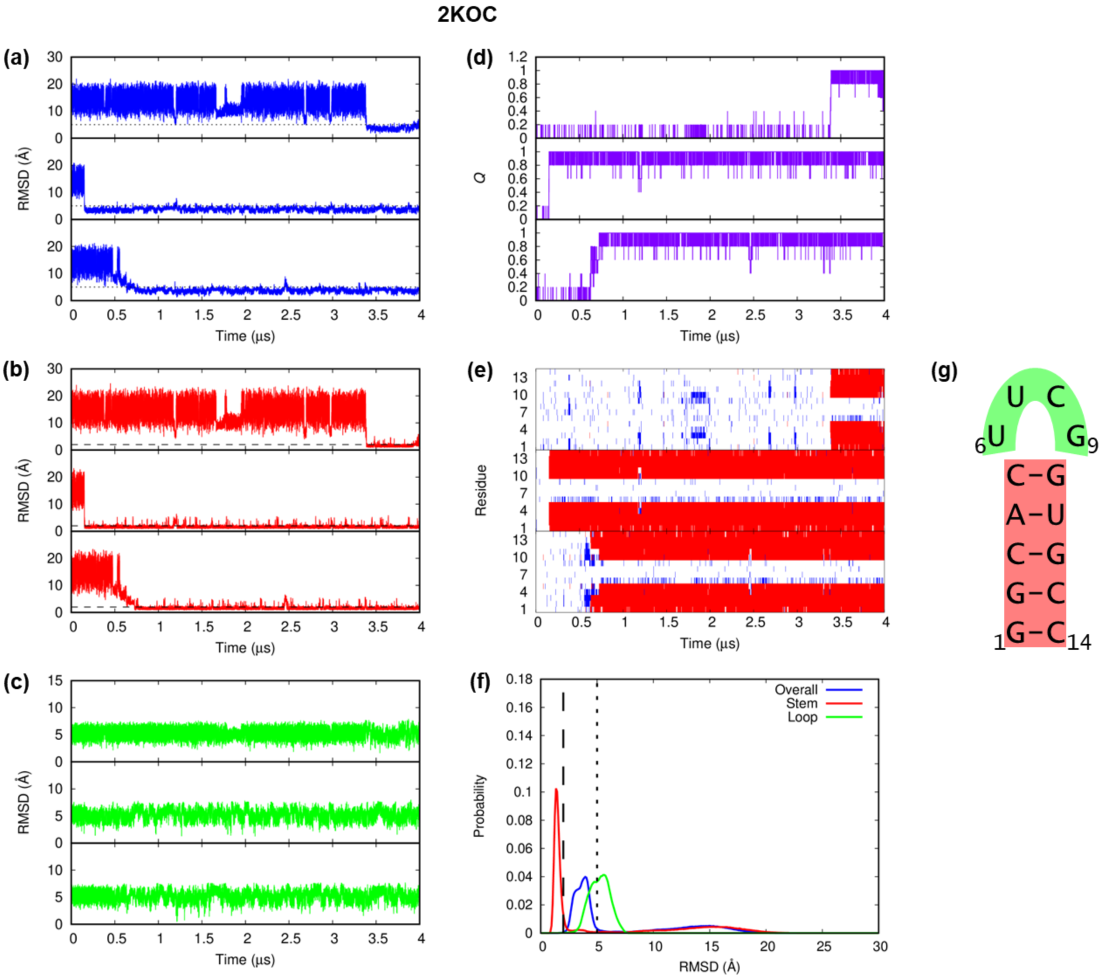

Figure S9. Trajectories of three independent molecular dynamics (MD) simulations of 2KOC. (a) Root mean square deviation (RMSD) of the entire molecule. The dotted horizontal lines indicate the RMSD at 5 Å. (b) RMSD of the stem region. The dashed horizontal lines indicate the RMSD at 2 Å. (c) RMSD of the loop region. (d) The fraction of native base pairs, Q. (e) Base pairs formed during simulations. The native and non-native base pairs are represented in red and blue, respectively. (f) Probability distributions of RMSD values for the entire molecule (blue), stem (red), and loop (green) regions. The dashed and dotted vertical lines in the probability distribution plot indicate the RMSD values at 2 and 5 Å, respectively. (g) Secondary structure of the RNA model. The stem and loop regions are indicated in red and green, respectively. In plots (a)–(e), the three sub-panels from top to bottom correspond to the first, second, and third MD simulations, respectively.

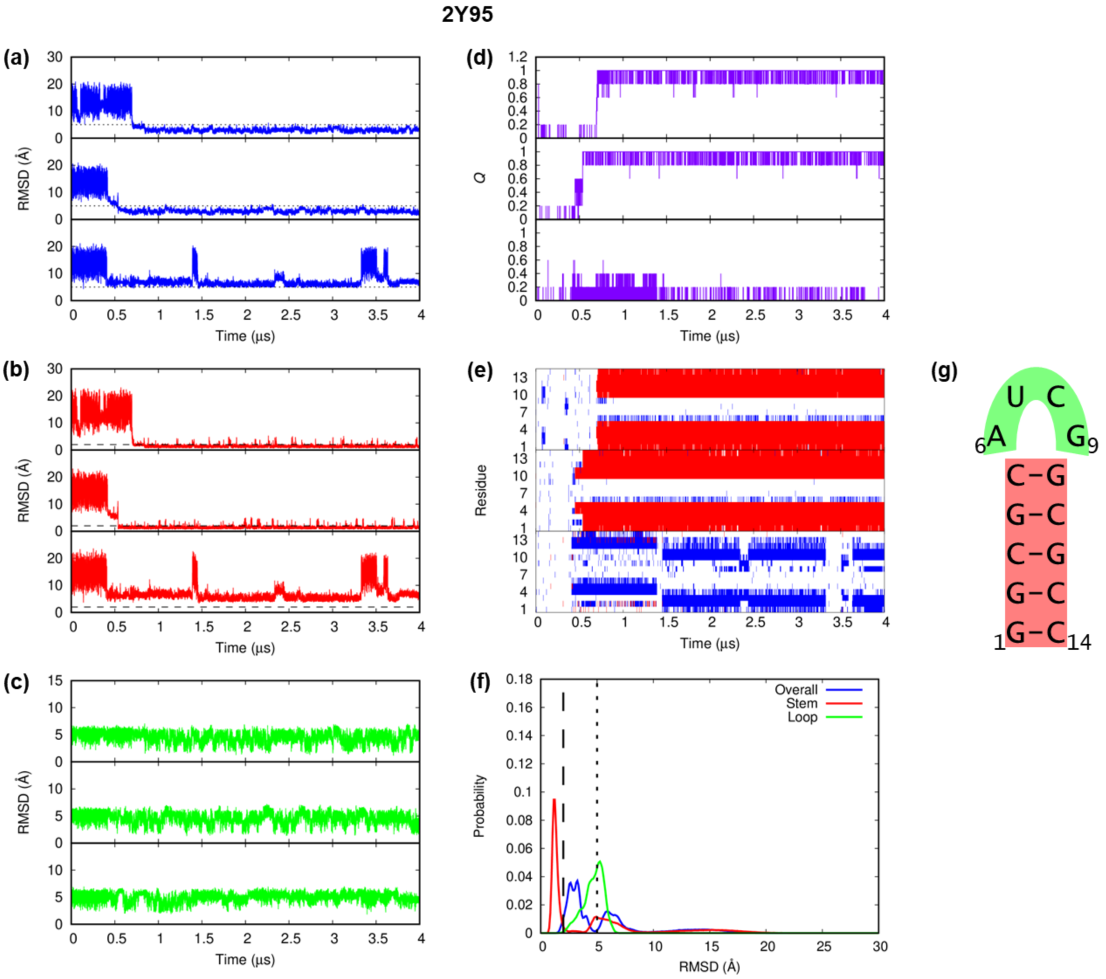

Figure S10. Trajectories of three independent molecular dynamics (MD) simulations of 2Y95. (a) Root mean square deviation (RMSD) of the entire molecule. The dotted horizontal lines indicate the RMSD at 5 Å. (b) RMSD of the stem region. The dashed horizontal lines indicate the RMSD at 2 Å. (c) RMSD of the loop region. (d) The fraction of native base pairs, Q. (e) Base pairs formed during simulations. The native and non-native base pairs are represented in red and blue, respectively. (f) Probability distributions of RMSD values for the entire molecule (blue), stem (red), and loop (green) regions. The dashed and dotted vertical lines in the probability distribution plot indicate the RMSD values at 2 and 5 Å, respectively. (g) Secondary structure of the RNA model. The stem and loop regions are indicated in red and green, respectively. In plots (a)–(e), the three sub-panels from top to bottom correspond to the first, second, and third MD simulations, respectively.

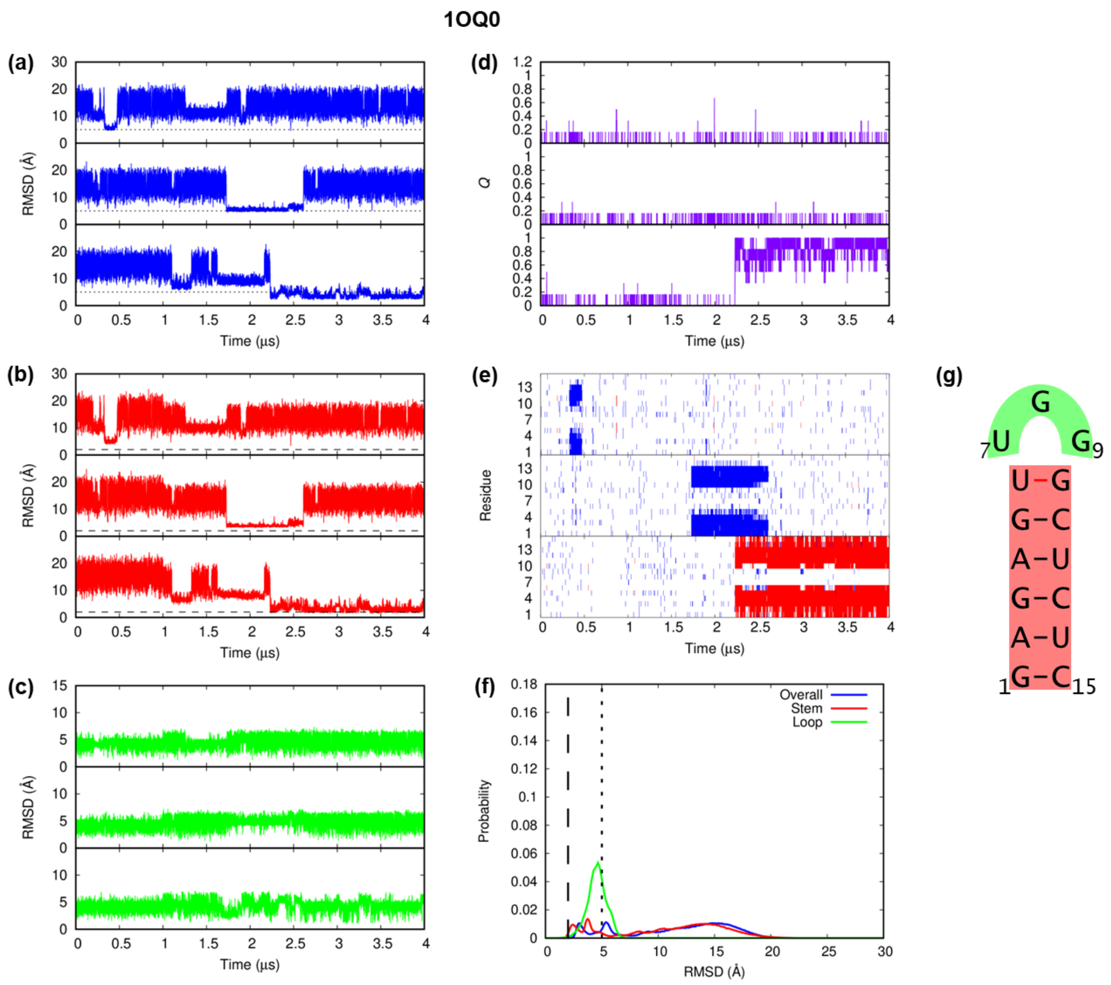

Figure S11. Trajectories of three independent molecular dynamics (MD) simulations of 1OQ0. (a) Root mean square deviation (RMSD) of the entire molecule. The dotted horizontal lines indicate the RMSD at 5 Å. (b) RMSD of the stem region. The dashed horizontal lines indicate the RMSD at 2 Å. (c) RMSD of the loop region. (d) The fraction of native base pairs, Q. (e) Base pairs formed during simulations. The native and non-native base pairs are represented in red and blue, respectively. (f) Probability distributions of RMSD values for the entire molecule (blue), stem (red), and loop (green) regions. The dashed and dotted vertical lines in the probability distribution plot indicate the RMSD values at 2 and 5 Å, respectively. (g) Secondary structure of the RNA model. The stem and loop regions are indicated in red and green, respectively. In plots (a)–(e), the three sub-panels from top to bottom correspond to the first, second, and third MD simulations, respectively.

1XWP

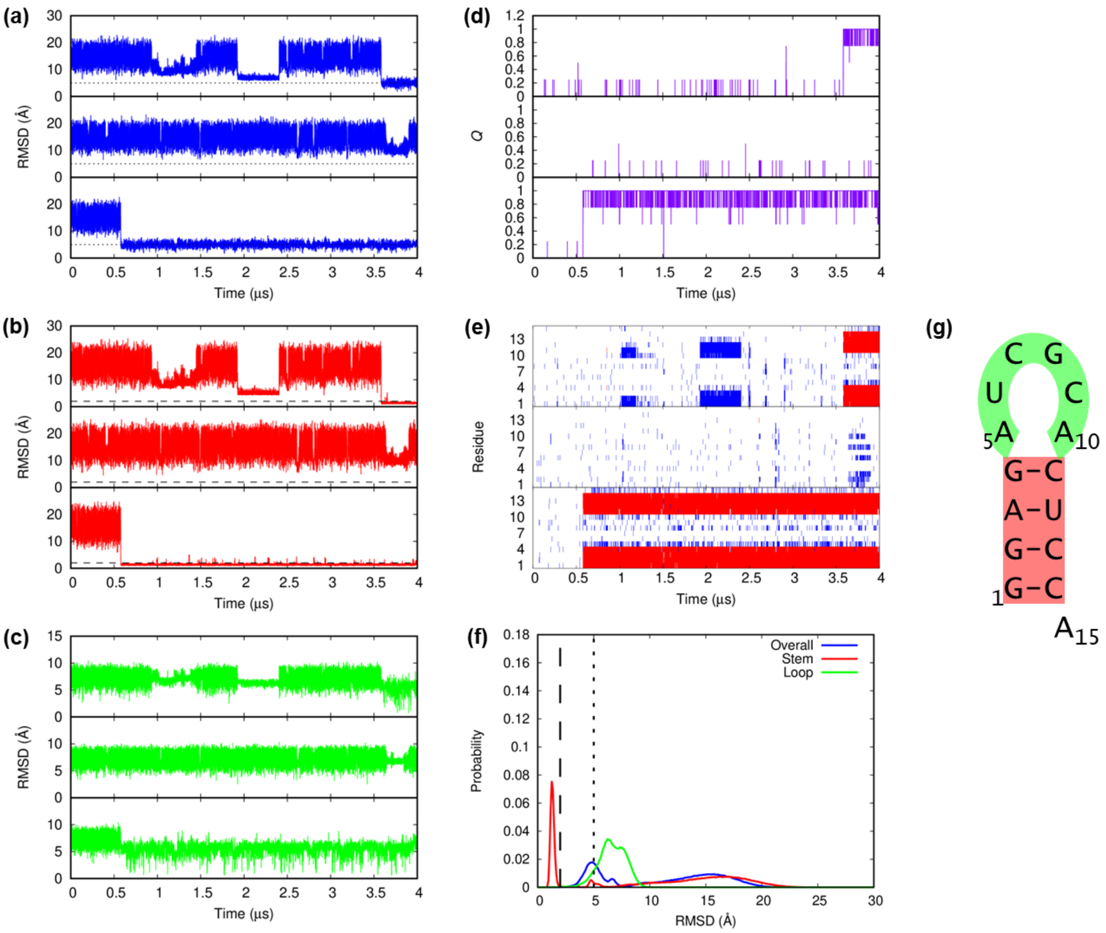

Figure S12. Trajectories of three independent molecular dynamics (MD) simulations of 1XWP. (a) Root mean square deviation (RMSD) of the entire molecule. The dotted horizontal lines indicate the RMSD at 5 Å. (b) RMSD of the stem region. The dashed horizontal lines indicate the RMSD at 2 Å. (c) RMSD of the loop region. (d) The fraction of native base pairs, Q. (e) Base pairs formed during simulations. The native and non-native base pairs are represented in red and blue, respectively. (f) Probability distributions of RMSD values for the entire molecule (blue), stem (red), and loop (green) regions. The dashed and dotted vertical lines in the probability distribution plot indicate the RMSD values at 2 and 5 Å, respectively. (g) Secondary structure of the RNA model. The stem and loop regions are indicated in red and green, respectively. In plots (a)–(e), the three sub-panels from top to bottom correspond to the first, second, and third MD simulations, respectively.

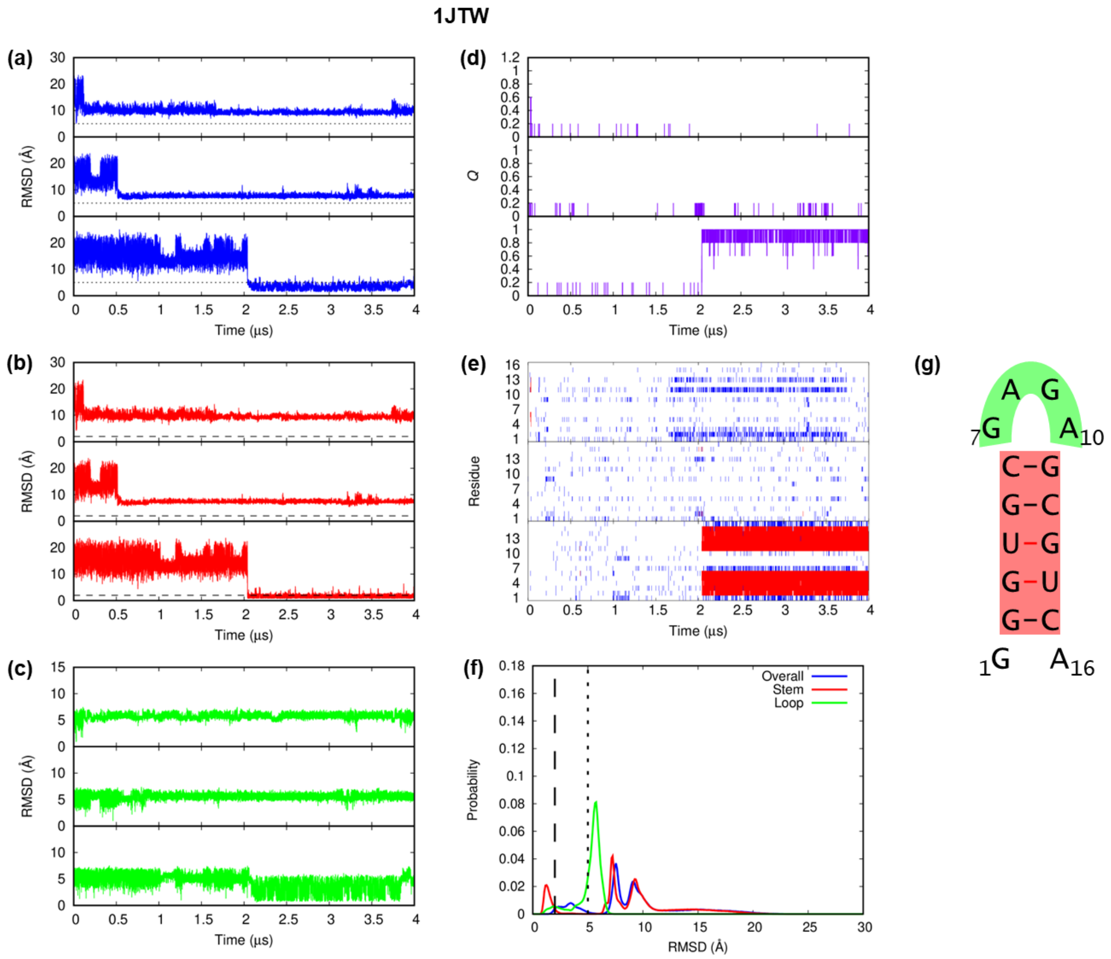

Figure S13. Trajectories of three independent molecular dynamics (MD) simulations of 1JTW. (a) Root mean square deviation (RMSD) of the entire molecule. The dotted horizontal lines indicate the RMSD at 5 Å. (b) RMSD of the stem region. The dashed horizontal lines indicate the RMSD at 2 Å. (c) RMSD of the loop region. (d) The fraction of native base pairs, Q. (e) Base pairs formed during simulations. The native and non-native base pairs are represented in red and blue, respectively. (f) Probability distributions of RMSD values for the entire molecule (blue), stem (red), and loop (green) regions. The dashed and dotted vertical lines in the probability distribution plot indicate the RMSD values at 2 and 5 Å, respectively. (g) Secondary structure of the RNA model. The stem and loop regions are indicated in red and green, respectively. In plots (a)–(e), the three sub-panels from top to bottom correspond to the first, second, and third MD simulations, respectively.

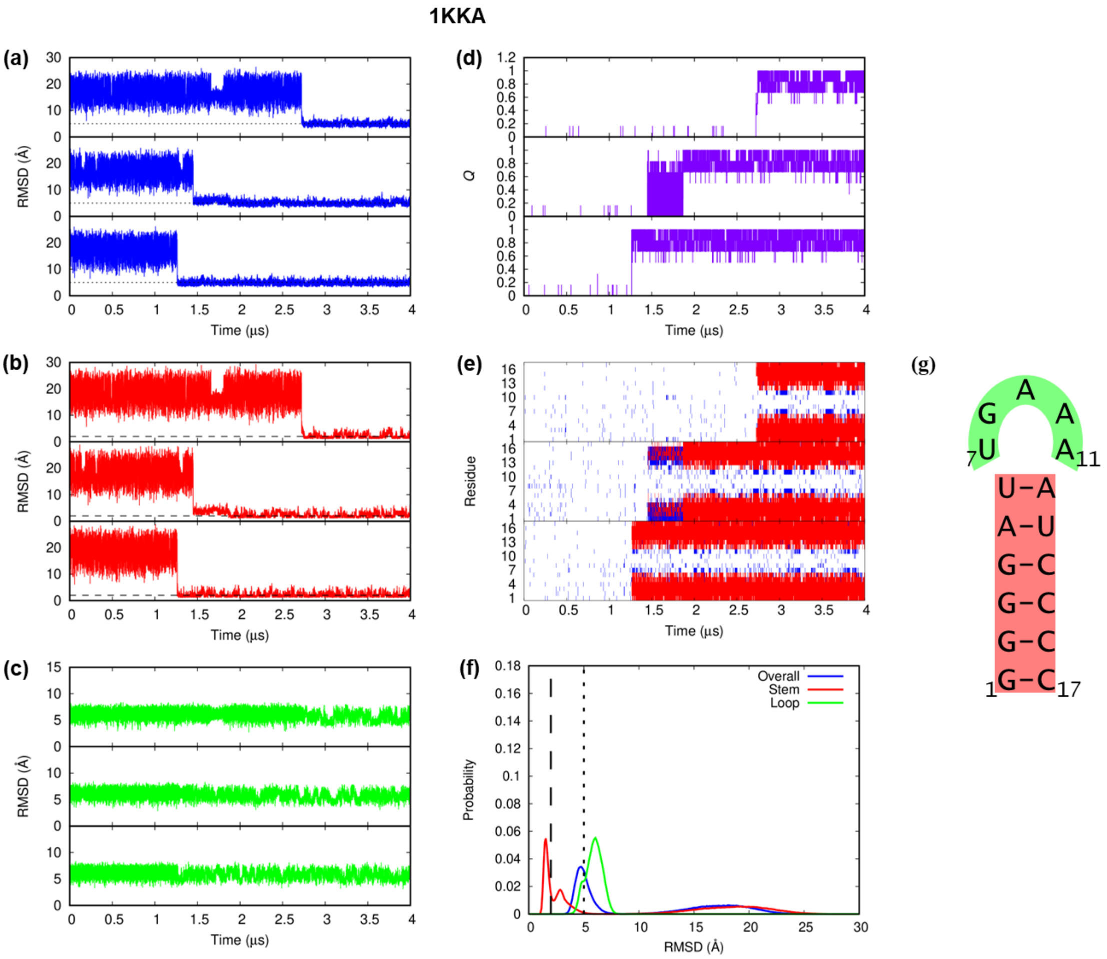

Figure S14. Trajectories of three independent molecular dynamics (MD) simulations of 1KKA. (a) Root mean square deviation (RMSD) of the entire molecule. The dotted horizontal lines indicate the RMSD at 5 Å. (b) RMSD of the stem region. The dashed horizontal lines indicate the RMSD at 2 Å. (c) RMSD of the loop region. (d) The fraction of native base pairs, Q. (e) Base pairs formed during simulations. The native and non-native base pairs are represented in red and blue, respectively. (f) Probability distributions of RMSD values for the entire molecule (blue), stem (red), and loop (green) regions. The dashed and dotted vertical lines in the probability distribution plot indicate the RMSD values at 2 and 5 Å, respectively. (g) Secondary structure of the RNA model. The stem and loop regions are indicated in red and green, respectively. In plots (a)–(e), the three sub-panels from top to bottom correspond to the first, second, and third MD simulations, respectively.

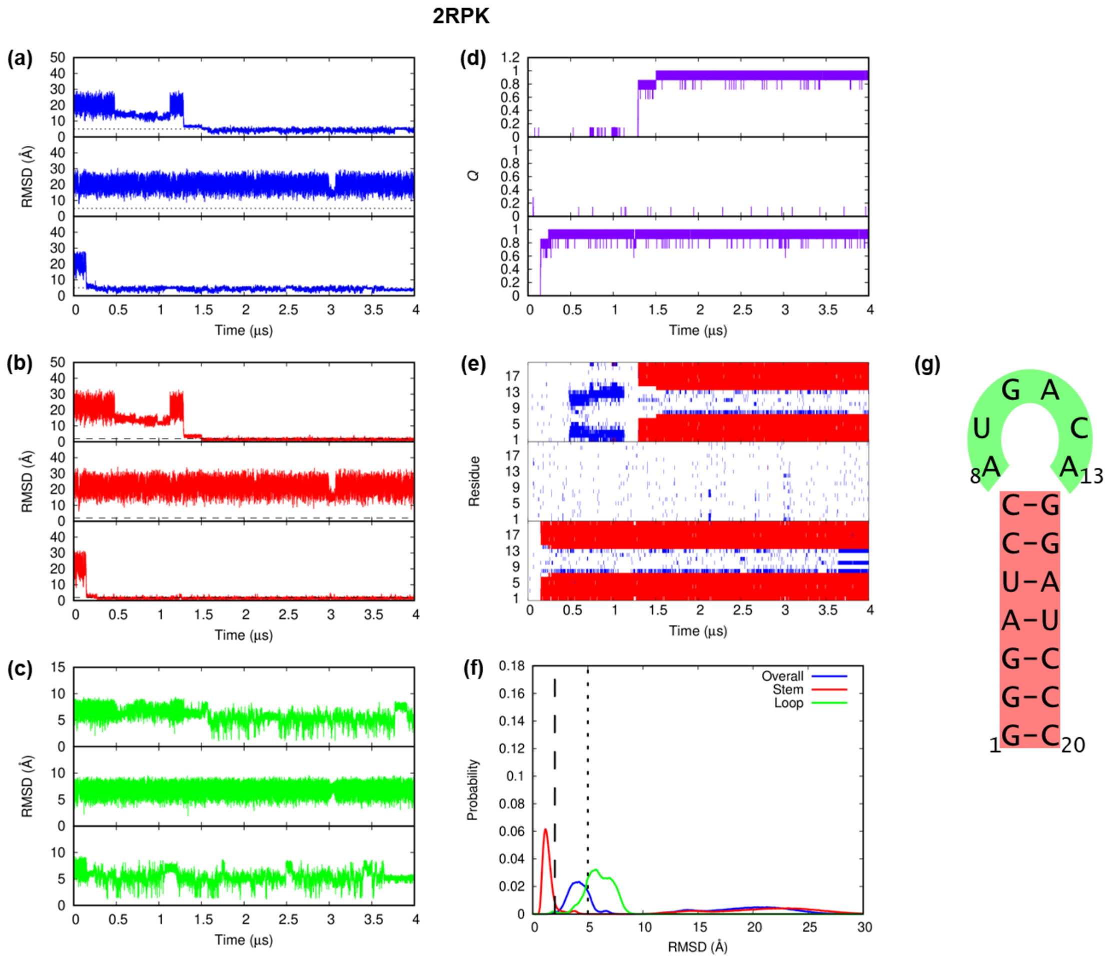

Figure S15. Trajectories of three independent molecular dynamics (MD) simulations of 2RPK. (a) Root mean square deviation (RMSD) of the entire molecule. The dotted horizontal lines indicate the RMSD at 5 Å. (b) RMSD of the stem region. The dashed horizontal lines indicate the RMSD at 2 Å. (c) RMSD of the loop region. (d) The fraction of native base pairs, Q. (e) Base pairs formed during simulations. The native and non-native base pairs are represented in red and blue, respectively. (f) Probability distributions of RMSD values for the entire molecule (blue), stem (red), and loop (green) regions. The dashed and dotted vertical lines in the probability distribution plot indicate the RMSD values at 2 and 5 Å, respectively. (g) Secondary structure of the RNA model. The stem and loop regions are indicated in red and green, respectively. In plots (a)–(e), the three sub-panels from top to bottom correspond to the first, second, and third MD simulations, respectively.

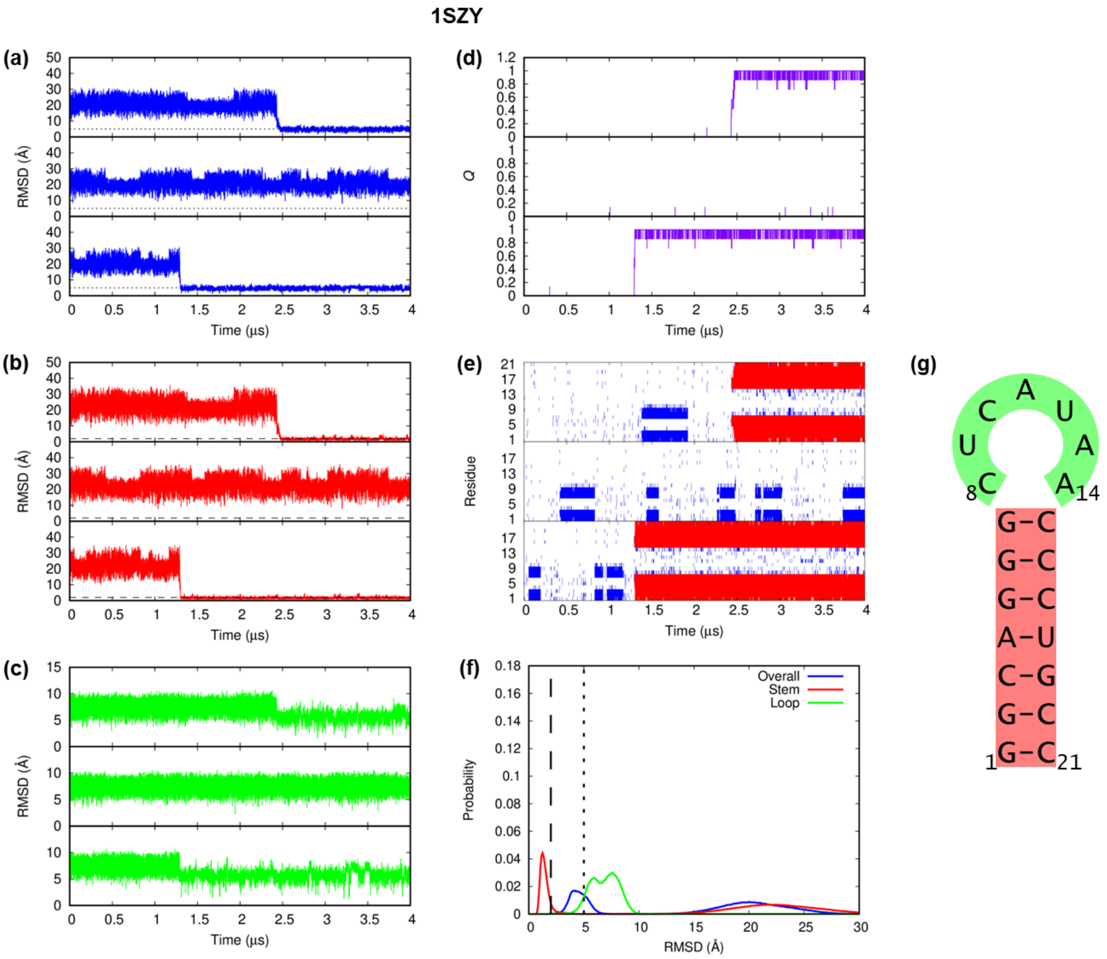

Figure S16. Trajectories of three independent molecular dynamics (MD) simulations of 1SZY. (a) Root mean square deviation (RMSD) of the entire molecule. The dotted horizontal lines indicate the RMSD at 5 Å. (b) RMSD of the stem region. The dashed horizontal lines indicate the RMSD at 2 Å. (c) RMSD of the loop region. (d) The fraction of native base pairs, Q. (e) Base pairs formed during simulations. The native and non-native base pairs are represented in red and blue, respectively. (f) Probability distributions of RMSD values for the entire molecule (blue), stem (red), and loop (green) regions. The dashed and dotted vertical lines in the probability distribution plot indicate the RMSD values at 2 and 5 Å, respectively. (g) Secondary structure of the RNA model. The stem and loop regions are indicated in red and green, respectively. In plots (a)–(e), the three sub-panels from top to bottom correspond to the first, second, and third MD simulations, respectively.

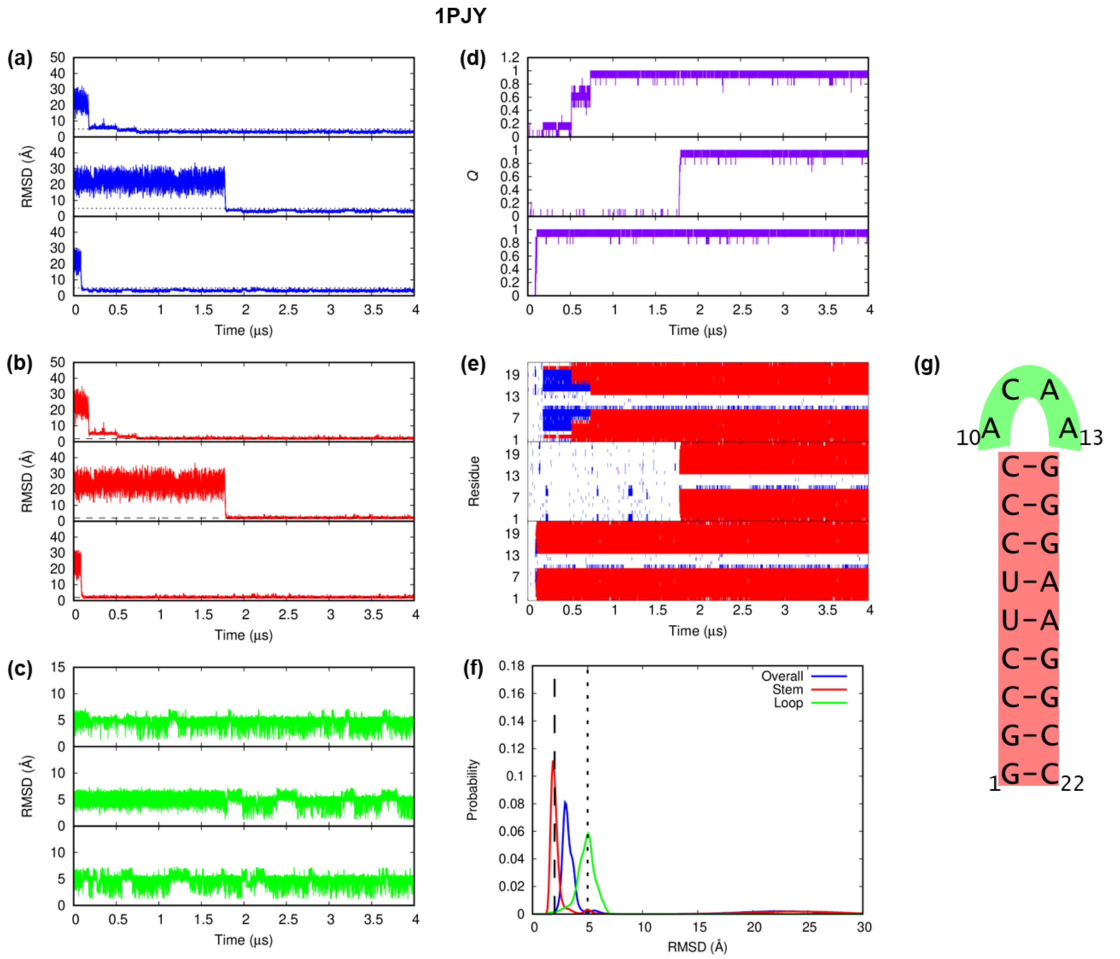

Figure S17. Trajectories of three independent molecular dynamics (MD) simulations of 1PJY. (a) Root mean square deviation (RMSD) of the entire molecule. The dotted horizontal lines indicate the RMSD at 5 Å. (b) RMSD of the stem region. The dashed horizontal lines indicate the RMSD at 2 Å. (c) RMSD of the loop region. (d) The fraction of native base pairs, Q. (e) Base pairs formed during simulations. The native and non-native base pairs are represented in red and blue, respectively. (f) Probability distributions of RMSD values for the entire molecule (blue), stem (red), and loop (green) regions. The dashed and dotted vertical lines in the probability distribution plot indicate the RMSD values at 2 and 5 Å, respectively. (g) Secondary structure of the RNA model. The stem and loop regions are indicated in red and green, respectively. In plots (a)–(e), the three sub-panels from top to bottom correspond to the first, second, and third MD simulations, respectively.

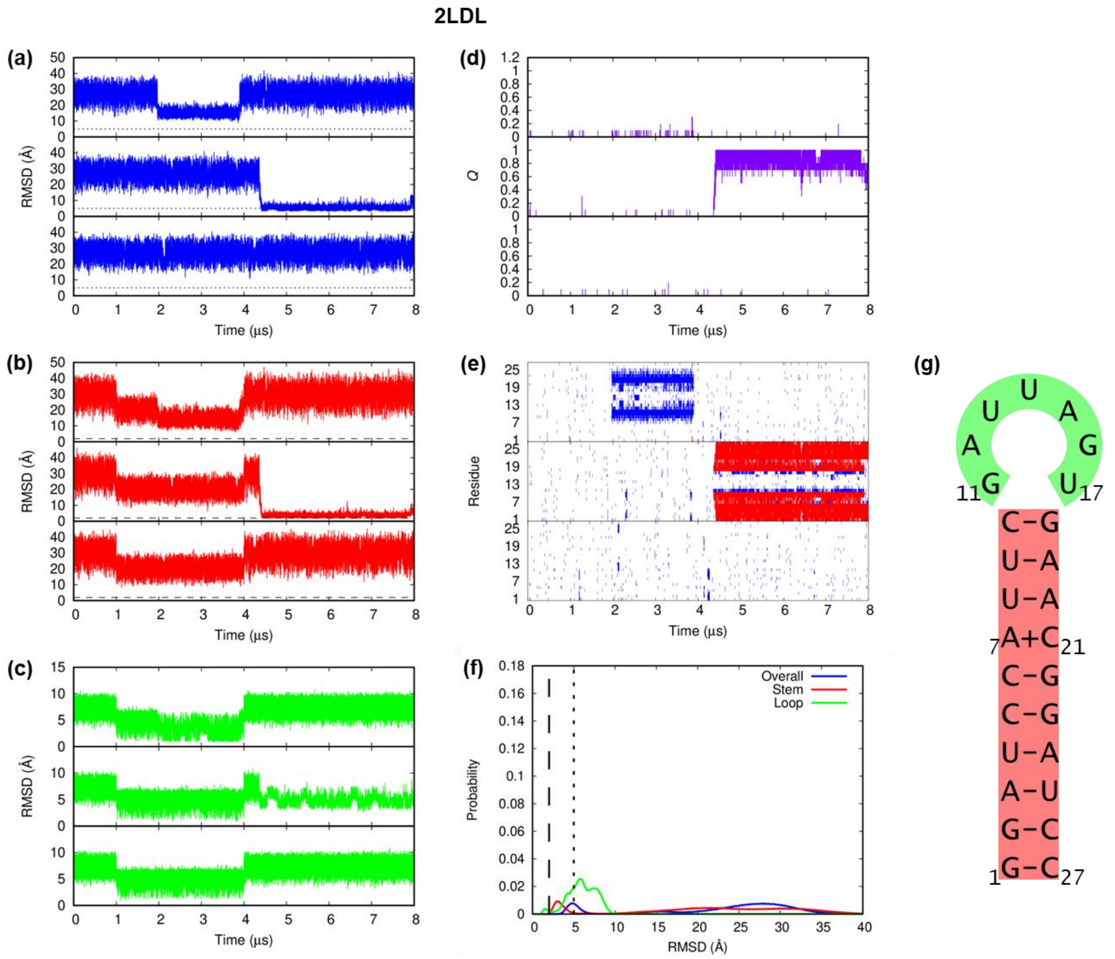

Figure S18. Trajectories of three independent molecular dynamics (MD) simulations of 2LDL. (a) Root mean square deviation (RMSD) RMSD of the entire molecule. The dotted horizontal lines indicate the RMSD at 5 Å. (b) RMSD of the stem region. The dashed horizontal lines indicate the RMSD at 2 Å. (c) RMSD of the loop region. (d) The fraction of native base pairs, *Q*. (e) Base pairs formed during simulations. The native and non-native base pairs are represented in red and blue, respectively. (f) Probability distributions of RMSD values for the entire molecule (blue), stem (red), and loop (green) regions. The dashed and dotted vertical lines in the probability distribution plot indicate the RMSD values at 2 and 5 Å, respectively. (g) Secondary structure of the RNA model. The stem and loop regions are indicated in red and green, respectively. In plots (a)–(e), the three sub-panels from top to bottom correspond to the first, second, and third MD simulations, respectively.

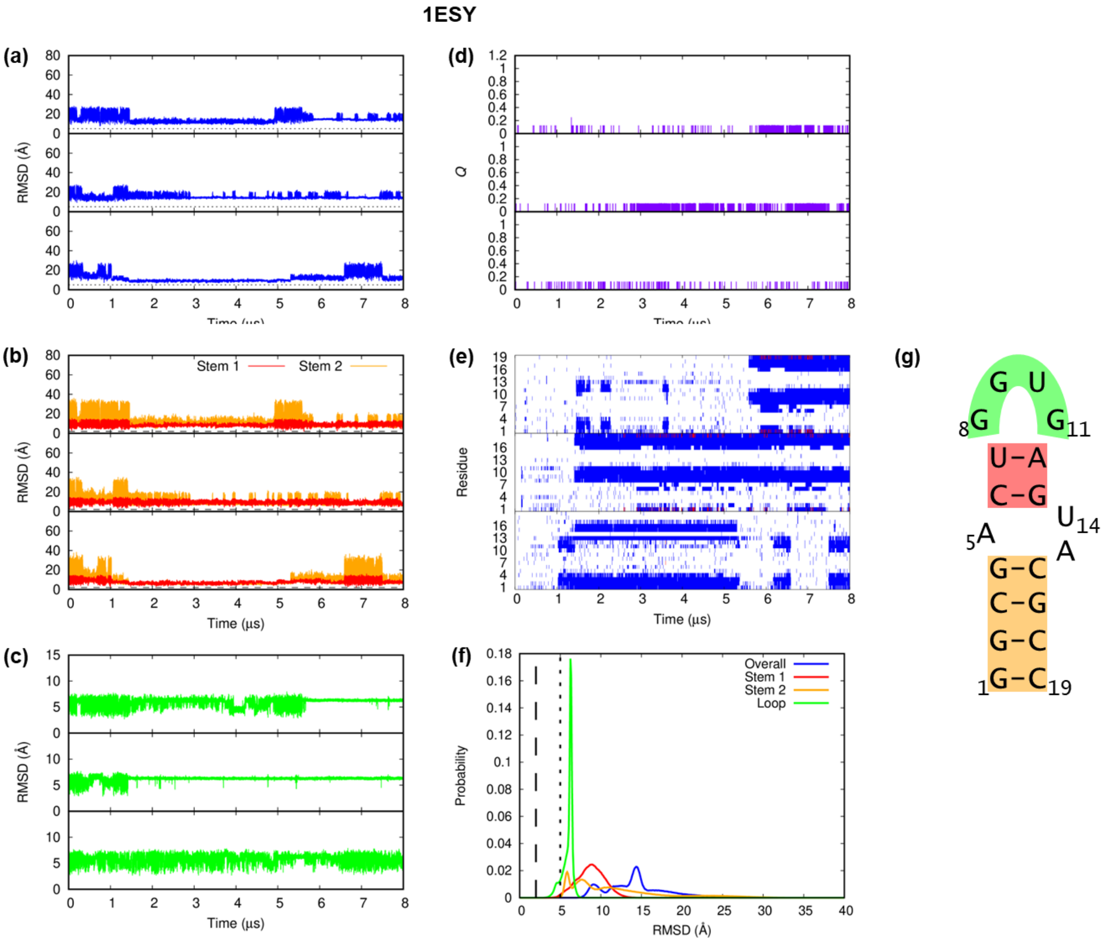

Figure S19. Trajectories of three independent molecular dynamics (MD) simulations of 1ESY. (a) Root mean square deviation (RMSD) of the entire molecule. The dotted horizontal lines indicate the RMSD at 5 Å. (b) RMSD of the two stem regions, where stem 1 (red lines) and stem 2 (orange lines) represent the double helical regions directly connecting the hairpin-loop and those near the 5′ and 3′ termini of the RNA, respectively. The dashed horizontal lines indicate the RMSD at 2 Å. (c) RMSD of the hairpin-loop region. (d) The fraction of native base pairs Q. (e) Base pairs formed during simulations, where the native and non-native base pairs are represented by red and blue, respectively. (f) Probability distributions of the RMSD values for the entire molecule (blue), stem 1 (red), stem 2 (orange), and hairpin-loop (green) regions. The dashed and dotted vertical lines in the probability distribution plot indicate the RMSD values at 2 and 5 Å, respectively. (g) Secondary structure of the RNA model. Stem 1, stem 2, and hairpin-loop regions are indicated in red, orange, and green, respectively. In plots (a)–(e), three sub-panels from top to bottom correspond to the first, second, and third MD simulations, respectively.

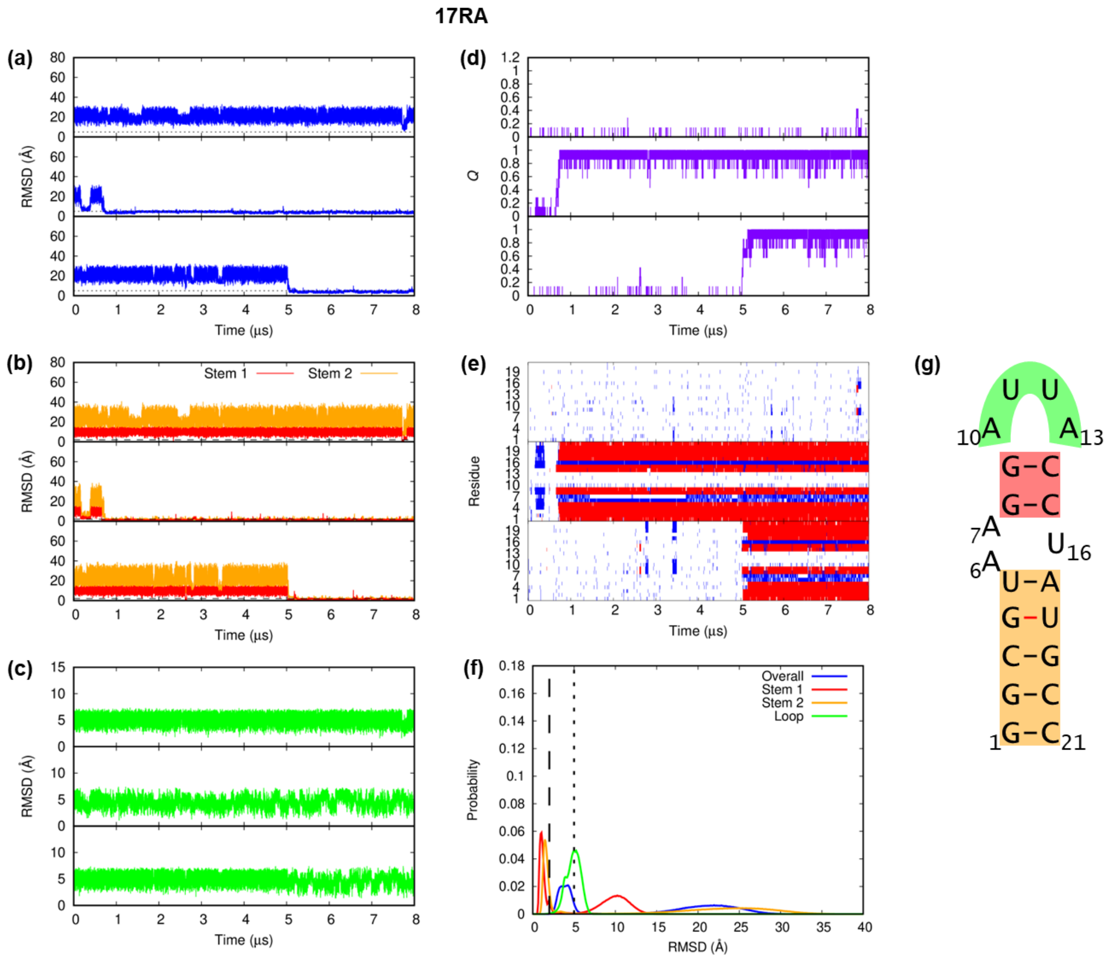

Figure S20. Trajectories of three independent molecular dynamics (MD) simulations of 17RA. (a) Root mean square deviation (RMSD) of the entire molecule. The dotted horizontal lines indicate the RMSD at 5 Å. (b) RMSD of the two stem regions, where stem 1 (red lines) and stem 2 (orange lines) represent the double helical regions directly connecting the hairpin-loop and those near the 5′ and 3′ termini of the RNA, respectively. The dashed horizontal lines indicate the RMSD at 2 Å. (c) RMSD of the hairpin-loop region. (d) The fraction of native base pairs Q. (e) Base pairs formed during simulations, where the native and non-native base pairs are represented by red and blue, respectively. (f) Probability distributions of the RMSD values for the entire molecule (blue), stem 1 (red), stem 2 (orange), and hairpin-loop (green) regions. The dashed and dotted vertical lines in the probability distribution plot indicate the RMSD values at 2 and 5 Å, respectively. (g) Secondary structure of the RNA model. Stem 1, stem 2, and hairpin-loop regions are indicated in red, orange, and green, respectively. In plots (a)–(e), three sub-panels from top to bottom correspond to the first, second, and third MD simulations, respectively.

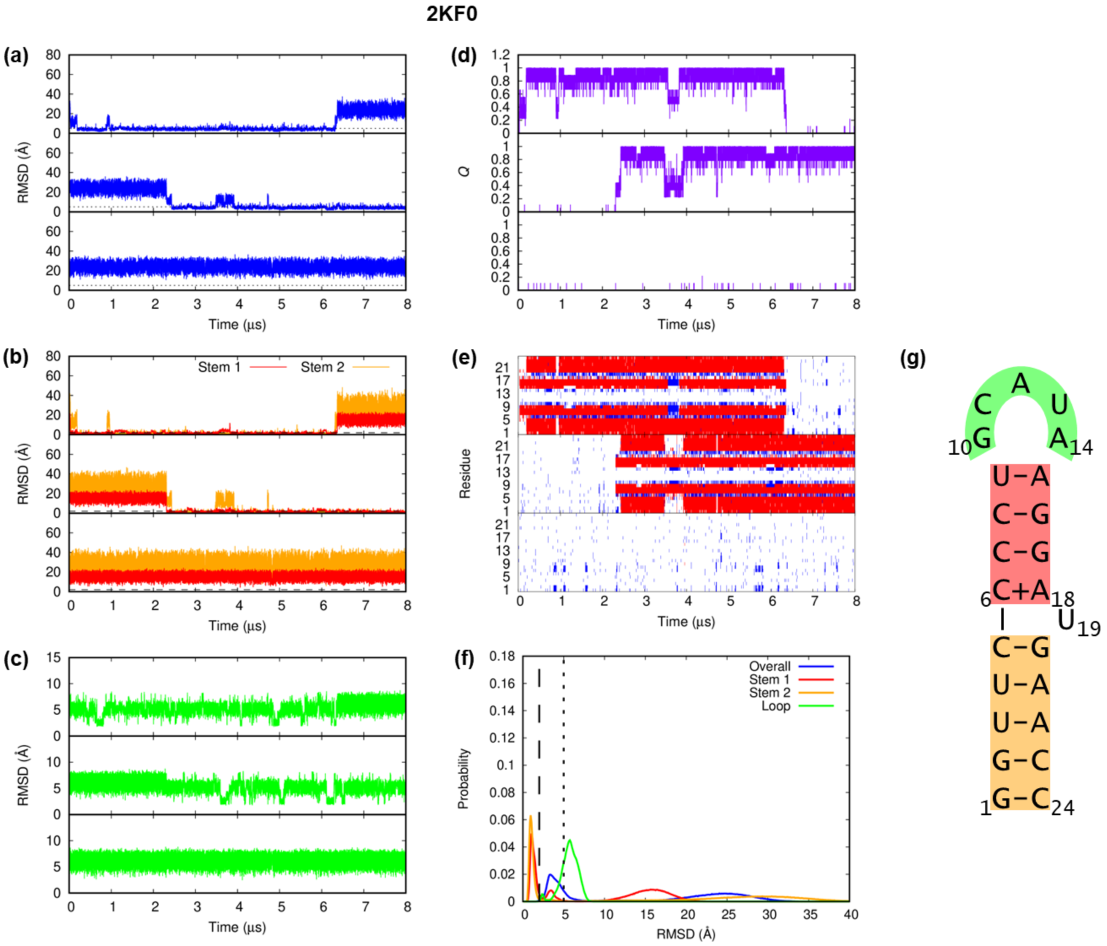

Figure S21. Trajectories of three independent molecular dynamics (MD) simulations of 2KF0. (a) Root mean square deviation (RMSD) of the entire molecule. The dotted horizontal lines indicate the RMSD at 5 Å. (b) RMSD of the two stem regions, where stem 1 (red lines) and stem 2 (orange lines) represent the double helical regions directly connecting the hairpin-loop and those near the 5′ and 3′ termini of the RNA, respectively. The dashed horizontal lines indicate the RMSD at 2 Å. (c) RMSD of the hairpin-loop region. (d) The fraction of native base pairs Q. (e) Base pairs formed during simulations, where the native and non-native base pairs are represented by red and blue, respectively. (f) Probability distributions of the RMSD values for the entire molecule (blue), stem 1 (red), stem 2 (orange), and hairpin-loop (green) regions. The dashed and dotted vertical lines in the probability distribution plot indicate the RMSD values at 2 and 5 Å, respectively. (g) Secondary structure of the RNA model. Stem 1, stem 2, and hairpin-loop regions are indicated in red, orange, and green, respectively. In plots (a)–(e), three sub-panels from top to bottom correspond to the first, second, and third MD simulations, respectively.

1ANR

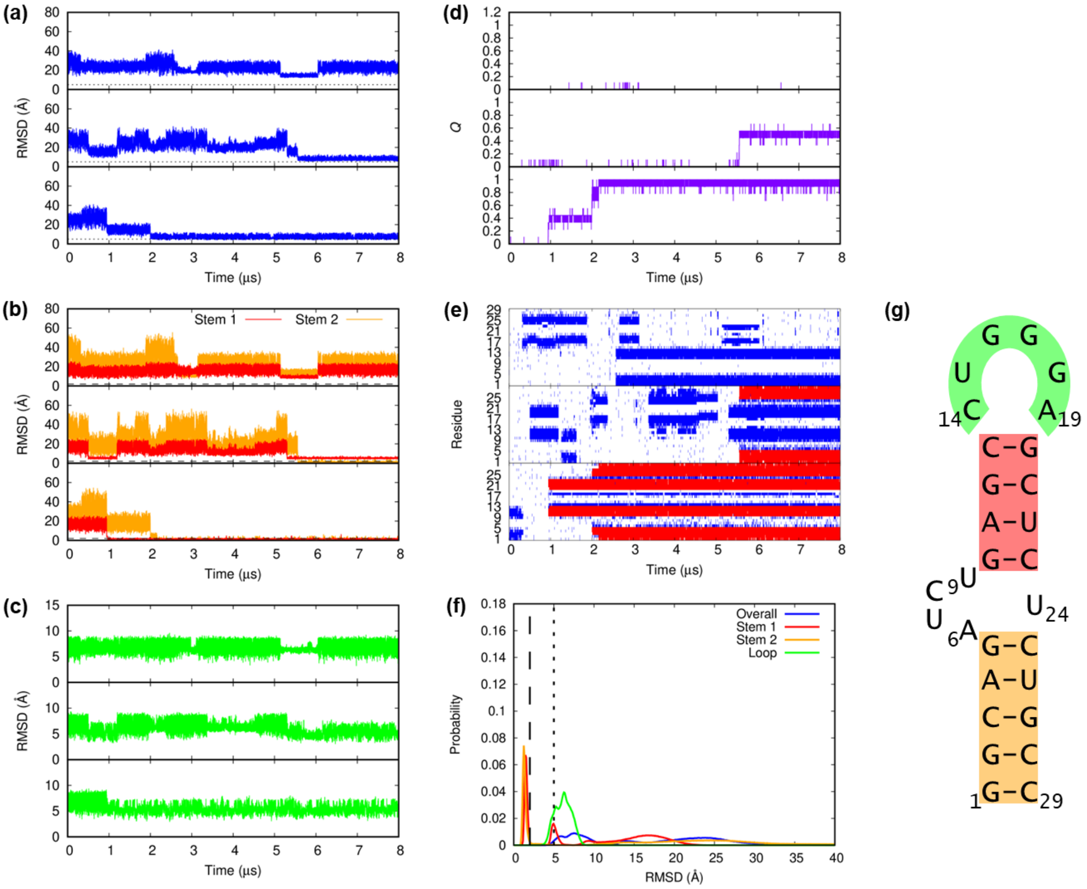

Figure S22. Trajectories of three independent molecular dynamics (MD) simulations of 1ANR. (a) Root mean square deviation (RMSD) of the entire molecule. The dotted horizontal lines indicate the RMSD at 5 Å. (b) RMSD of the two stem regions, where stem 1 (red lines) and stem 2 (orange lines) represent the double helical regions directly connecting the hairpin-loop and those near the 5′ and 3′ termini of the RNA, respectively. The dashed horizontal lines indicate the RMSD at 2 Å. (c) RMSD of the hairpin-loop region. (d) The fraction of native base pairs *Q*. (e) Base pairs formed during simulations, where the native and non-native base pairs are represented by red and blue, respectively. (f) Probability distributions of the RMSD values for the entire molecule (blue), stem 1 (red), stem 2 (orange), and hairpin-loop (green) regions. The dashed and dotted vertical lines in the probability distribution plot indicate the RMSD values at 2 and 5 Å, respectively. (g) Secondary structure of the RNA model. Stem 1, stem 2, and hairpin-loop regions are indicated in red, orange, and green, respectively. In plots (a)–(e), three sub-panels from top to bottom correspond to the first, second, and third MD simulations, respectively.

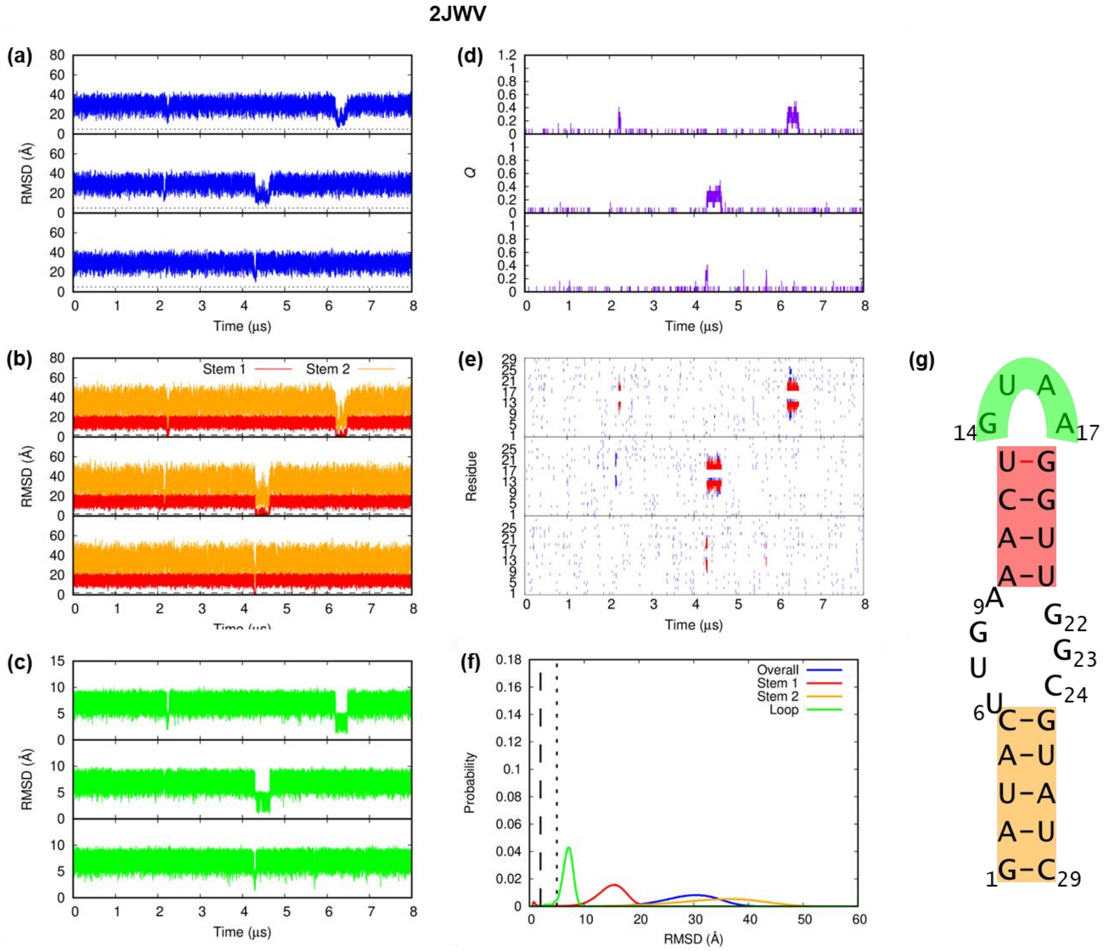

Figure S23. Trajectories of three independent molecular dynamics (MD) simulations of 2JWV. (a) Root mean square deviation (RMSD) of the entire molecule. The dotted horizontal lines indicate the RMSD at 5 Å. (b) RMSD of the two stem regions, where stem 1 (red lines) and stem 2 (orange lines) represent the double helical regions directly connecting the hairpin-loop and those near the 5′ and 3′ termini of the RNA, respectively. The dashed horizontal lines indicate the RMSD at 2 Å. (c) RMSD of the hairpin-loop region. (d) The fraction of native base pairs *Q*. (e) Base pairs formed during simulations, where the native and non-native base pairs are represented by red and blue, respectively. (f) Probability distributions of the RMSD values for the entire molecule (blue), stem 1 (red), stem 2 (orange), and hairpin-loop (green) regions. The dashed and dotted vertical lines in the probability distribution plot indicate the RMSD values at 2 and 5 Å, respectively. (g) Secondary structure of the RNA model. Stem 1, stem 2, and hairpin-loop regions are indicated in red, orange, and green, respectively. In plots (a)–(e), three sub-panels from top to bottom correspond to the first, second, and third MD simulations, respectively.

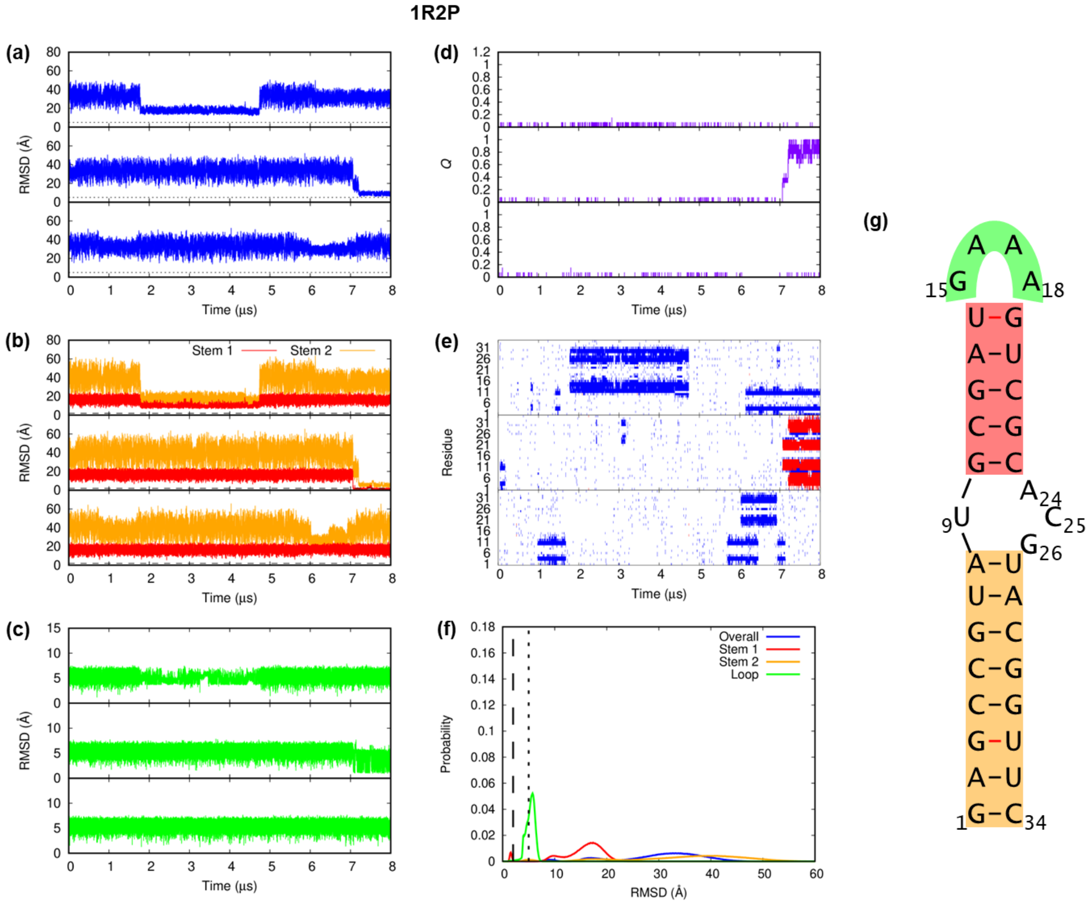

Figure S24. Trajectories of three independent molecular dynamics (MD) simulations of 1R2P. (a) Root mean square deviation (RMSD) of the entire molecule. The dotted horizontal lines indicate the RMSD at 5 Å. (b) RMSD of the two stem regions, where stem 1 (red lines) and stem 2 (orange lines) represent the double helical regions directly connecting the hairpin-loop and those near the 5′ and 3′ termini of the RNA, respectively. The dashed horizontal lines indicate the RMSD at 2 Å. (c) RMSD of the hairpin-loop region. (d) The fraction of native base pairs Q. (e) Base pairs formed during simulations, where the native and non-native base pairs are represented by red and blue, respectively. (f) Probability distributions of the RMSD values for the entire molecule (blue), stem 1 (red), stem 2 (orange), and hairpin-loop (green) regions. The dashed and dotted vertical lines in the probability distribution plot indicate the RMSD values at 2 and 5 Å, respectively. (g) Secondary structure of the RNA model. Stem 1, stem 2, and hairpin-loop regions are indicated in red, orange, and green, respectively. In plots (a)–(e), three sub-panels from top to bottom correspond to the first, second, and third MD simulations, respectively.

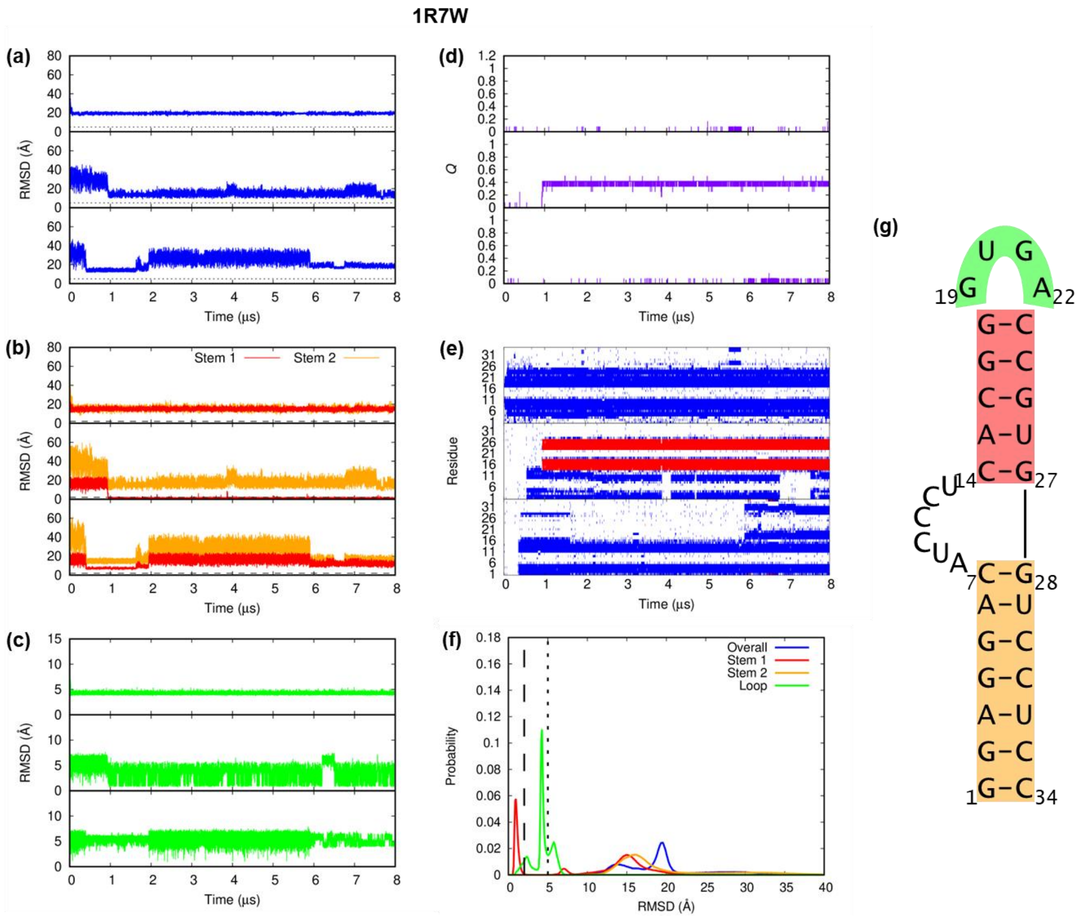

Figure S25. Trajectories of three independent molecular dynamics (MD) simulations of 1R7W. (a) Root mean square deviation (RMSD) of the entire molecule. The dotted horizontal lines indicate the RMSD at 5 Å. (b) RMSD of the two stem regions, where stem 1 (red lines) and stem 2 (orange lines) represent the double helical regions directly connecting the hairpin-loop and those near the 5′ and 3′ termini of the RNA, respectively. The dashed horizontal lines indicate the RMSD at 2 Å. (c) RMSD of the hairpin-loop region. (d) The fraction of native base pairs *Q*. (e) Base pairs formed during simulations, where the native and non-native base pairs are represented by red and blue, respectively. (f) Probability distributions of the RMSD values for the entire molecule (blue), stem 1 (red), stem 2 (orange), and hairpin-loop (green) regions. The dashed and dotted vertical lines in the probability distribution plot indicate the RMSD values at 2 and 5 Å, respectively. (g) Secondary structure of the RNA model. Stem 1, stem 2, and hairpin-loop regions are indicated in red, orange, and green, respectively. In plots (a)–(e), three sub-panels from top to bottom correspond to the first, second, and third MD simulations, respectively.

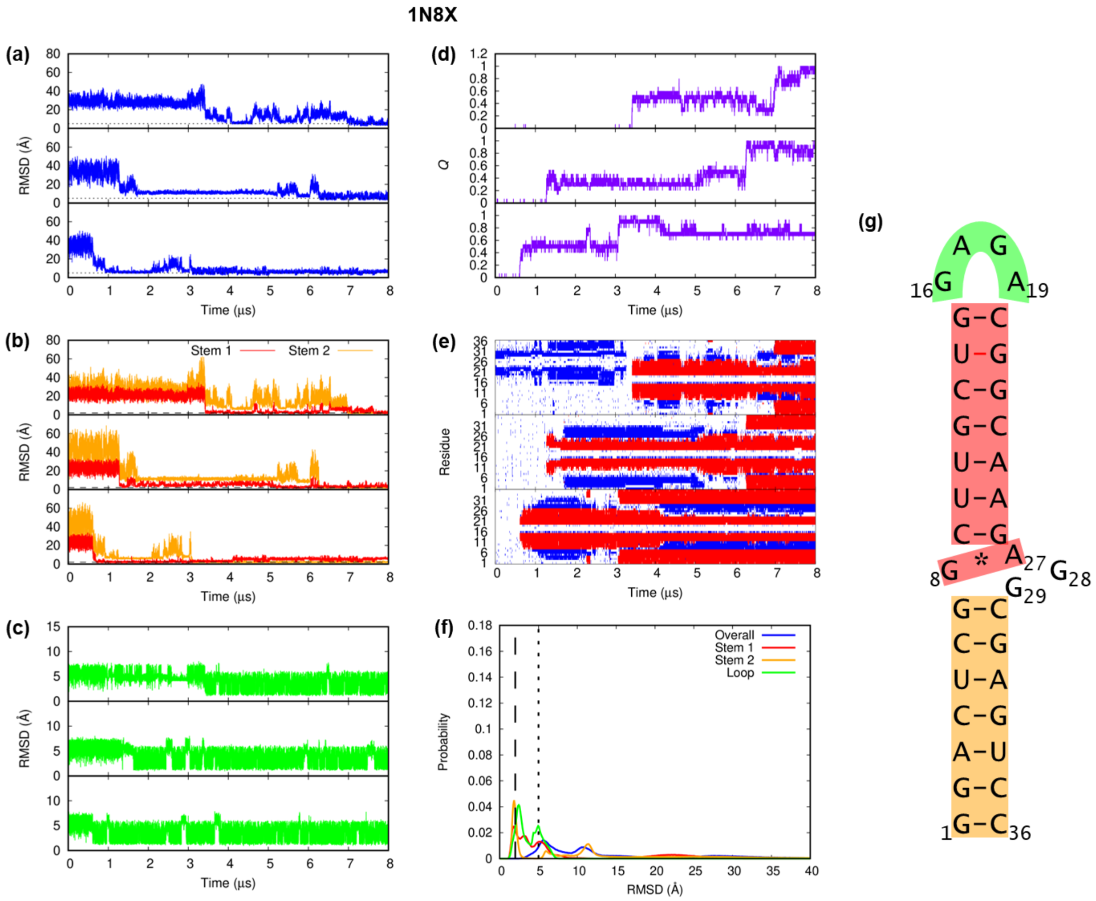

Figure S26. Trajectories of three independent molecular dynamics (MD) simulations of 1N8X. (a) Root mean square deviation (RMSD) of the entire molecule. The dotted horizontal lines indicate the RMSD at 5 Å. (b) RMSD of the two stem regions, where stem 1 (red lines) and stem 2 (orange lines) represent the double helical regions directly connecting the hairpin-loop and those near the 5′ and 3′ termini of the RNA, respectively. The dashed horizontal lines indicate the RMSD at 2 Å. (c) RMSD of the hairpin-loop region. (d) The fraction of native base pairs Q. (e) Base pairs formed during simulations, where the native and non-native base pairs are represented by red and blue, respectively. (f) Probability distributions of the RMSD values for the entire molecule (blue), stem 1 (red), stem 2 (orange), and hairpin-loop (green) regions. The dashed and dotted vertical lines in the probability distribution plot indicate the RMSD values at 2 and 5 Å, respectively. (g) Secondary structure of the RNA model. Stem 1, stem 2, and hairpin-loop regions are indicated in red, orange, and green, respectively. In plots (a)–(e), three sub-panels from top to bottom correspond to the first, second, and third MD simulations, respectively.

(a) (b)

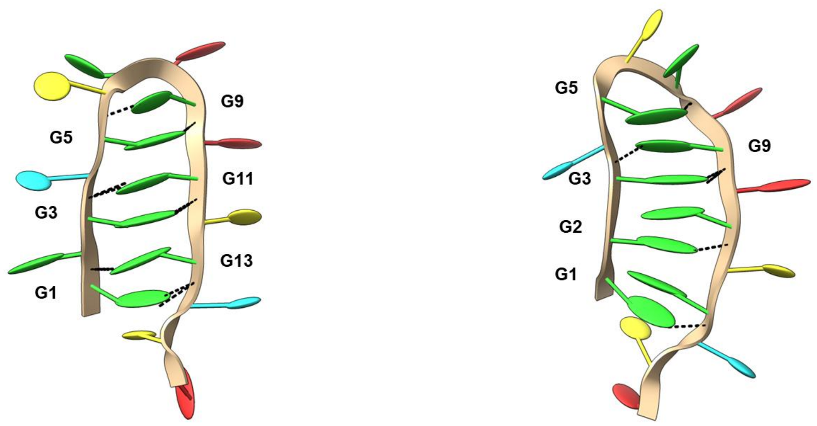

Figure S27. Centroid structures of the (a) first and (b) second clusters of 1JTW. RNA backbones are represented by ribbons. Ribose and base moieties are depicted as tubes and ellipsoids, respectively. Base coloring is defined as follows: adenine, red; cytosine, yellow; guanine, green; and uracil, cyan. Dashed black lines represent the hydrogen bonds between guanosine bases and backbone phosphates. Labeled guanosine residues are involved in the formation of hydrogen bonds.

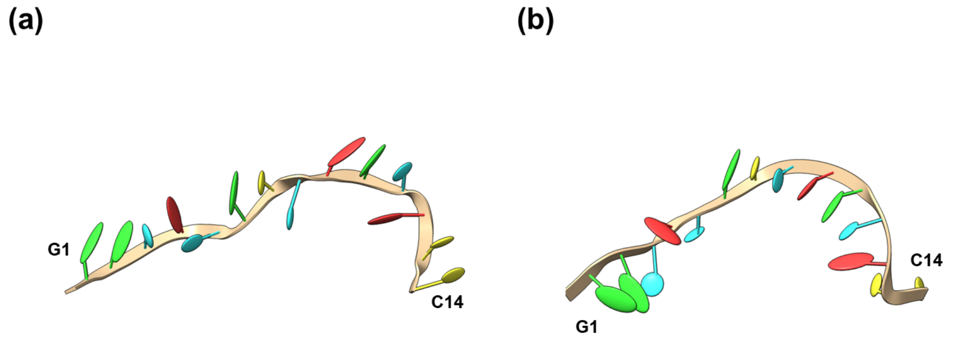

Figure S28. Centroid structures of the (a) first and (b) second clusters of 2EVY. RNA backbones are represented by ribbons. Ribose and base moieties are depicted as tubes and ellipsoids, respectively. Base coloring is defined as follows: adenine, red; cytosine, yellow; guanine, green; and uracil, cyan.

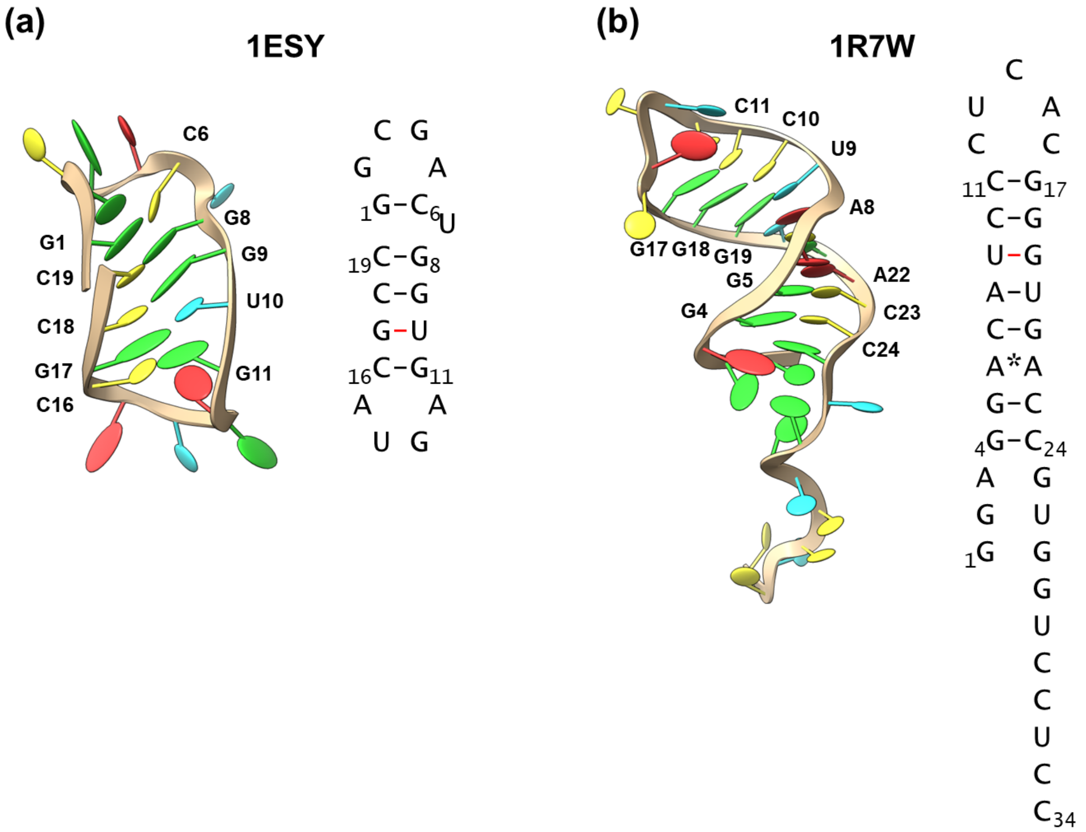

Figure S29. Misfolded structures and corresponding secondary structure diagrams forming the first clusters of (a) 1ESY and (b) 1R7W. RNA backbones are represented by ribbons. Ribose and base moieties are depicted as tubes and ellipsoids, respectively. Base coloring is defined as follows: adenine, red; cytosine, yellow; guanine, green; and uracil, cyan. In the three-dimensional structures, residues involved in base pairing are labeled, except for A6, C7, U20, and G21 in 1R7W, which are omitted for clarity. In the secondary structure diagrams, the G-C/A-U Watson-Crick and the G-U wobble base pairs are represented by black and red lines, respectively. The A6-A22 mismatch base pair in 1R7W is indicated by an asterisk (*).

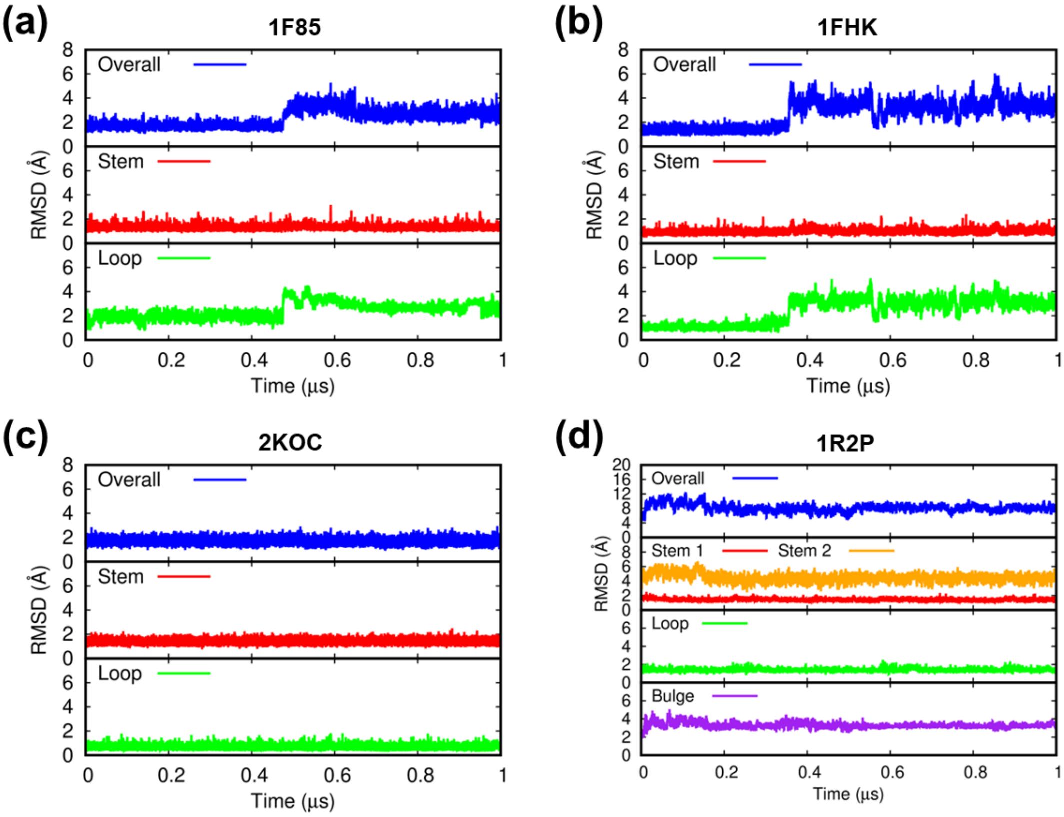

Figure S30. Trajectories of 1-μs molecular dynamics (MD) simulations for three class I stem-loops (1F85, 1FHK, and 2KOC) and one class II stem-loop (1R2P) using the DESRES-RNA force field and the TIP4P-D explicit solvent model. (a) 1F85, (b) 1FHK, (c) 2KOC, and (d) 1R2P. For the class I stem-loops (panels a–c), the three sub-panels from top to bottom show the root mean square deviation (RMSD) of non-hydrogen atoms for the entire molecule (blue), stem region (red), and loop region (green). For the class II stem-loop (panel d), RMSD trajectories are shown for five structural regions: entire molecule (blue), stem 1 (red), stem 2 (orange), loop (green), and bulge (purple).

Comparison of non-canonical and transient base pairing with NMR data

The RNA stem-loops examined in this study, 1KKA, 2LDL, 17RA, 2KF0, 1ANR, 1R2P, and 1N8X, include regions with potential base-pairing that are not detected experimentally, or whose base-pairing behavior is modulated by pH conditions or ligand binding. This section describes the analysis of the base-pairing patterns in the MD simulations using the DESRES-RNA force field and the GB-neck2 implicit solvent model, along with comparisons to NMR data.

1KKA

The U7-A11 of the molecule can potentially form a Watson–Crick base pair. In the reference NMR structure of the molecule, these residues did not form a base pair. In NMR experiments, cross-strand nuclear Overhauser effects (NOEs) indicating canonical Watson–Crick base pairing were clearly observed for the A5-U13 and U6-A12 pairs.[1] In contrast, no NOE was observed for the U7-A11 pair, but the chemical shift of the U7 imino proton was consistent with that of a Watson–Crick A-U base pair, indicating that U7-A11 may engage in transient or unstable base pairing.[1]

MD simulations using the DESRES-RNA force field and the GB-neck2 implicit solvent model showed that 18% of the structures in the first cluster formed the U7-A11 base pair. This low population of base pairing may reflect the transient nature suggested by NMR data. Conversely, the U6-A12 base pair was formed in only 48% of structures within the same cluster, which is inconsistent with the NMR experiments.

2LDL

In the NMR experiments, none of the canonical NOE patterns expected for a G-U wobble base pair were observed for the G11-U17 pair, indicating that these residues are unlikely to form a wobble base pair.[2] In contrast, MD simulations using the DESRES-RNA force field and the GB-neck2 implicit solvent model showed that 50% of the structures in the first cluster formed the G11-U17 base pair. This result is inconsistent with the NMR data.

The NMR experiments also showed that the A7-C21 pair formed a protonated A^+-C wobble base pair at pH 5.5 and that A7 titrated with a pK_a value close to 7.[2] In MD simulations using the DESRES-RNA force field and the GB-neck2 implicit solvent model, 66% of the structures in the first cluster formed an A7-C21 wobble base pair. Because the GB model implicitly assumes neutral pH conditions, this result is in reasonable agreement with the NMR experimental data.

17RA

In the bulge region, the molecule can, in principle, adopt either of two conformations, A6-U16 or A7-U16 base pairs. NMR spectral data indicated that the A6-U16 base pair and A7 bulge were the

predominant conformations at pH 6.8.[3] In contrast, MD simulations using the GB-neck2 implicit solvent model yielded 64% of structures in the first cluster with an A7-U16 base pair and 58% with an A6-U16 base pair. The implicit solvent MD simulations assuming neutral pH conditions likely overestimate the relative stability of the A7-U16 base pair compared with the A6-U16 base pair.

2KF0

The RNA molecule contains an internal loop in equilibrium between two conformations controlled by the protonation state of A18 residue.[4] At a low pH (5.7), a protonated C6-A18$^+$ wobble pair is formed. At high pH (8.0), a C6-U19 base pair is formed. The structure deposited with PDB entry 2KF0 was solved by NMR at pH 7.0, where the C6 residue appeared to form a single hydrogen bond with A18 (C6 amino-A18 N1), leaving U19 unpaired. In MD simulations using the GB-neck2 implicit solvent model, 25% of the structures in the first cluster adopted a G6-A18 base pairing, whereas 56% of the structures in the same cluster formed a C6-U19 base pair. These results suggest that the current simulation model has difficulty reproducing the relative stability differences between noncanonical C-A and C-U base pairs under pH-neutral conditions.

1ANR

The stem-loop structure of PDB entry 1ANR is derived from a *cis*-acting RNA regulatory element called TAR, which is essential for efficient transcription from the human immunodeficiency virus (HIV) promoter.[5] The molecule has a trinucleotide bulge (U7-C8-U9) near the apex of the stem-loop, which serves as the binding site for the viral regulatory protein Tat. Immediately below the bulge, the A6 and U24 residues are positioned. NMR experiments revealed an NOE cross peak between the imino proton of U24 and the H2 proton of A6 in the presence of ligands, indicating that A6-U24 formed a Watson–Crick base pair in a Tat-bound conformation.[5, 6] In contrast, in the absence of ligands, no such NOE cross peaks corresponding to the A6-U24 base pair were identified, indicating that this base pair may be unstable in the free TAR RNA structure.[5, 6]

The structures of PDB entry 1ANR were solved by NMR in the absence of ligands, where the A6 and U24 residues did not adopt a typical Watson-Crick pairing geometry. MD simulations of the 1ANR model using the DESRES-RNA force field and the GB-neck2 implicit solvent model showed that 51% of the structures in the first cluster formed the A6-U24 base pair with the Watson-Crick geometry. Given this modest base pairing probability, the simulation results are in reasonable agreement with the NMR data, reflecting the transient nature of the A6-U24 interaction in the unbound state.

1R2P

In principle, the U9 residue can form either a Watson–Crick base pair with A24 or a wobble base pair with G26. However, NMR experiments revealed no evidence of an interaction between U9 and A24

or between U9 and G26. The G26 residue adopted a *syn* conformation and flipped into the major groove of duplex stem 2, precluding canonical base pairing.[7]

In MD simulations using the DESRES-RNA force field and the GB-neck2 implicit solvent model, only 12% of the structures in the 16th cluster exhibited a U9-A24 base pair, which is qualitatively consistent with experimental observations. In contrast, 81% of the structures in the same cluster formed a U9-G26 wobble base pair. This result is inconsistent with the NMR data.

1N8X

In the NMR experiment, NOE cross peaks indicating a G8-A24 mismatched base pair were observed.[8] In MD simulations using the DESRES-RNA force field and the GB-neck2 implicit solvent model, only 9% of structures in the first cluster formed a U6-A24 base pair, which is inconsistent with the experimental results.

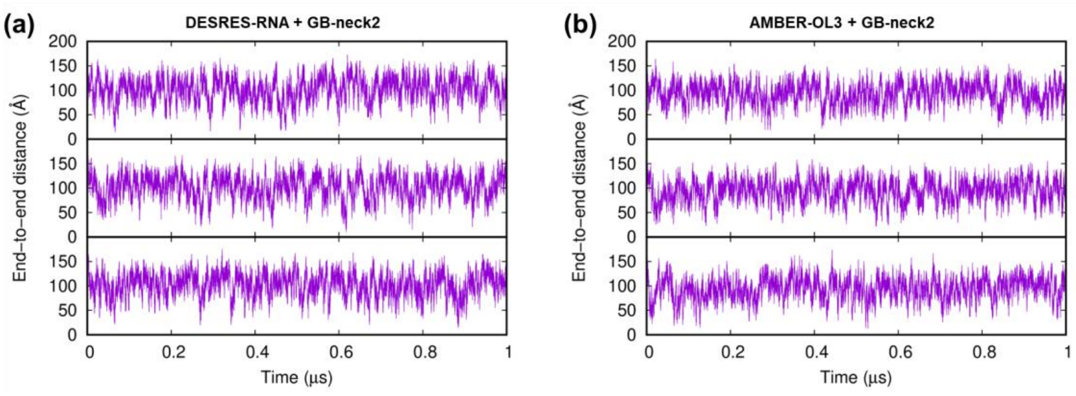

Figure S31. Trajectories of the rU_{40} single-stranded RNA end-to-end distances in three independent molecular dynamics (MD) simulations with the (a) DESRES-RNA and (b) AMBER-OL3 force fields in the GB-neck2 implicit solvent environment at a 0.15 M ionic concentration. In plots (a) and (b), the three sub-panels from top to bottom correspond to the first, second, and third MD simulations, respectively.

Explicit water molecular dynamics simulation of an RNA stem-loop in the presence of divalent magnesium ions

For the class II stem-loop 1R2P, a 1-μs molecular dynamics (MD) simulation was performed using the TIP4P-D explicit solvent model in the presence of divalent magnesium ions (Mg^{2+}) to investigate their effects on RNA structural modeling accuracy. NMR experiments have demonstrated that the RNA adopts its native structure in the absence of Mg^{2+} ions, while Mg^{2+} ions strongly bind to a minor groove shelf in the bulge.[7]

Methods

For the MD simulation, three additional Mg^{2+} and six Cl^{-} ions were added using the *tleap* module to the system containing 150 mM NaCl described in the Methods section of the main text. This corresponds to a $MgCl_2$ concentration of 10 mM. All other simulation parameters were identical to those used in the explicit solvent MD simulations in 150 mM NaCl.

Results and Discussion

Trajectories of RMSD and base pairs during 1-μs MD simulations for 1R2P using the DESRES-RNA force field and the TIP4P-D explicit solvent model in the absence and presence of magnesium ions are shown in Figure S32. In the presence of Mg^{2+} ions, the overall RMSD increased to approximately 9.0 Å. The terminal stem 2 region exhibited elevated RMSD values (~4.0 Å), whereas stem 1 remained stably formed throughout the simulation (RMSD < 2.0 Å). The loop region maintained RMSD values below 2 Å, while the bulge region fluctuated around 4.4 Å. The G26 residue consistently adopted the *syn* conformation throughout the trajectory. These trends are similar to those observed in the explicit solvent MD simulation without Mg^{2+} ions. However, in the presence of Mg^{2+}, the A8-U27 and U7-A28 base pairs were less stable and became disrupted after approximately 0.3 μs, which are different from the simulation without Mg^{2+} ions. Additionally, no base pair involving U9 was observed over the 1-μs trajectory, which differs markedly from the simulation without Mg^{2+} ions, where U9-A24 and U9-G26 base pairs were observed in 55% and 20% of the sampled structures, respectively.

Snapshots at 1 μs from explicit solvent MD simulations of 1R2P, along with the NMR structure and the lowest-RMSD centroid structure from the GB-neck2 implicit solvent MD simulation are shown in Figure S33. Overall structures obtained from the explicit solvent simulations with and without Mg^{2+} ions were similar each other and adopted slightly more compact conformations compared to the NMR structure and the GB-neck2 implicit solvent MD simulation. In the loop region, the structures obtained from explicit solvent MD simulations with and without Mg^{2+} ions were very similar, with RMSD values relative to the NMR structure remaining below 1.5 Å. In

contrast, the bulge region comprising U9, A24, C25, and G26 residues exhibited notable differences. Specifically, in the structure from the explicit solvent MD with Mg^{2+} ions, U9 adopted a flipped-out conformation, which deviated from both the NMR structure and the simulation without Mg^{2+} ions.

Three Mg^{2+} ions introduced by the AMBER *tleap* module were initial placed near the bulge region. The ions rapidly bound to the RNA near their initial positions upon initiation of the MD simulation and remained stably bound throughout the simulation. The first Mg^{2+} ion was coordinated two phosphate oxygen atoms from G10 and C11; the second ion was coordinated phosphate oxygen atoms from U7 and U8; and the third ion was coordinated by the phosphate oxygen atom of G6, the nucleobase oxygen atom O4 of U7, and the nucleobase oxygen atom O6 of G26. The binding of the third Mg^{2+} ion may contribute to the instability of the U7-U28 and a subtle conformational distortion in the bulge region.

The explicit solvent MD simulation of the class II 1R2P stem-loop in the presence of Mg^{2+} ions examined in this study demonstrated that the ions strongly bound near the bulge region, which influenced the stability of nearby base pairs and induced local conformational changes around the binding site. These results underscore the importance of appropriately incorporating Mg^{2+} ion effects into implicit solvent models to improve the fidelity of predicting RNA structure and dynamics under physiologically relevant conditions.

Nevertheless, accurately modeling Mg^{2+} ion effects remains challenging, even in explicit solvent simulations. NMR experiments of the 1R2P stem-loop revealed that resonances for A24 and C25 in the bulge region, as well as U9 on the opposite strand, were most strongly perturbed upon Mg^{2+} addition, whereas the adjacent G26 residue appeared largely unaffected.[7] Notably, the three Mg^{2+} binding sites observed in this simulation were not proximal to A24 or C25, and G26 was directly involved in ion coordination. Although the precise Mg^{2+} coordination site remains experimentally unresolved, the simulated binding sites likely differ from those inferred based on NMR perturbation patterns, highlighting the complexity of Mg^{2+}-RNA interactions and the limitations of current modeling approaches.

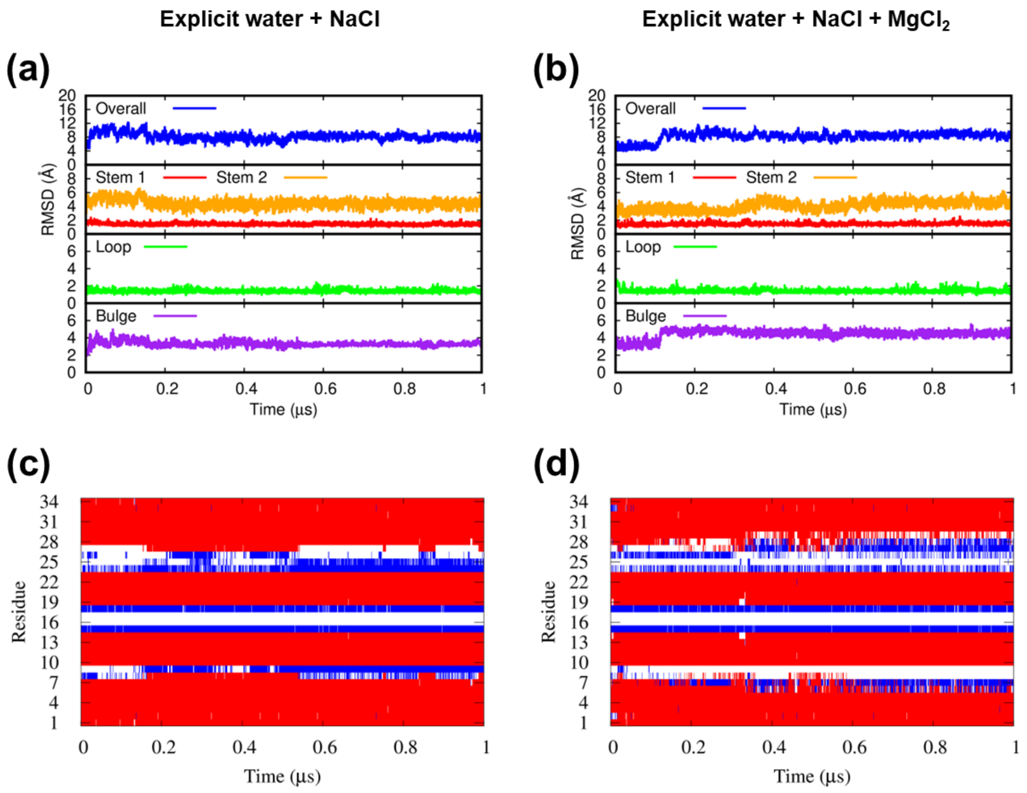

Figure S32. Trajectories of 1-μs molecular dynamics (MD) simulations for class II stem-loops 1R2P using the DESRES-RNA force field and the TIP4P-D explicit solvent model in the absence (a, c) and presence (b, d) of magnesium ions. Panels (a) and (b) show the root mean square deviation (RMSD) of non-hydrogen atoms for five structural regions: the entire molecule (blue), stem 1 (red), stem 2 (orange), loop (green), and bulge (purple). Panels (c) and (d) show base pairs formed during simulations, where the native and non-native base pairs are represented by red and blue, respectively. Panel (a) is identical to panel (d) in Figure S30.

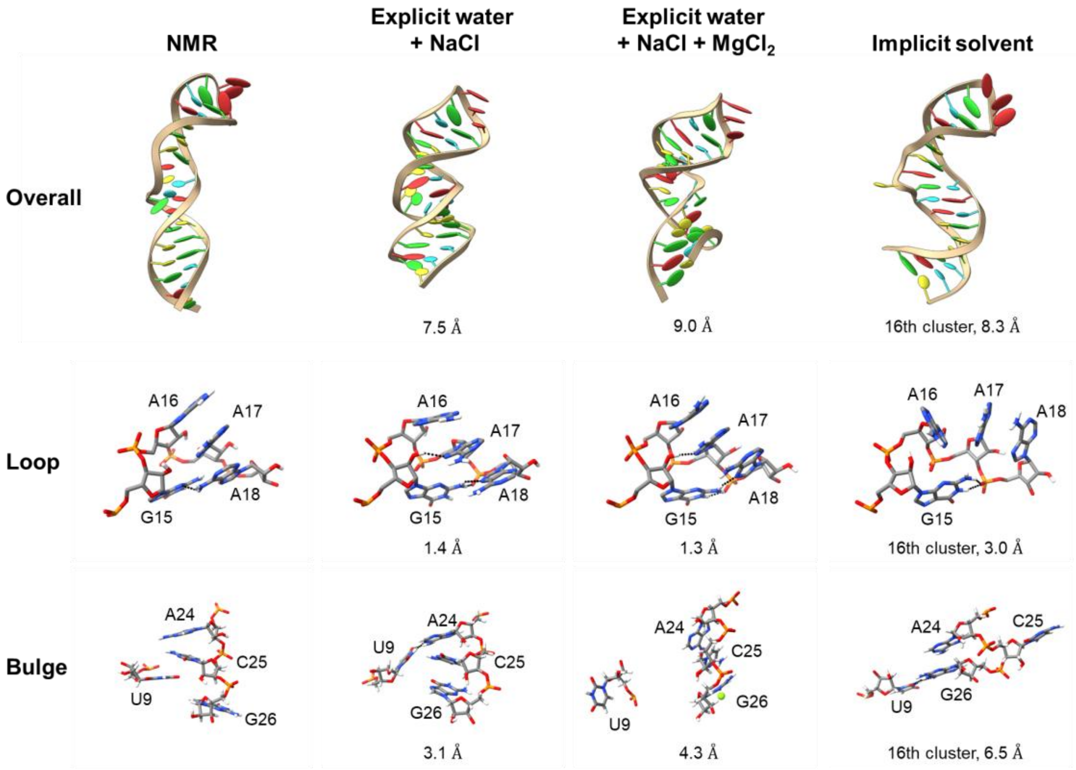

Figure S33. Comparison of overall, loop, and bulge structures of the class II 1R2P stem-loop obtained from NMR, molecular dynamics (MD) simulations with the TIP4P-D explicit solvent model in the absence and presence of magnesium ions (Mg^{2+}), and MD simulations with the GB-neck2 implicit solvent model. For the explicit solvent MD simulations, snapshots at 1 µs are shown with non-hydrogen atom root mean square deviation (RMSD) values for the corresponding structural regions. For the implicit solvent MD simulation, the centroid structure with the lowest RMSD value for the entire molecule is shown with its cluster number and corresponding RMSD value. In the overall structures (top row), RNA backbones are represented by ribbons, with ribose and base moieties depicted as tubes and ellipsoids, respectively. Base coloring is defined as follows: adenine, red; cytosine, yellow; guanine, green; and uracil, cyan. In the loop (middle row) and bulge (bottom row) structures, dashed black lines indicate hydrogen bonds. In the bulge structure obtained from the explicit solvent MD simulation with Mg^{2+} ions, a magnesium ion located near G26 is shown as a green sphere. Sub-figures for loop and bulge structures from the NMR model, the explicit solvent MD simulation without Mg^{2+} ions, and the implicit MD simulation are identical to those presented in Figure 8 of the main text.

Molecular dynamics simulations of RNA duplex formation

Models and Methods

Three non-self-complementary RNA duplexes were simulated using the DESRES-RNA force field with the GB-neck2 implicit solvent model, starting from separate complementary strands. The sequences used are CGCGG, ACUGUCA, and CGACGCAG, whose duplex structures were previously simulated by Tan et al. using the DESRES-RNA force field with the TIP4P-D explicit solvent model in a cubic box with an edge of ~55 Å containing 1 M KCl.[9] Initial structures for the implicit solvent MD simulations were generated using the *tleap* module of AMBER22, where the two complementary strands were positioned 30 Å apart along the *x*-axis. To prevent the two strands from diffusing away, a half-harmonic restraining potential was applied to the distance between the phosphorus atoms of the central residues in each strand,

$$U(r_{ij}) = \begin{cases} k(r_{ij} - R)^2 & \text{if } r_{ij} \geq R \\ 0 & \text{if } r_{ij} < R \end{cases}$$

where r_{ij} is the distance between two phosphorus atoms, R is the reference distance, and k is the force constant. In this study, R = 30 Å (approximately half the length of the simulation box used by Tan et al.) and k = 10 kcal/mol/Å^2 were used. The ionic concentration was set to 1 M. Each simulation was conducted for 2 μs, and three independent runs were performed. All other simulation parameters were identical to those used in the stem-loop folding simulations. Reference A-form duplex conformations were generated using ChimeraX.[10] The RNA A-form duplex typically adopts a C3′-endo sugar pucker conformation. The presence of C3′-endo sugar pucker was evaluated using a pseudorotation phase angle of 0 to 36° with the *cpptraj* programs.[11]

Results and Discussion

All simulation trajectories for the three RNA duplexes are shown in Figures S34–S36. Movie S3 in the Supporting Information presents the first trajectory of the CGACGCAG RNA duplex. Notably, duplex formation was observed in all simulations for each of the three sequences within the 2 μs timescale. Once formed, the duplex conformations remained stable throughout the remainder of the simulations. Non-hydrogen RMSD, base pairing, and the presence of C3′-endo sugar puckers evaluated over the last 0.5 μs of each simulation are summarized in Table S1. The average RMSD values over this period were 1.3, 3.5, and 2.3 Å for the CGCGG, ACUGUCA, and CGACGCAG duplexes, respectively. In the ACUGUCA duplex, terminal A-U base pairs (A1-U14 and A7-U8) were formed in 17%–23% of conformations during the last 0.5 μs of simulation time. In contrast, in the CGCGG and CGACGCAG duplexes, terminal G-C base pairs (C1-G10 and G5-C6 in the CGCGG, and C1-G16 and G8-C9 in the CGACGCAG) were formed in 69%–98% of the conformations during the same interval. This difference in base-pairing stability contributes to the slightly higher RMSD

values observed for the ACUGUCA model. In all three RNA duplexes, each non-terminal residue exhibited the C3′-endo sugar pucker in more than 75% of configurations during the final 0.5 μs of simulation time, indicating the adoption of a canonical A-form RNA conformation.

The simulation results for RNA duplex formation using the DESRES-RNA force field combined with the GB-neck2 implicit solvent model presented in the Supporting Information do not include investigations of the temperature or ionic strength dependence of duplex formation. Nevertheless, the results clearly demonstrate the applicability of our simulation model for studying the mechanisms of RNA duplex formation.

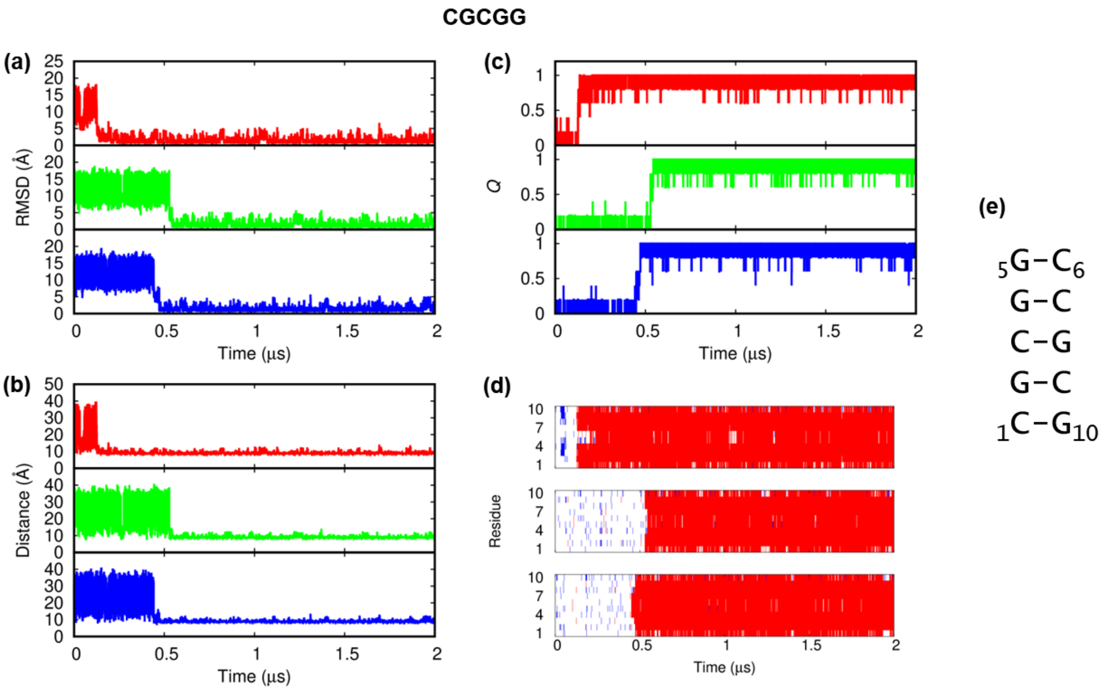

Figure S34. Trajectories of three independent molecular dynamics (MD) simulations of CGCGG RNA duplex formation. (a) Root mean square deviation (RMSD) of the entire molecule. (b) The distance between the centers of mass of the two strands. (c) The fraction of native base pairs, Q. (d) Base pairs formed during simulations. The native and non-native base pairs are represented in red and blue, respectively. (e) The RNA model consists of two strands: residues 1–5 and 6–10, which form the first and second strands, respectively. In plots (a)–(c), the three sub-panels from top to bottom (red, green, and blue lines) correspond to the first, second, and third MD simulations, respectively.

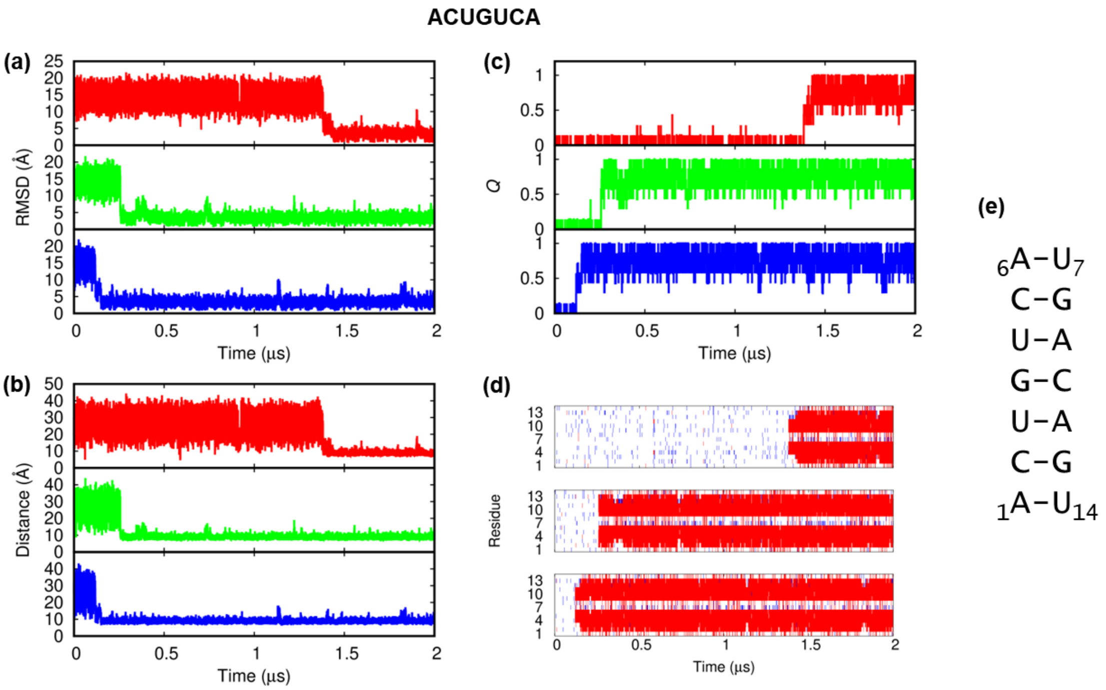

Figure S35. Trajectories of three independent molecular dynamics (MD) simulations of ACUGUCA RNA duplex formation. (a) Root mean square deviation (RMSD) of the entire molecule. (b) The distance between the centers of mass of the two strands. (c) The fraction of native base pairs, Q. (d) Base pairs formed during simulations. The native and non-native base pairs are represented in red and blue, respectively. (e) The RNA model consists of two strands: residues 1–6 and 7–14, which form the first and second strands, respectively. In plots (a)–(c), the three sub-panels from top to bottom (red, green, and blue lines) correspond to the first, second, and third MD simulations, respectively.

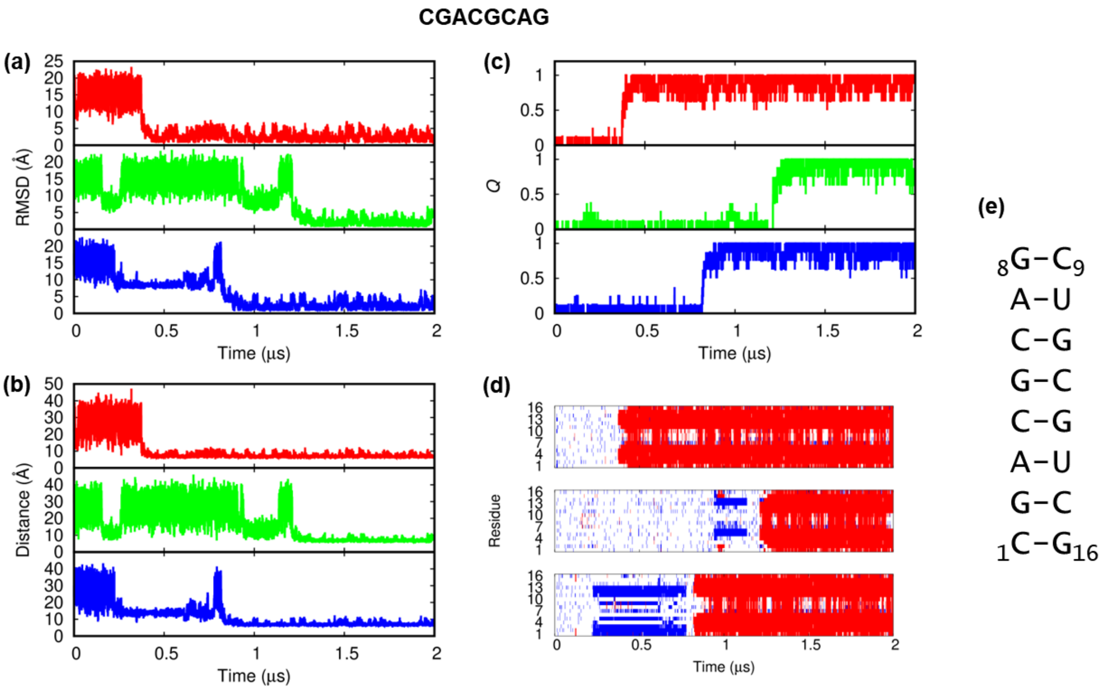

Figure S36. Trajectories of three independent molecular dynamics (MD) simulations of CGACGCAG RNA duplex formation. (a) Root mean square deviation (RMSD) of the entire molecule. (b) The distance between the centers of mass of the two strands. (c) The fraction of native base pairs, Q. (d) Base pairs formed during simulations. The native and non-native base pairs are represented in red and blue, respectively. (e) The RNA model consists of two strands: residues 1–8 and 9–16, which form the first and second strands, respectively. In plots (a)–(c), the three sub-panels from top to bottom (red, green, and blue lines) correspond to the first, second, and third MD simulations, respectively.

Table S1. Non-hydrogen atom root mean square deviation (RMSD), base pairing, and C3′-endo sugar pucker presence evaluated over the last 0.5 μs of the CGCGG, ACUCUCA, and CGACGCAG RNA duplex model simulations.

	CGCGG				ACUGUCA				CGACGCAG			
RMSD (Å)	1.3 ± 0.7				3.5 ± 1.0				2.3 ± 1.1			
Base pairs	G5-C6	98%			A7-U8	17%			G8-C9	69%		
	G4-C7	100%			C6-G9	100%			A7-U10	80%		
	C3-G8	100%			U5-A10	100%			C6-G11	100%		
	G2-C9	100%			G4-C11	100%			G5-C12	100%		
	C1-G10	85%			U3-A12	98%			C4-G13	100%		
					C2-G13	91%			A3-U14	100%		
					A1-U14	23%			G2-C15	100%		
									C1-G16	85%		
C3′-endo	G5	74%	C6	77%	A7	51%	U8	49%	G8	66%	C9	67%
sugar pucker	G4	88%	C7	90%	C6	82%	G9	79%	A7	88%	U10	80%
	C3	91%	G8	88%	U5	88%	A10	88%	C6	90%	G11	86%
	G2	85%	C9	89%	G4	87%	C11	88%	G5	86%	C12	91%
	C1	72%	G10	68%	U3	89%	A12	90%	C4	90%	G13	87%
					C2	76%	G13	86%	A3	89%	U14	88%
					A1	55%	U14	41%	G2	86%	C15	86%
									C1	74%	G16	70%
									G8	66%	C9	67%

Descriptions of simulation movies

Movie S1. Folding of a 1SZY class I stem-loop observed in the first molecular dynamics simulation using the DESRES-RNA force field and the GB-neck2 implicit solvent model. This movie visualizes the trajectory from 2.42 to 2.5 μs, in which snapshots at 40 ps intervals are played at 25 frames per second. The time evolutions of the root mean square deviation for the stem region and formed base pairs in this simulation are shown in Figure 6.

Movie S2. Folding of a 1ANR class II stem-loop observed in the third molecular dynamics simulation using the DESRES-RNA force field and the GB-neck2 implicit solvent model. This movie visualizes the trajectory from 1 to 4 μs, in which snapshots at 2 ns intervals are played at 25 frames per second. The time evolutions of the root mean square deviation for the stem regions and formed base pairs in this simulation are shown in Figure 7.

Movie S3. Formation of a CGACGCAG RNA duplex in the first molecular dynamics simulation using the DESRES-RNA force field and the GB-neck2 implicit solvent model. This movie visualizes the trajectory from 350 to 400 ns, in which snapshots at 0.4 ns intervals are played at 25 frames per second. The time evolutions of the root mean square deviation, distance between centers of mass of two strands, fraction of native base pairs, and formed base pairs in this simulation are shown in Figure S36.

Input files used for AMBER MD simulations

```
# tleap.in (Input file for tleap)
source leaprc.RNA.Shaw
# The following sequence is for 1R4H as an example.
rna = sequence { G5 G G C A A G C C C3 }
set default PBradii mbondi3
savepdb rna rna.pdb
saveAmberParm rna rna.parm7 rna.rst7
quit
```

```
# min.in (Input file for minimization)
Minimization with GB-neck2
&cntrl
   imin = 1,
   maxcyc = 1000, ncyc = 500, ntpr = 50,
   ntb = 0, ntp = 0, igb = 8, saltcon = 0.15, dielc = 1.0, intdiel = 1.0,
   cut = 999.0, rgbmax = 999.0,
&end
```

```
# md.in (Input file for MD simulation)
1 microsecond MD simulation with implicit solvent
&cntrl
  imin = 0, ntx = 1, irest = 0,
  nstlim = 500000000, dt = 0.002,
  ntpr = 1000, ntwx = 1000, ntwr = 10000000,
  ntb = 0, ntp = 0,
  ntt = 3, gamma_ln = 1.0, ig = -1, temp0 = 298.0, tempi = 298.0,
  ntc = 2, ntf = 2, cut = 999.0,
  igb = 8, saltcon = 0.15, rgbmax = 999.0, dielc = 1.0, intdiel = 1.0,
&end
```

SI References

(1) Cabello-Villegas, J.; Winkler, M. E.; Nikonowicz, E. P. Solution conformations of unmodified and $A_{37}N^6$-dimethylallyl modified anticodon stem-loops of *Escherichia coli* $tRNA^{Phe}$. *J. Mol. Biol.* **2002**, *319* (5), 1015-1034. DOI: 10.1016/S0022-2836(02)00382-0

(2) Levengood, J. D.; Rollins, C.; Mishler, C. H. J.; Johnson, C. A.; Miner, G.; Rajan, P.; Znosko, B. M.; Tolbert, B. S. Solution structure of the HIV-1 exon splicing silencer 3. *J. Mol. Biol.* **2012**, *415* (4), 680-698. DOI: 10.1016/j.jmb.2011.11.034

(3) Smith, J. S.; Nikonowicz, E. P. NMR structure and dynamics of an RNA motif common to the spliceosome branch-point helix and the RNA-binding site for phage GA coat protein. *Biochemistry* **1998**, *37* (39), 13486-13498. DOI: 10.1021/bi981558a

(4) Venditti, V.; Clos, L.; Niccolai, N.; Butcher, S. E. Minimum-energy path for a U6 RNA conformational change involving protonation, base-pair rearrangement and base flipping. *J. Mol. Biol.* **2009**, *391* (5), 894-905. DOI: 10.1016/j.jmb.2009.07.003

(5) Aboulela, G.; Karn, J.; Varani, G. Structure of HIV-1 TAR RNA in the absence of ligands reveals a novel conformation of the trinucleotide bulge. *Nucleic Acids Res.* **1996**, *24* (22), 4598-4598.

(6) Aboulela, F.; Karn, J.; Varani, G. The structure of the human-immunodeficiency-virus type-1 TAR RNA reveals principles of RNA recognition by Tat protein. *J. Mol. Biol.* **1995**, *253* (2), 313-332. DOI: 10.1006/jmbi.1995.0555

(7) Sigel, R. K. O.; Sashital, D. G.; Abramovitz, D. L.; Palmer, A. G.; Butcher, S. E.; Pyle, A. M. Solution structure of domain 5 of a group II intron ribozyme reveals a new RNA motif. *Nat. Struct. Mol. Biol.* **2004**, *11* (2), 187-192. DOI: 10.1038/nsmb717

(8) Lawrence, D. C.; Stover, C. C.; Noznitsky, J.; Wu, Z. R.; Summers, M. F. Structure of the intact stem and bulge of HIV-1 ψ-RNA stem-loop SL1. *J. Mol. Biol.* **2003**, *326* (2), 529-542. DOI: 10.1016/S0022-2836(02)01305-0

(9) Tan, D.; Piana, S.; Dirks, R. M.; Shaw, D. E. RNA force field with accuracy comparable to state-of-the-art protein force fields. *Proc. Natl. Acad. Sci. U. S. A.* **2018**, *115* (7), E1346-E1355. DOI: 10.1073/pnas.1713027115

(10) Meng, E. C.; Goddard, T. D.; Pettersen, E. F.; Couch, G. S.; Pearson, Z. J.; Morris, J. H.; Ferrin, T. E. UCSF ChimeraX: Tools for structure building and analysis. *Protein Sci.* **2023**, *32* (11). DOI: 10.1002/pro.4792

(11) Linzer, J. T.; Aminov, E.; Abdullah, A. S.; Kirkup, C. E.; Ventura, R. I. D.; Bijoor, V. R.; Jung, J.; Huang, S. P.; Tse, C. G.; Toucet, E. A.; Onghai, H. P.; Ghosh, A. P.; Grodzki, A. C.; Haines, E. R.; Iyer, A. S.; Khalil, M. K.; Leong, A. P.; Neuhaus, M. A.; Park, J.; Shahid, A.; Xie, M.; Ziembicki, J. M.; Simmerling, C.; Nagan, M. C. Accurately modeling RNA stem-loops in an implicit solvent environment. *J. Chem. Inf. Model.* **2024**, *64* (15), 6092-6104. DOI: 10.1021/acs.jcim.4c00756
